# Photonic-electronic arbitrary-waveform generation using quadrature multiplexing and active optical-phase stabilization

**DOI:** 10.1038/s41467-025-61564-w

**Published:** 2025-09-18

**Authors:** Christoph Füllner, Alban Sherifaj, Thomas Henauer, Dengyang Fang, Daniel Drayss, Lennart Schmitz, Tobias Harter, Tilahun Z. Gutema, Thomas Zwick, Wolfgang Freude, Sebastian Randel, Christian Koos

**Affiliations:** 1 Institute of Photonics and Quantum Electronics (IPQ), Karlsruhe Institute of Technology (KIT), Karlsruhe, Germany; 2 Institute of Radio Frequency Engineering and Electronics (IHE), Karlsruhe Institute of Technology (KIT), Karlsruhe, Germany; 3 Teragear GmbH, Karlsruhe, Germany; 4 Institute of Microstructure Technology (IMT), Karlsruhe Institute of Technology (KIT), Eggenstein-Leopoldshafen, Germany

**Keywords:** Fibre optics and optical communications, Optoelectronic devices and components

## Abstract

Generation of electrical waveforms with bandwidths of 100 GHz or more is key to many applications in science and industry, comprising high-speed communications, radar, or test and measurement equipment. However, while conventional digital-to-analog converters based on electronic circuits still represent the technological mainstay for broadband waveform generation, further bandwidth scaling comes with a series of challenges related to circuit design and implementation, packaging, and system integration. In this paper, we show that photonic-electronic signal-processing techniques may overcome these limitations. We demonstrate a photonic-electronic waveform generator that exploits quadrature multiplexing in the optical domain in combination with phase-stabilized coherent down-conversion to the electrical domain. In a proof-of-concept experiment, we generate electrical multi-level data signals at symbol rates up to 200 GBd at quality levels that can already compete with best-in-class electronic systems. We believe that our concept opens an attractive path to waveforms generation at bandwidths beyond the limitations of current microelectronics, leveraging advanced photonic integration technologies that are currently being developed.

## Abstract (web summary)

The authors propose and demonstrate the concept of photonic-electronic arbitrary-waveform generation, overcoming the bandwidth limitations of all-electronic systems. The idea is to exploit quadrature multiplexing of optical waveforms and opto-electronic conversion by phase-stabilized coherent detection.

## Introduction

Broadband arbitrary-waveform generation is a fundamental technology that is widely used in science and industry, be it for high-speed optical^1–8^ and wireless^9^ communications, for test and measurement of high-speed electronics, or for investigation of ultra-short events in scientific experiments^10^. High-speed digital-to-analog converters (DACs) based on advanced electronic circuits currently represent the technological mainstay for broadband waveform generation. Using 16-nm CMOS nodes, DACs with bandwidth up to 45 GHz have been demonstrated already a few years ago^3,5^. More recently, high-speed CMOS application-specific integrated circuits (ASICs) based on 3-nm, 5-nm, and 7-nm technology have been announced in the context of high-speed optical communications^11–15^, featuring DAC bandwidths of 60 GHz or more. For a given structure size, BiCMOS circuits can reach much higher speeds compared to their CMOS counterparts, leading to usable DAC bandwidths of up to 65 GHz demonstrated on a 55-nm BiCMOS node^4^. However, further scaling the bandwidth of individual DAC interfaces is challenging due to bandwidth limitations of the underlying circuit elements, the complexity of associated packaging and impedance matching approaches^16^, and increased transmission line losses^17^. To overcome these limitations, several multiplexing techniques have been proposed in the past^18,19^. They all have in common that the bandwidth-limited analog outputs of multiple DACs are actively combined to form a single high-speed output. More specifically, bandwidths of up to 80 GHz have been achieved by time interleaving of parallel DACs, and 100 GHz were reached by spectral stitching^20–22^. Still, all of these fully-electronic concepts require broadband RF multiplexers or RF mixer circuits that are challenging to design, to fabricate, and to integrate into multi-chip RF systems.

In this paper, we introduce optical quadrature multiplexing as an alternative approach for broadband arbitrary-waveform generation, exploiting photonic-electronic signal-processing schemes to effectively double the bandwidth of electronic DACs^23,24^. Specifically, the approach relies on in-phase (I) and quadrature (Q) modulation of an optical carrier using a pair of well-defined electronic drive signals, which leads to an optical IQ waveform with uncorrelated sidebands that covers twice the bandwidth of the underlying electronic DACs. The optical waveform can then be down-converted to a corresponding ultra-broadband electric waveform using a high-bandwidth balanced photodetector (BPD) in combination with a local oscillator (LO) tone tuned to the spectral edge of the optical IQ signal, thereby effectively leading to the formation of an optical single-sideband (SSB) signal^25–29^ at the BPD input. A key element of this approach is a precise control of the relative phase between the IQ signal and the LO tone, which is accomplished through a closed-loop active phase stabilization scheme. We demonstrate the viability of the approach by implementing a proof-of-concept photonic-electronic arbitrary-waveform generator (PE-AWG), which relies on a pair of conventional electronic DACs with an individual bandwidth of approximately 50 GHz and which offers a 100 GHz overall bandwidth. We use the system for generating two-level, four-level, and eight-level pulse-amplitude modulation signals (PAM2, PAM4, and PAM8) with symbol rates of up to 200 GBd, and we analyze and benchmark the resulting signal quality via well-established metrics for communication signals such as the signal-to-noise-and-distortion ratio (SNDR) and the bit-error ratio (BER). We find that our proof-of-concept system can already compete with best-in-class commercially available benchtop-type waveform generators. As an application example, the PE-AWG system is finally used to drive a high-speed Mach-Zehnder modulator (MZM) for intensity modulation of an optical carrier, resulting in on-off-keying (OOK) and PAM4 signals with symbol rates of up to 200 GBd and 190 GBd, respectively, which are transmitted over a 10.5-km-long fiber link. To the best of our knowledge, the 100 GHz-wide waveforms generated in our experiments represent the highest-bandwidth electric data signals that were synthesized so far by a photonic-electronic system. In combination with recently demonstrated phase-stabilized spectral stitching of optical waveforms^30^, the proposed scheme paves a path towards efficient bandwidth scaling of waveform generators with linearly increasing effort, exploiting massive parallelization of low-speed DAC arrays. Our approach may leverage the tremendous progress of broadband electro-optic devices such as modulators^31–34^ and ultra-high-speed photodetectors^35–39^.

This paper is structured as follows: In Subsection “Photonic-electronic arbitrary-waveform generator (PE-AWG) concept” of Section “Results”, we explain the general concept of the proposed PE-AWG scheme. The subsequent subsection “Active optical-phase stabilization” explains the underlying closed-loop active optical-phase stabilization, which is key to the generation of well-defined time-domain waveforms. In Subsection “Digital synthesis of IQM drive signals”, we describe the digital synthesis of the IQM drive signals that lead to the desired target waveform at the PE-AWG output, and Subsection “Bandwidth scaling and spectral stitching in the optical domain” presents an approach to further bandwidth scaling by using spectral stitching techniques. In Subsection “Experimental implementation”, we present the experimental proof-of-concept demonstration, while the benchmarking of the resulting signal quality is discussed in Subsections “Characterization and performance benchmarking” and “Symbol rate sweep”. In the last subsection of Section “Results” labelled “Application example: IM/DD fiber transmission experiment” we describe the results achieved when employing our PE-AWG as a signal source in an optical transmission experiment. In Section “Discussion” we discuss a roadmap for the PE-AWG including potential improvements, photonic integration, and possible use cases in more detail and summarize our findings.

## Results

### Photonic-electronic arbitrary-waveform generator (PE-AWG) concept

**Fig. 1:**
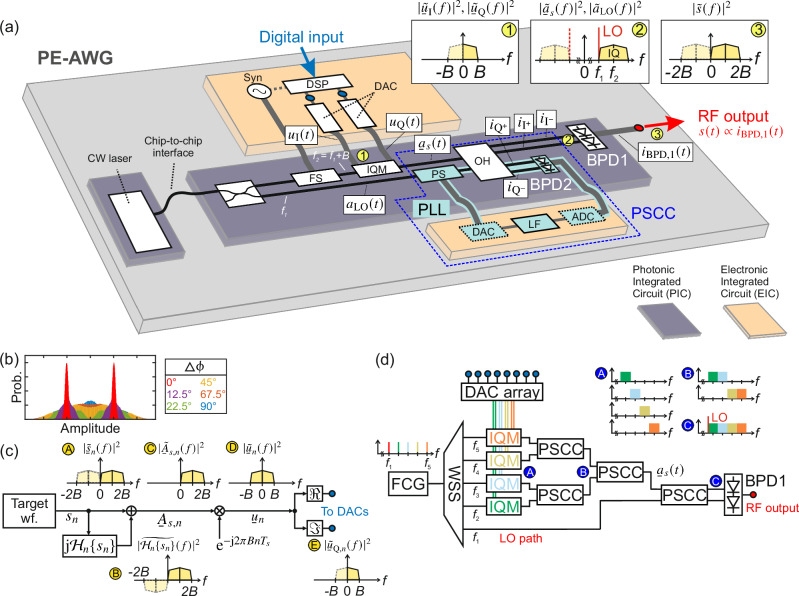
Concept of a photonic-electronic arbitrary waveform generator (PE-AWG) relying on quadrature multiplexing and an active optical phase stabilization in a phase-locked loop (PLL). **a** Visionary illustration of a chip-scale hybrid integrated PE-AWG comprising photonic integrated circuits (PICs) and electronic integrated circuits (EICs) intended to give an idea of what a miniaturized PE-AWG system could look like. Two phase-locked optical tones at frequencies *f*_1_ and *f*_2_ are derived from a continuous-wave (CW) laser. After coupling to the main PIC, the light emitted by the CW laser is split into two portions. One portion is frequency-shifted (FS) by *B* = *f*_2_ − *f*_1_ and acts as a carrier for an optical IQ waveform; the other one serves as a local oscillator (LO) tone for heterodyne down-conversion in a high-bandwidth balanced photodetector (BPD1). The drive signals for the IQ modulator (IQM) are provided by two time-synchronized DAC modules with bandwidth *B* each. A feedback loop (turquoise) in a phase-stabilized coherent combiner (PSCC) compensates the unwanted phase deviation Δ*ϕ* between the optical IQ waveform and the LO tone. The insets show spectra at positions ① to ③ in Subfigure (a) and illustrate the basic principle of quadrature multiplexing in the frequency domain, where the tilde denotes the Fourier transforms of the respective time-domain quantities. In these spectral plots, dashed lines refer to spectral components at negative frequencies that are simply the complex conjugate of their positive-frequency counterparts. ① Real-valued electric drive signal as fed to the I and Q ports of the IQM. ② Optical IQ signal covering a bandwidth of 2*B* along with the LO tone located at the edge of the signal spectrum. ③ Real-valued waveform with bandwidth 2*B* obtained at the output of BPD1 after coherent down-conversion. **b** Histogram of a PAM2 signal at the PE-AWG output for various phase offsets Δ*ϕ*. A non-zero phase offset introduces severe distortions to the generated electrical waveform, and the histogram deteriorates to the point of being unrecognizable as a PAM2 signal. **c** Digital synthesis of the drive signals $\Re \{\underline{u}(t)\}$ and $\Im \{\underline{u}(t)\}$ for the IQM. A discrete-time version of the target waveform *s*_*n*_ covering a bandwidth 2*B* is generated, Inset Ⓐ. Next, its Hilbert transform, Inset Ⓑ, is added as an imaginary part, leading to an elimination of the negative-frequency components, Inset Ⓒ. The analytic signal ${\underline{A}}_{s,n}$ is then frequency-shifted to be centered around zero frequency, Inset Ⓓ, and the real and imaginary parts of the resulting digital signal ${\underline{u}}_{n}$ are fed to the two DAC modules with bandwidth *B*, Inset Ⓔ. **d** Vision of an ultra-broadband PE-AWG that combines the idea of quadrature multiplexing with the generation of broadband optical waveforms by feedback-stabilized stitching of spectrally sliced tributary signals as illustrated in the Insets Ⓐ to Ⓒ^30^. An illustrative example with *N* = 4 tributaries is sketched. Each of the phase-stabilized coherent combiners (PSCC) consists of an optical hybrid (OH) and a PLL for phase stabilization. For the PSCCs with a single output only, the output labeled ‘I^−^’ in (a) is unused.

Figure 1a shows a visionary illustration of a fully integrated ultra-broadband photonic-electronic arbitrary-waveform generator (PE-AWG) exploiting the concept of quadrature multiplexing. The figure is intended to give an idea of what a miniaturized PE-AWG system could look like based on technologies that are in principle available already today. The system comprises multiple interconnected photonic integrated circuits (PICs) and electronic integrated circuits (EICs). Electrical connections may be realized with conventional metal wirebonds and photonic wirebonds^40,41^ are a possibility to efficiently connect the PICs but alternative coupling techniques are feasible as well^42^. The generation of the broadband electrical target waveform *s*(*t*) at the RF ouput relies on multiplexing the output signals of a pair of independent synchronized electronic DACs via an IQ modulator (IQM) in the optical domain. To this end, real-valued I and Q signals, *u*_I_(*t*) and *u*_Q_(*t*), each having a single-sided bandwidth *B* in baseband, see Inset ① of Fig. 1a, are modulated onto an optical carrier. In Inset ① and in all further spectral plots, dashed lines refer to spectral images at negative frequencies that are simply the complex conjugate of their positive-frequency counterparts and thus carry only redundant information. The superposition of the optical I and Q signals, also referred to as the optical IQ signal, exhibits fully uncorrelated spectral components over its entire spectral width 2*B*, see Inset ② - as opposed to a pure amplitude modulation of an optical carrier, for which the upper and lower sidebands are correlated. We indicate the independence of the upper and lower sidebands of complex-valued optical signals by solid lines in the spectral plots. Phase-stabilized heterodyne down-conversion of such optical IQ signals by means of a local oscillator (LO) located at the edge of the optical signal spectrum finally allows for generating real-valued electrical waveforms having twice the bandwidth of the employed DACs, see Inset ③. In this case, the optical IQ signal can be interpreted as a single-sideband signal with respect to the LO tone at the BPD input.

Key to the signal generation and phase-stabilized coherent down-conversion are two frequency- and phase-locked laser tones at frequencies *f*_1_ and *f*_2_ = *f*_1_ + *B*, where 2*B* is the bandwidth covered by the electrical target waveform. One tone acts as an optical carrier onto which the IQ signal is modulated, while the other one is used as the LO for down-conversion. These tones can either be provided by an optical frequency-comb generator (FCG), which is particularly attractive if the scheme is complemented by spectral stitching to increase the bandwidth of the optical IQ signal^30^, or by a single continuous-wave (CW) laser in combination with a frequency shifter (FS)^43^, that derives the tone at *f*_2_ from the one at *f*_1_ by imposing a spectral shift of *f*_shift_ = *B*, see Fig. 1a. The RF signal for driving the frequency shifter is provided by a synthesizer (Syn) that is synchronized with the digital signal processing (DSP) unit and the two DACs as indicated by the dotted line in Fig. 1a. The optical carrier at *f*_2_ is then modulated in the IQ modulator (IQM), which is driven by two real-valued drive signals *u*_I_(*t*) and *u*_Q_(*t*). The resulting optical IQ signal ${\underline{a}}_{s}(t)=\left({u}_{{{{\rm{I}}}}}(t)+{{{\rm{j}}}}{u}_{{{{\rm{Q}}}}}(t)\right)\exp \left({{{\rm{j}}}}\left(2\pi {f}_{2}t+{\phi }_{s}(t)\right)\right.$ is fed to a 90° optical hybrid (OH) and combined with the LO tone ${a}_{{{{\rm{LO}}}}}(t)={\Re} \left\{{A}_{{{{\rm{LO}}}}}\,\exp \left({{{\rm{j}}}}\left(2\pi {f}_{1}t+{\phi }_{{{{\rm{LO}}}}}(t)\right)\right)\right\}$ at *f*_1_ for heterodyne down-conversion in the high-bandwidth balanced photodetector (BPD1). In these relations, the quantities *ϕ*_*s*_(*t*) and *ϕ*_LO_(*t*) represent slow phase variations of the signal carrier and the LO tone that are, e.g., caused by thermal drift or mechanical vibrations of the waveguides and the frequency comb generators. The LO tone is positioned exactly at the edge of the data spectrum, *f*_2_ = *f*_1_ + *B*, see Inset ② of Fig. 1a, and we can re-write the optical IQ signal as ${\underline{a}}_{s}(t)={\underline{A}}_{s}(t)\exp \left({{{\rm{j}}}}\left(2\pi {f}_{1}t+{\phi }_{s}(t)\right)\right)$ where the complex-valued envelope ${\underline{A}}_{s}(t)=\left({u}_{{{{\rm{I}}}}}(t)+{{{\rm{j}}}}{u}_{{{{\rm{Q}}}}}(t)\right)\exp \left({{{\rm{j}}}}2\pi Bt\right)$ now refers to the LO tone at frequency *f*_1_. Assuming ideal balancing and hence full elimination of the common-mode terms at the BPD output, we obtain the real-valued photocurrent derived from the ‘I^+^’ and ‘I^−^’ port of the OH, see Section S1 of Supplement 1 for details,

1 $${i}_{{{{\rm{BPD}}}},1}(t)\propto {\Re} \left\{{\underline{A}}_{s}(t){{{{\rm{e}}}}}^{{{{\rm{j}}}}\Delta \phi (t)}\right\},\,\,\,\Delta \phi (t)={\phi }_{s}(t)-{\phi }_{{{{\rm{LO}}}}}(t).$$

This photocurrent covers a singled-sided bandwidth of 2*B* in the baseband, corresponding to twice the bandwidth of the individual DAC interfaces when considering rectangular Nyquist spectra. The photocurrent represents the output of the proposed PE-AWG (’RF output’), see Inset ③ in Fig. 1a. If needed, an additional electrical amplifier (not shown) can be used for boosting the electrical output power^44–46^.

Equation (1) shows that the generated electrical waveform sensitively depends on the optical phase offset Δ*ϕ* between the LO tone and the optical IQ signal at the inputs of the BPD. To ensure that the PE-AWG output is stable over time and matches the broadband electrical target waveform *s*(*t*), two conditions need to be fulfilled. First, the optical phase offset Δ*ϕ* needs to vanish, and second, the real-valued drive signals *u*_I_(*t*) and *u*_Q_(*t*) of the IQM must be synthesized to satisfy the relations $s(t)=\Re \left\{{\underline{A}}_{s}(t)\right\}$ and ${\widetilde{\underline{A}}}_{s}(f)=0$ for *f* < 0. These two aspects are discussed in more detail in Section S1.3 of Supplement 1.

Note that some parts of our system are similar to those used in previous experiments on optical SSB transmission, e.g., in combination with optical orthogonal-frequency division multiplexing (OFDM) schemes^25,26,47^, and related Kramers-Kronig receiver systems^27–29^. Specifically, many of those schemes use an IQM to generate a broadband optical IQ signal with uncorrelated sidebands that has twice the bandwidth of the underlying DAC channels, along with a strong monochromatic tone, which serves as an LO tone for down-conversion in a square-law detector. Since the signal and the LO tone are already combined, it is impossible to use a BPD at the receiver, and down-conversion has to rely on direct detection instead. If the LO tone is positioned right at the edge of the signal spectrum, direct detection unavoidably leads to a strong spectral overlap between the down-converted signal and unwanted, residual signal-signal mixing products, also known as signal-signal beat interference (SSBI), making such schemes unsuited for high-quality electrical arbitrary waveform generation. These SSBI impairments can be avoided by inserting a guard band between the LO tone and the signal band^25,26^. However, this limits the scheme to the generation of electrical band pass signals with a center frequency that corresponds to at least 1.5 × the bandwidth of the IQ signal. In the next section, we explain how to overcome this problem by combining the broadband optical IQ signal with an independently generated LO tone in an OH while precisely controlling the phase offset Δ*ϕ* between the two. This allows to translate the optical IQ waveform into a well-defined electrical time-domain waveform, that suffers neither from SSBI nor from uncontrolled phase drifts of the LO tone with respect to the signal band.

### Active optical-phase stabilization

The LO tone and the optical carrier are derived from the same laser, and one might hence expect the optical signal to have a fixed phase offset Δ*ϕ* with respect to the LO tone. However, in practice, Δ*ϕ* is usually subject to random drift over time due to vibrations and/or temperature fluctuations in the underlying optical setup, especially if the optical LO tone and/or the optical IQ signal are routed through a sequence of fiber-optic devices such as modulators or amplifiers, as is the case for our proof-of-concept demonstration. An unknown or randomly drifting optical phase offset Δ*ϕ*(*t*) between the LO and the modulated IQ signal renders targeted waveform generation impossible and hence needs to be compensated by an active feedback control. This is achieved by the so-called phase-stabilized coherent combiner (PSCC), that is indicated by a dashed blue box in Fig. 1a and that exploits the two remaining output ports of the 90° OH (‘Q^+^’ and ‘Q^−^’) in combination with a second balanced photodetector (BPD2) for generating the feedback signal. Note that the optical phase offset usually varies rather slowly compared to the electrical target waveform and that the monitoring photodiodes and all associated electronic circuits can be kept at operation bandwidths well below 1 MHz. The PSCC also comprises an electronic controller, consisting e.g., of a DAC, a digital loop filter (LF), an analog-to-digital converter (ADC), as well as an endless phase shifter (PS), which form an electro-optic phase-locked loop (PLL)^48^ together with the 90° OH. These elements are highlighted in turquoise in Fig. 1a, and a more detailed explanation of the underlying concept is given in Section S2.3 of Supplement 1 along with a mathematical description. Note that the phase locking of the LO tone and the optical carrier is still important as the phase control can only compensate for low-speed phase drifts, but not for broadband phase noise that would occur in case of tones derived from two different free-running lasers. We illustrate the importance of the phase control by simulating the impact of non-zero phase offsets Δ*ϕ* on the output waveform, see Fig. 1b. In this investigation, we use a two-level pulse amplitude modulation (PAM2) waveform with a pulse shape having a root-raised-cosine (RRC) spectrum as the target waveform and then subject it to different phase offsets Δ*ϕ*. In case of a vanishing phase offset Δ*ϕ* = 0, we find the expected histogram consisting of two sharp peaks, as indicated in red. In contrast to that, non-vanishing phase offsets Δ*ϕ* ≠ 0, turn the resulting signal into a linear superposition of the electrical target waveform *s*(*t*) and its Hilbert transform ${{{\mathcal{H}}}}\left\{s(t)\right\}$, see Section S1.3 of Supplement 1 for details, and the histogram deteriorates to the point of being unrecognizable as a PAM2 signal. In case of communication signals, this distortion can be undone by an appropriately designed linear adaptive equalizer at the receiver, see Section S3.4 of Supplement 1 for details. For targeted synthesis of waveforms with arbitrary shape, however, precise phase control is imperative.

### Digital synthesis of IQM drive signals

The generation of the target waveform *s*(*t*) at the PE-AWG output poses two requirements on the synthesis of ${\underline{A}}_{s}(t)$ and the associated drive signals *u*_I_(*t*) and *u*_Q_(*t*). First, the generated photocurrent *i*_BPD,1_(*t*) at the high-speed BPD (BPD1) output must correspond to the target waveform *s*(*t*). Assuming that phase control is established, i.e., Δ*ϕ* = 0, the photocurrent is proportional to the real-part of the complex-valued envelope of the optical IQ signal ${\underline{A}}_{s}(t)$ with respect to the LO frequency *f*_1_, see Eq. (1), and requirement 1 hence is

2 $${i}_{{{{\rm{BPD}}}},1}(t)\propto \Re \left\{{\underline{A}}_{s}(t)\right\}=s(t).$$

Second, the complex-valued envelope ${\underline{A}}_{s}(t)$ of the optical IQ signal must be an analytic signal having a single-sided power spectrum, ${\widetilde{\underline{A}}}_{s}(\,\,f)=0$ for *f* < 0. This is important since an IQM with limited bandwidth *B* modulating an optical carrier at frequency *f*_2_ cannot generate spectral components at frequencies below *f*_1_ = *f*_2_ − *B*. The second condition is ensured by choosing the imaginary part as the Hilbert transform of the real part, thus rendering ${\underline{A}}_{s}(t)$ an analytic signal with a single-sided spectrum,

3 $${\widetilde{\underline{A}}}_{s}(\,\,f)=0\,\,{{{\rm{for}}}}\,\,f < 0.$$

Compliance with Eqs. (2) and (3) is achieved by proper digital signal processing, see Fig. 1c for the associated block diagram. As an initial step, a discrete-time version *s*_*n*_ = *s*(*nT*_*s*_) of the broadband target waveform *s*(*t*) (Target wf.) is generated, where *T*_*s*_ denotes the sampling period and *n* is an integer. The spectrum of the target waveform is sketched in Inset Ⓐ of Fig. 1c, where redundant spectral components at negative frequencies are again dashed. Next, the corresponding analytic signal is computed,

4 $${\underline{A}}_{s,n}={s}_{n}+{{{\rm{j}}}}{{{{\mathcal{H}}}}}_{n}\left\{{s}_{n}\right\},$$

with ${{{{\mathcal{H}}}}}_{n}$ representing the discrete-time Hilbert transform. Applying the discrete-time Hilbert transform to a signal corresponds to a convolution of the signal with a discrete-time Hilbert filter

$${h}_{n}=\left\{\begin{array}{ll}0\quad &n\,{{{\rm{even}}}}\\ \frac{2}{\pi n}\quad &n\,{{{\rm{odd}}}}\end{array}\right..$$

The analytic signal is then down-shifted in frequency to be centered at zero frequency, yielding ${\underline{u}}_{n}={\underline{A}}_{s,n}{{{{\rm{e}}}}}^{-{{{\rm{j}}}}2\pi Bn{T}_{s}}$, see Inset Ⓓ of Fig. 1c. Finally, we take the real and imaginary parts ${u}_{{{{\rm{I}}}},n}=\Re \left\{{\underline{u}}_{n}\right\}$ and ${u}_{{{{\rm{Q}}}},n}=\Im \left\{{\underline{u}}_{n}\right\}$ to obtain the digital inputs into the pair of electronic DACs, which generate the two corresponding analog signals *u*_I_(*t*) and *u*_Q_(*t*) for driving the IQM.

### Bandwidth scaling and spectral stitching in the optical domain

Quadrature multiplexing opens a path towards further bandwidth scaling by spectral stitching of tributary signals in the optical domain, exploiting the fact that the bandwidth of advanced photodetectors^9,35,37^ is much higher than that of the latest electronic DACs. The long-term vision of such an ultra-broadband PE-AWG is illustrated in Fig. 1d. The scheme exploits a chip-scale optical frequency-comb generator (FCG)^49^, providing a multitude of phase-locked tones that are individually modulated by an array of *N* = 4 IQ modulators, each driven by an associated pair of electronic DACs. The resulting spectral tributary signals are then spectrally stitched to form a well-defined time-domain broadband optical waveform ${\underline{A}}_{s}(t)$, which is then down-converted to the electrical domain by an ultra-broadband BPD using the remaining comb tone at frequency *f*_0_ as an LO. Importantly, the spectral stitching also relies on precisely controlled optical phases among the various spectral tributaries, which can be accomplished by using essentially the same PSCCs that were described for phase-stabilized coherent down-conversion in Subsection “Active optical-phase stabilization”. For signal stitching, the phase stabilization exploits the interference generated by spectrally overlapping portions of the neighboring signal slices and generates the stitched signal at the OH output labeled with ‘I^+^’ in Fig. 1a, while the ‘I^−^’ output remains unused, see^30^ for details. In the exemplary scheme depicted in Fig. 1d, a tree structure of (*N* − 1) = 3 PSCCs is used for spectrally stitching the *N* = 4 tributaries thus forming a well-defined broadband optical waveform. This concept is illustrated by sketching the spectra of tributary and combined signals at several positions A, B, and C in the setup, see Insets Ⓐ to Ⓒ. After the stitching, the entire broadband optical waveform is down-converted to the electrical domain by mixing with a single optical LO tone in an ultra-broadband balanced photodetector (BPD1) connected to the ‘I^+^’ and ‘I^−^’ outputs of the last PSCC.

While the heterodyne down-conversion of stitched optical waveforms has not yet been shown experimentally, we have already demonstrated generation of optical waveforms with a bandwidth exceeding 300 GHz via spectral stitching, resulting in a signal quality clearly exceeding that of conventional all-electronic waveform-generation schemes^50^ and enabling the first transmission of single-carrier 1 Tbit/s over transatlantic distances^51^. Clearly, for photonic-electronic arbitrary waveform generation, the bandwidth of the photodetectors used to convert the waveform to the electrical domain and the subsequent amplification of the resulting electrical are key. Future PE-AWG schemes may rely on advanced photodetectors with bandwidths of several hundred GHz that have recently been shown^35–37,39^. Similarly, traveling-wave millimeter-wave amplifiers with bandwidths in excess of 300 GHz have been demonstrated^45^, and could be used for boosting the electrical power at the BPD output. Coaxial connectors with a bandwidth of 145 GHz are already commercially available^52^ and further advancements can be expected in the future. The key benefit of the PE-AWG scheme is the fact that it reduces the complexity of the optoelectronic frequency-conversion front-end to a single photodetector and a subsequent amplifier. These rather simple components can be implemented on an ultra-broad-band III-V-based integration platform^53^ that stands out due to speed, but does not offer sufficient yield for implementing more complex mixed-signal circuits. Scaling the bandwidth of the photodetector and the analog amplifier is thus much simpler than scaling the bandwidth of an entire all-electronic AWG, or a high-quality multi-channel millimeter-wave frequency-conversion circuit. Such systems typically consist of large numbers of (Bi)CMOS components as well as additional analog circuits for clock distribution, amplification, or electrical multiplexing, and they are unavoidably much more complex than a single photodetector and/or a single amplifier. This notion is confirmed by the fact that even today, RF amplifiers and balanced photodetectors with 3-dB bandwidth of 100 GHz or more can be bought off the shelf ^46^ at acceptable prices, whereas the most advanced AWGs are still limited to a 3-dB bandwidth of 75 GHz and represent a major investment^54^. In this context, reducing the complexity of the optoelectronic frequency-conversion front-end to a single photodetector and a subsequent amplifier is a key step, since it allows implementing these rather simple components on ultra-broad-band III-V-based integration platforms that stand out due to speed, but do not offer sufficient yield for implementing more complex mixed-signal circuits.

### Experimental implementation

**Fig. 2:**
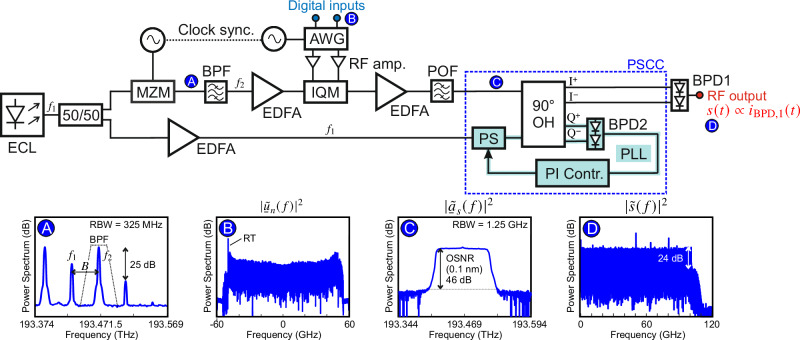
Experimental implementation of the proposed PE-AWG using benchtop-type laboratory equipment along with discrete fiber-optic components. The optical carrier and the LO tone are derived from a common external-cavity laser (ECL), emitting at frequency *f*_1_. In the upper arm of the setup, a frequency shift is applied to the laser tone by using a sine-driven Mach-Zehnder modulator (MZM), that generates a pair of spectral sidebands, and an optical band pass filter (BPF), that suppresses one of the sidebands and the original tone at *f*_1_ such that only the desired optical carrier at *f*_2_ = *f*_1_ + *B* remains. The optical carrier is then modulated in a subsequent IQM, which is driven by a pair of channels of an electronic AWG, time-synchronized with the frequency shifter. The resulting IQ signal is sent through a programmable optical filter (POF) before being superimposed with the LO tone at frequency *f*_1_ in a 90° optical hybrid (OH). Erbium-doped fiber amplifiers (EDFAs) compensate insertion and modulation losses at various positions in the setup. The coherent down-conversion is finally performed by a commercially available 100-GHz balanced photodetector (BPD1), connected to the ‘I^+^’ and ‘I^−^’ outputs of the OH^55^. The insets show power spectra at various positions in the setup and illustrate the filtering of the frequency-shifted tone *f*_2_ Ⓐ, the complex-valued digital signal ${\underline{u}}_{n}$ Ⓑ, the optical spectrum at the input of the down-conversion stage Ⓒ, and the electrical spectrum of the generated target waveform Ⓓ. The PSCC (dashed blue box) contains an electro-optical PL (turquoise) for active stabilization of the optical phase offset Δ*ϕ* between the LO and the IQ signal. To this end, the two remaining outputs of the OH are used to tap monitoring signals to a low-speed BPD, which is connected to either a digital or an analog PI controller generating a feedback signal for driving a piezo-based phase shifter in the LO path. The controller is configured to minimize the $\sin \left(\Delta \phi (t)\right)$ output of the low-speed balanced photodetector (BPD2) and to stabilize the phase difference *Δϕ* of 0, ± 2*π*, ± 4*π*…. With this proof-of-concept implementation of a PE-AWG based on quadrature multiplexing, we generate PAM test signals with up to eight amplitude levels (PAM8) at symbol rates between 100 GBd and 200 GBd.

To demonstrate the viability and performance of the PE-AWG concept illustrated in Fig. 1a, we perform a proof-of-concept system-level experiment that largely relies on conventional discrete fiber-optic components. Figure 2 shows a simplified sketch of the experimental setup - a more detailed description can be found in Section S2.1 of Supplement 1. For evaluating the performance of the system, we generate PAM signals with a RRC-type pulse shape at symbol rates between 100 GBd and 200 GBd. We deliberately chose data signals rather than arbitrary waveforms for our demonstration to simplify evaluation and benchmarking of the results to the existing literature via widely used performance metrics such as the bit-error ratio (BER) and the frequency-dependent signal-to-noise-and-distortion ratio (SNDR). In addition, we performed two-tone measurements to directly quantify linear and nonlinear signal distortions at different frequencies, see Section S4 of Supplement 1 for details. In our PAM signaling experiments, we derive the optical carrier and the LO tone from an external-cavity laser (ECL) with a linewidth below 100 kHz, emitting an optical tone at frequency *f*_1_. The optical power is then split in two identical copies using a 3-dB coupler, and one copy is frequency-shifted to a frequency *f*_2_ = *f*_1_ + *f*_shift_, see upper arm after the splitter in Fig. 2. In our demonstration, the frequency shift is accomplished with a sine-wave-driven MZM operated close to the null point, see Inset Ⓐ of Fig. 2 for the associated output spectrum, and a subsequent optical band pass filter (BPF) having steep transitions is used to suppress the unwanted sideband and the residual tone at frequency *f*_1_. As an alternative, an IQ modulator (IQM) could be used for single-sideband modulation. Note that the frequency shift *f*_shift_ to be applied during digital signal synthesis is dictated by the target waveform and limited by the bandwidth *B* of the electronic DAC, *f*_shift_ ≤ *B*, see Subsection “Photonic-electronic arbitrary-waveform generator (PE-AWG) concept”. Notably, the PE-AWG architecture gives some flexibility in choosing the frequency shift, which allows balancing the signal quality at the waveform generator output over the full bandwidth range by a tailored digital calibration, see Section S2.2.2 of Supplement 1 for details. An erbium-doped fiber amplifier (EDFA) is used in the upper arm to compensate for the modulation loss as well as the optical insertion loss of the MZM and the BPF. The optical carrier at frequency *f*_2_ is then modulated in amplitude and phase using a commercially-available single-polarization LiNbO_3_ IQM with a 3-dB bandwidth of  ~30 GHz. The associated drive signals are generated by a pair of channels of a fully-electronic AWG (Keysight M8194A) based on CMOS-DACs, each with a sampling rate of 200 GSa/s and an analog bandwidth of about 45 GHz^5^. The transmitter DSP used to generate the digital inputs for the electronic AWG follows the principles explained Subsection “Digital synthesis of IQM drive signals” and Fig. 1c above and is discussed in more detail in Section S2.2.1 of Supplement 1. Inset Ⓑ of Fig. 2 shows the power spectrum of the complex-valued digital signal ${\underline{u}}_{n}$ after precompensation of the frequency-dependent attenuation and phase distortions of the hardware components of the PE-AWG along with an additional reference tone (RT) at the lower edge of the spectrum that is required for the optical-phase stabilization, see Section S1.4 of Supplement 1 for details. At the AWG outputs, we use linear RF amplifiers (RF amp.) with 23 dB gain to boost the drive signals prior to feeding them to the modulator. After the IQM, a second EDFA is used to compensate for the insertion and modulation losses. We further employ a programmable optical filter (POF) in the upper fiber arm of our setup to remove out-of-band amplified spontaneous emission (ASE) noise of the EDFAs. We measure an optical signal-to-noise ratio (OSNR) of more than 40 dB (reference bandwidth: 12.5 GHz), see Inset Ⓒ of Fig. 2, which indicates that ASE noise is negligible as an impairment in our PE-AWG. Note that the POF can also be used as an optical pre-equalizer to flatten the spectrum at the high-speed MZM output, see Subsection “Application example: IM/DD fiber transmission experiment” for details. In our experiments, the optical input powers to the OH as well as the LO-to-signal power ratio (LOSPR) are adjusted via the gains of the corresponding EDFA. For coherent down-conversion, we rely on a commercially available 100-GHz indium-phosphide waveguide-integrated balanced photodetector (BPD1)^55^. No broadband RF amplifier has been used at the PE-AWG output in our proof-of-concept experiments. As discussed above, the PSCC (dashed blue box) contains an electro-optical PLL (turquoise), which uses the two remaining outputs of the OH to tap monitoring signals to a low-speed BPD (BPD2). The output of BPD2 is proportional to $\sin (\Delta \phi (t))$, where Δ*ϕ*(*t*) is the time-dependent phase offset between the signal and the LO, see Subsection “Photonic-electronic arbitrary-waveform generator (PE-AWG) concept” above as well as Sections S1.4 and S2.3 of Supplement 1 for details. This output signal is fed to either a digital or an analog proportiona-integral (PI) controller generating a feedback signal that is applied to a piezo-driven fiber-based phase shifter in the path of the LO tone. The controller aims at minimizing the $\sin \left(\Delta \phi (t)\right)$ error signal of the low-speed BPD and is configured to stabilize a phase offset Δ*ϕ* of 0, ± 2*π*, ± 4*π*.... Note that the synthesizer providing the drive signal to the MZM is synchronized with the AWG clock, thereby ensuring perfect synchronization of the frequency offset *f*_shift_ and the modulation sidebands generated by the IQM, which could hardly be compensated by a piezo-based phase shifter with limited range.

### Characterization and performance benchmarking

We finally analyze the performance of the proof-of-concept PE-AWG implementation in an electrical back-to-back configuration by connecting the high-speed output directly to a 100-GHz real-time oscilloscope with a sampling rate of 256 GSa/s and by offline-processing and -analysis of the recorded waveforms, see Section S2.2.3 of Supplement 1 for details. The digital receive signal features an original length of 2 million samples, corresponding to approximately 20*μ*s. It is re-sampled to 2 samples per symbol and a feed-forward timing recovery^56^ is used to compensate the deviations between the receiver and the transmitter clock. Next, a receive filter with an root-raised-cosine (RRC) spectrum is applied as a matched filter, and the signal is down-sampled to one sample per symbol. As a last step, an optional symbol-spaced adaptive linear equalizer with *L* = 100 coefficients may be used to compensate residual inter-symbol interference (ISI) that has not yet been eliminated by the predistortion of the drive signals. In this case, the equalizer coefficients are updated blindly according to the Sato algorithm^57^ in a first processing stage, which is followed by a second stage relying on the least-mean-squares (LMS) algorithm for a decision-directed update of the coefficients^58^. Unless specified differently in the following, all measurements are performed with the active phase stabilization being turned on. As a metric for the quality of the generated signals, we use the signal-to-noise-and-distortion ratio of PAM signals SNDR_PAM_ as defined in Section S2.2.4 of Supplement 1. Note that the use of timing recovery, matched filtering, and optional adaptive equalization prior to extracting the SNDR_PAM_ as a signal-quality metric allows to compensate for a series of distortions that are largely inherent to any characterization setup and that are not directly related to the signal-generation technique. The approach is therefore commonly used in the literature to quantify the performance of high-speed DACs^1,2,5,21^ allowing for a direct comparison of the SNDR_PAM_ values obtained in our experiments to those demonstrated in earlier publications, see Subsection “Symbol rate sweep”.

### Signal quality and electrical output power

In a first step of our analysis, we optimize the signal quality, quantified by the SNDR_PAM_, and investigate its interdependence with the electrical power at the PE-AWG high-speed output. Specifically, for heterodyne down-conversion in a BPD with limited optical input power, the electrical output power of the RF beat signal is maximized if the optical IQ signal and the optical LO incident to the BPD have equal power, ${P}_{s}={P}_{{{{\rm{LO}}}}}={P}_{\max }/2$, where ${P}_{\max }$ denotes the maximum input power tolerated by each of the two pn-junctions of BPD1, and where *P*_*s*_ and *P*_LO_ refer to the signal and the LO power found at each of the outputs of the OH. In real devices, however, imperfections of the BPD such as a limited common-mode rejection ratio (CMRR) lead to an inherent trade-off between performance and output power by causing SSBI^28^. As a consequence, the best quality of the generated electrical signal is commonly obtained for an LOSPR (LO-to-signal power ratio, as introduced in Subsection “Experimental implementation”) greater than 1, which does not provide the highest possible electrical output power. The down-converted signal is further deteriorated by a residual delay (skew) between the two BPD arms, which may result from lengths variations of the OH outputs and/or the BPD fiber pigtails. Such a skew results in additional SSBI and in an imperfect temporal coincidence of the two signal-LO beating terms at the BPD output, and both distortions get stronger with increasing frequency. The 100-GHz BPD from Fraunhofer HHI employed herein features a CMMR of ≥30 dB from 0 to 70 GHz and of ≥25 dB between 70 and 100 GHz. The specified maximum input power ${P}_{\max }$ amounts to 14 dBm for each detector of the balanced pair, but we observe slight bandwidth reductions and nonlinear behavior already at powers of around 9 dBm and hence refrain from exceeding this level.

**Fig. 3:**
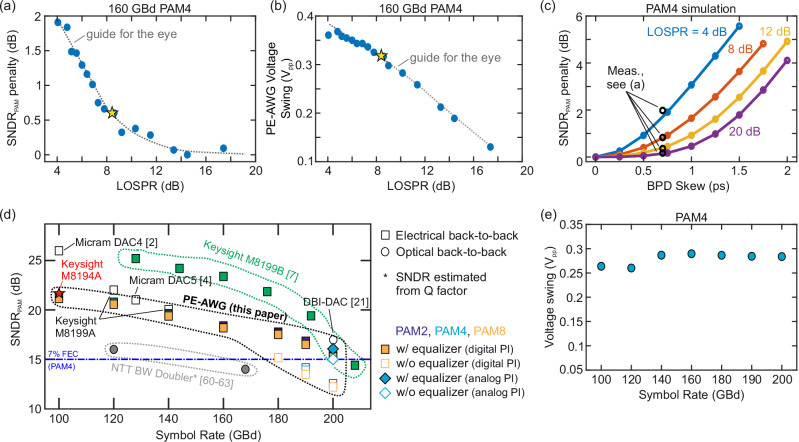
Characterization of the PE-AWG in an electrical back-to-back system, where the PE-AWG output is directly connected to a 100-GHz real-time oscilloscope. As a performance metric, we use the SNDR estimated from received PAM symbols (SNDR_PAM_). **a** SNDR_PAM_ penalty as a function of the LO-to-signal power ratio (LOSPR). The penalty is measured with respect to the optimum LOSPR of 14.5 dB and initially decreases with increasing LOSPR, because the residual signal-signal beat interference (SSBI) decreases. **b** Peak-to-peak voltage swing measured at the PE-AWG output as a function of the LOSPR. Since the electrical output power grows with decreasing LOSPR, a trade-off between output voltage swing and signal quality needs to be made. The yellow stars in Subfigures (**a**) and (**b**) correspond to the levels used in the subsequent measurements shown in Subfigures (**d**) and (**e**). **c** Simulated SNDR_PAM_ penalty for BPD skews between 0 and 2 ps and for different LOSPR levels as indicated by the different colors. We find a SNDR_PAM_ penalty of 0.9 dB for a LOSPR of 8 dB and a skew of 0.7 ps as specified for the BPD used in our experiments. We hence conclude that the skew results mainly from a path-length mismatch between the two fiber pigtails connected to the BPD, such that future integration of the PE-AWG can improve the SNDR_PAM_. **d** SNDR_PAM_ levels achieved for generating PAM waveforms at various symbol rates with the PE-AWG and with several other waveform generators, comprising both commercially available AWGs^2,4,7^ as well as other research-type waveform generators^21,60–63^. Square markers refer to results obtained in electrical back-to-back measurements, whereas circular markers represent optical back-to-back experiments. In case of the PE-AWG, we compare the SNDR_PAM_ for the case without and with an adaptive equalizer (*L* = 100 taps) used to remove residual inter-symbol interference, see the associated square markers with white filling and colored filling, respectively. The PE-AWG relies on Keysight’s AWG model M8194A (red star) and offers a signal quality that is on par with that offered by some commercially available waveform generators such as the Keysight M8199A or the Micram DAC5, while featuring a much higher usable bandwidth than these devices. At symbol rates beyond 140 GBd, the PE-AWG is only outperformed by Keysight’s most recent AWG model M8199B (green markers), which was not available as a signal source for our experiments at the time they were conducted. **e** Average voltage swing observed at the PE-AWG output for RRC pulses with a spectral roll-off of *ρ* = 0.05. We achieve decent voltage swings between 250 and 300 mV_pp_ irrespective of the symbol rate.

To find a good compromise between the power and the quality of the generated target waveform, we sweep the LOSPR during the generation of a 160 GBd PAM4 signal while keeping the total receive power *P*_rx_ = *P*_*s*_ + *P*_LO_ at a constant level of 8 dBm and measuring the SNDR_PAM_. The measured SNDR_PAM_ penalty and the peak-to-peak voltage swing measured at the PE-AWG output are depicted in Fig. 3a and b, respectively, as a function of the LOSPR. For calculating the SNDR_PAM_ penalty, the optimum performance at an LOSPR of 14.5 dB is taken as a reference. We observe a negligible penalty at higher LOSPR along with an increase up to 2 dB at an LOSPR of 4 dB. On the other hand, high output swings of ≥350 mV_pp_ require low LOSPR, whereas the swing is reduced to slightly above 100 mV_pp_ at high LOSPR levels, see Fig. 3b. For the further experiments discussed in this paper, we therefore decided to operate the system with an LOSPR of 8.3 dB as a compromise of output voltage swing and signal quality - the related SNDR_PAM_ penalty and voltage swing of ~300 mV_pp_ are highlighted by yellow stars in Fig. 3a and b. It should be noted that the SSBI in our BPD is mainly caused by the BPD skew and not by an insufficient CMRR. This fact becomes apparent through a simulation of the PE-AWG, investigating the effect of the BPD skew on the SNDR_PAM_ for PAM4 signals, see Fig. 3c. For the sake of simplicity, we neglect bandwidth limitations and ISI in this simulation. We further fix the optical signal-to-noise ratio (OSNR) to 35 dB and assume a frequency-independent CMRR of 30 dB to approximate the real conditions in our experiment. The simulation is repeated for various LOSPRs from 4 dB to 20 dB as indicated by the different colors in Fig. 3c. We find very good agreement between the simulated SNDR_PAM_ penalty (colored curves) and the SNDR_PAM_ penalty measured in our back-to-back measurements (black circles) for a BPD skew of 0.7 ps, which coincides with the value measured by the BPD manufacturer. This skew corresponds to a path length mismatch of  ~150*μ*m within the optical fibers. Looking closer at the case of LOSPR = 8 dB, which is similar to our experimental conditions, we see that the PE-AWG performance could be further improved by reducing the path length difference of the two BPD arms. This will be straightforward to achieve in a future integrated version as sketched in Fig. 1a.

### Symbol rate sweep

In a next step of our analysis, we investigate the deterioration of the SNDR_PAM_ with increasing symbol rate. To this end, we use PAM signals with up to 8 levels at various symbol rates from 100 GBd to 200 GBd and measure the corresponding SNDR_PAM_, see colored markers in Fig. 3d, where each color represents a specific number of PAM levels. For all signals, we use RRC-type pulses with a roll-off factor of *ρ* = 0.05. We use an adaptive equalizer with *L* = 100 real-valued coefficients – the corresponding data points are indicated by square markers with colored filling. Note that some markers fall on top of each other such that only the orange PAM8 marker is visible. For symbol rates of 180 GBd and higher, we additionally investigate the signal quality obtained without equalizer as a reference, indicated by empty square markers. At a symbol rate of 200 GBd, we further plot the SNDR obtained when using an analog PI controller in the active phase stabilization circuit instead of a digital one, see Subsection “Active optical-phase stabilization” for details. The corresponding SNDR_PAM_ levels with and without equalizer are shown as empty and filled diamonds in Fig. 3d. For PAM4, we measure a SNDR_PAM_ of 21.2 dB at 100 GBd and up to 16.1 dB at 200 GBd when using adaptive equalization. Note that a better SNDR is achieved with the analog PI controller because of a significantly reduced latency compared with the digital implementation, which leads to less phase-induced distortions and to more stable SNDR performance over time, thereby also allowing more optimal predistortion filters to be derived. Details can be found in Section S2.3.1 of Supplement 1. The SNDR levels at 100 GBd and 200 GBd correspond to BER levels of 1.3 × 10^−4^ and 2.2 × 10^−3^, respectively. The BER levels were obtained by demodulating the received signals and by counting the actual bit errors in the framework of our offline DSP used for evaluation of the PE-AWG performance, see Section S3.3.1 of Supplement 1 for details. Note that both BER values are well below the BER threshold for forward error correction (FEC) with a hard-decision decoder and 7% coding overhead (BER_7%FEC_ = 4.5 × 10^−3^,^59^). We observe slight SNDR_PAM_ variations when changing the number of amplitude levels, which we attribute to different peak-to-average power ratios (PAPR) of the I and the Q signals that are associated with the various modulation formats. The penalty when omitting the adaptive equalizer lies between 2 and 3 dB when using a digital PI controller and is attributed to two effects: First, the low-pass characteristics of the AWG used to generate the IQM drive signals were only partly compensated by the underlying linear predistortion scheme. Notably, this scheme was based on a minimum-mean-square error (MMSE) filter and does hence not fully compensate the low-pass characteristics of the AWG. Instead, the MMSE filter finds an optimum trade-off between distortions caused by bandwidth limitations along the analog signal path and distortions caused by the fact that pre-amplification of high-frequency signal components increases the peak-to-average power ratio (PAPR), thereby effectively limiting the SNR of the generated analog signal. The uncompensated part of the AWG bandwidth limitations is thus taken care of by the adaptive equalizer, see Section S2.2.1 of Supplement 1 for details. Second, slight imperfections of the active phase stabilization might lead to an imperfect calibration of the PE-AWG and thus degrade the SNDR_PAM_, but can still be compensated by the adaptive equalizer as discussed in Section S3.4.3 of Supplement 1. For our purely analog phase-control implementation, the SNDR_PAM_ decreases by only 1 dB when omitting the adaptive equalizer, which indicates a stable performance of the PE-AWG.

To benchmark the performance of the proposed PE-AWG, Fig. 3d also includes the SNDR_PAM_ levels obtained with various commercially available DACs and AWGs and with other research-type waveform generators reported in the literature. If no reference is provided for the respective data point in Fig. 3d, the SNDR_PAM_ has been measured in our own laboratory. Square markers refer to results obtained in electrical back-to-back measurements, whereas circular markers represent optical back-to-back experiments, for which the performance of the underlying electrical waveform generator can typically be expected to be 1-2 dB higher^4^. Note that for some publications, no SNDR_PAM_ data have been available. In these cases, we estimated the SNDR_PAM_ level from the Q factor – the associated label is indicated with a star (*). Our proof-of-concept PE-AWG relies on Keysight’s CMOS-based AWG model M8194A^5^, that can generate data signals with symbol rates of up to 100 GBd at acceptable performance. The corresponding SNDR level is highlighted as a red star on the very left of Fig. 3d. This underlying electronic signal source limits our PE-AWG to the generation of PAM signals with symbol rates of up to 200 GBd. Overall, we find that the signal quality (SNDR_PAM_) of our PE-AWG can well compete with that of several commercially available electronic waveform generators such as the underlying CMOS-based Keysight AWG model M8194A itself ^5^, the Micram DAC5^4^, or the Keysight M8199A, while offering symbol rates that are clearly above the 140 GBd achieved by the Keysight M8199A. At symbol rates beyond 140 GBd, the performance of the PE-AWG is only overcome by Keysight’s most recent AWG model M8199B, which is based on latest advancements in SiGe technology and which was not available as a signal source for our measurements at the time they were conducted. This AWG model has already been used for generation of symbol rates up to 260 GBd, but its nominal 3-dB bandwidth of 75 GHz^54^ is associated with a sharp drop of the signal quality for symbol rates beyond 200 GBd. Specifically, an SNDR_PAM_ of only ~5 dB was reported at 260 GBd. Details can be found in see Section S3.3 of Supplement 1, where we also indicate the SNDR_PAM_ of state-of-the-art AWGs for symbol rates higher than 200 GBd that could be captured by our 100 GHz photodetector and real-time oscilloscope. Still, although the first-generation PE-AWG reported herein relies on multiplexing of two bandwidth-limited CMOS DAC channels inferior to those of the Keysight M8199B and even though our system is built from discrete components, the resulting signal quality at symbol rates of 180 GBd and above comes close to that of the Keysight M8199B. Moreover, the PE-AWG remains superior to most other bandwidth-enhancing concepts reported in the literature such as the electrical bandwidth-doubling scheme reported in^60–63^ or active electrical time-division multiplexing as described in ref. ^34^. The SNDR_PAM_ measured for a so-called digital-bandwidth-interleaving DAC (DBI-DAC)^21,22,64^ is slightly higher (~1.5 dB) than that of our PE-AWG at 200 GBd. Note, however, that the SNDR_PAM_ of the PE-AWG measured at 200 GBd is an underestimation of the true SNDR_PAM_, as it is already deteriorated by the roll-off of our 100-GHz real-time oscilloscope in contrast to the 113-GHz oscilloscope used in^21,22,64^ and ^8^. Note also that Fig. 3d may give the impression that the frequency-dependent SNDR decay observed for our PE-AWG system has a somewhat more “flat” behavior towards high symbol rates than competing devices like the M8199B, and might have the potential to outperform their SNDR performance for symbol rates beyond 200 GBd. However, this claim could not be substantiated experimentally due to the sharp software-based bandwidth limitation of our oscilloscope to 100 GHz. In Fig. 3e, we display the voltage swing at the PE-AWG output as a function of symbol rate. We observe that voltage swings of 250 mV_pp_ to 300 mV_pp_ can be achieved irrespective of the symbol rate, which is only slightly lower than the 400 mV_pp_ to 500 mV_pp_ of single-ended output voltage that are typically obtained from high-speed CMOS DACs^65^. More details on the comparison of our PE-AWG to the underlying electronic AWG and other waveform generator schemes can be found in Section S3.2 and Section S3.3 of Supplement 1.

To further quantify the quality of the generated waveforms and to identify sources of signal impairments, we additionally investigate the frequency-dependent SNDR choosing a 190 GBd PAM4 waveform, see Section S3.1 of Supplement 1 for details. We deliberately stayed slightly below the maximum achievable symbol rate of 200 GBd, which would lead to significant distortions arising from the bandwidth limitations of the 100 GHz real-time oscilloscope and which might hence not lead to a reliable SNDR measurement of the PE-AWG. In these experiments, we use and compare two different predistortion schemes to compensate for bandwidth limitations and other non-idealities of the underlying system components. Specifically, we use again the purely linear predistortion scheme based on an MMSE filter, which corresponds to a straightforward efficient-to-implement approach and we compare it to an idealized nonlinear predistortion scheme based on a pattern-dependent look-up table, which provides an upper bound for the performance achievable with more advanced nonlinear predistortion schemes, e.g, based on Volterra-type pre-equalizers^66^ or neural networks^67^. We find that the linear predistortion is already quite effective, leading to an average SNDR of approx. 17 dB for the frequency range between 0 and 95 GHz, which would correspond to an effective number of bits (ENOB) of 3.7, see Section S3.2.1 of Supplement 1 for details. The idealized nonlinear predistortion increased the average SNDR to approximately 20 dB, corresponding to an ENOB of of approximately 4.2. In additional experiments, we analyze the SNDR and related performance metrics as a function of the IQM drive power when generating two target tones. These experiments give a more direct insight into the presence and strength of nonlinear distortions, see Section S4 of Supplement 1 for details.

### Application example: IM/DD fiber transmission experiment

**Fig. 4:**
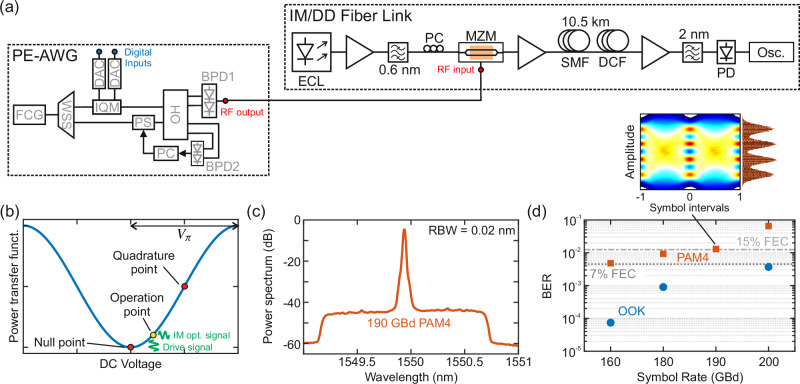
IM/DD fiber-optic transmission experiment using the PE-AWG as an electrical signal source. **a** Experimental setup. The broadband electrical waveforms generated with the PE-AWG are used to drive an optically packaged thin-film LiNbO_3_ MZM^31^ for intensity modulation of an optical carrier. The optical waveform is transmitted over 10.5-km of standard single-mode fiber (SSMF) followed by a dispersion-compensating fiber (DCF) of appropriate length. A 100-GHz PD is used to detect the received signal, and a 100-GHz oscilloscope records the resulting electrical waveform. EDFAs are used to compensate for optical losses in the setup and to adjust the input power at the receiver. **b** Illustration of the transfer function of the MZM and of the used operation point (yellow dot). We operate close to the minimum-transmission point (null point) to reduce the carrier-to-sideband ratio of the resulting optical data signal. **c** Double-sided optical spectrum measured after the DCF for a 190-GBd PAM4 signal with an overall optical bandwidth that essentially corresponds to the full symbol rate. The slight asymmetry of the spectrum is caused by the inclined gain profile of the last EDFA in the fiber link. **d** BER vs. symbol rate for the IM/DD experiment. For OOK, we measure BER levels below the 7% hard-decision FEC limit even at a symbol rate of 200 GBd. For PAM4, the BER for 190 GBd is still below the BER threshold for soft-decision FEC with a 15% overhead. The inset shows an exemplary eye diagram obtained for a 190-GBd PAM4 waveform, obtained by 20-fold up-sampling of the demodulated signal and by applying an interpolation filter with RC-type spectrum. The inset further shows the histogram evaluated at the center of the eye.

To demonstrate the viability of the PE-AWG approach for high-speed communications, we use our demonstrator as an electrical signal source in an optical transmission experiment relying on intensity modulation and direct detection (IM/DD). In a first step, we generate broadband electrical PAM2 and PAM4 waveforms, which lead to optical OOK and PAM4 signals, respectively. The electrical waveforms are fed to an optically packaged thin-film LiNbO_3_ MZM with a nominal bandwidth of 100 GHz and a *π*-voltage of *U*_*π*_ = 2.4 V^31^, see Fig. 4a. The MZM modulates the intensity of an optical carrier at 1550 nm, which is supplied by an ECL. Due to the relatively small voltage swings of approximately 300 to 350 mV_pp_ obtained at the output of the PE-AWG, see Fig. 3e, the modulation depth is relatively small and we boosted the optical carrier to a power of 26 dBm to ensure sufficient power in the optical sidebands after the MZM. Note that we used a slightly lower LOSPR compared to the back-to-back experiments, which increases the voltage swing and thus the modulation depth at the expense of additional SSBI. We account for the roll-off of the MZM’s frequency response by using the POF in the PE-AWG, see Fig. 2, as an optical pre-equalizer. An additional EDFA is used to amplify the optical signal after the MZM before it is launched into a 10.5 km-long standard single-mode fiber (SMF) with an initial optical power of 10 dBm. The accumulated chromatic dispersion of 173 ps/nm is compensated by a dispersion-compensating fiber (DCF) of appropriate length. Finally, the intensity-modulated signal is detected with a 100-GHz photodiode (PD) and captured by a 100-GHz real-time oscilloscope (Osc.). Since we did not have a broadband transimpedance amplifier at the receiver, we use a third EDFA at the input of the receiver to boost the incoming optical power to 9 dBm, which ensures proper reception without any impairment by oscilloscope quantization noise. DSP is performed offline, including resampling, retiming, and adaptive equalization with a linear time-domain filter whose coefficients are updated with the decision-directed least-mean-squares (LMS) algorithm. The equalizer compensates bandwidth limitations of the modulator and the photodiode, as well as weak distortions induced by a residual phase offset Δ*ϕ*(*t*), see Section S3.4 of Supplement 1 for details. It can further partially undo intersymbol interference that is caused by residual CD, as long as the dispersion-induced spectral nulls are outside the spectral range covered by the received signal. In our experiment, this is ensured by compensating most of the dispersion via an appropriate DCF, such that only residual dispersion is effective at the receiver. Due to the strong optical carrier associated with intensity modulation around the quadrature point of the MZM, the rather small modulation depth, and the optical input power limit of the receiver photodiode, the voltage swing observed after direct detection is rather small. This problem can be mitigated by shifting the MZM operation point towards the minimum-transmission point (null point), which reduces the optical carrier-to-sideband power ratio of the optical data signal. We identify the point with a carrier suppression of 13 dB as an optimum, illustrated by a yellow dot in Fig. 4b. Figure 4c displays the optical double-sideband spectrum of a 190-GBd PAM4 signal at the output of the DCF. The overall optical bandwidth essentially corresponds to the full symbol rate and amounts to approximately 200 GHz. In our experiments, we sweep the symbol rate and record the BER, see Fig. 4d. For 200 GBd OOK, the BER is below the threshold for hard-decision FEC with 7% overhead^59^, whereas the BER for 190 GBd PAM4 is still low enough for soft-decision FEC with 15% overhead^59^. Note that the 200-GBd waveforms with RRC pulse shaping (roll-off-factor *ρ* = 0.05) cover an electrical bandwidth of approximately 105 GHz after down-conversion and are hence deteriorated by the limited bandwidth of our oscilloscope, which features a sharp drop of the frequency response at 100 GHz. The inset of Fig. 4d shows an exemplary eye diagram obtained for a 190-GBd PAM4 waveform detected by the IM/DD receiver along with the associated histogram captured at the center of the eye. The eye diagram was digitally generated by up-sampling the sequence of receive symbols 20 times and by applying a RC-type interpolation filter with a roll-off of 0.1. We observe most bit errors between the two lowest levels and attribute this to a slight compression of the histogram peaks towards the left-hand side caused by the nonlinearity of the MZM transfer function at operating points close to the null point. In future experiments, a nonlinear predistortion resulting in non-equidistant spacing of the drive amplitude levels might help improving the performance. Clearly, the achievable performance gain will eventually be limited by the ENOB of the PE-AWG, which currently amounts to approximately 3.7. Note that this number might be increased in future PE-AWG implementations, see Section S5 of Supplement 1 for details. We also measure the normalized generalized mutual information (NGMI) of the transmitted PAM4 signals, which amount to 0.9691, 0.9396, 0.9225, and 0.6989 at symbol rates of 160, 180, 190, and 200 GBd, respectively.

## Discussion

### Further improvements, integration, and use cases of PE-AWG systems

While our proof-of-concept experiment shows the potential of the proposed PE-AWG, the current implementation clearly leaves room for further improving the quality of the generated waveforms, see Section S5 of Supplement 1 for a more detailed discussion. Specifically, we believe that replacing the currently used electronic AWG (Keysight M8194A) by another device with a lower frequency-dependent roll-off and/or ENOB (e.g., the Keysight M8199A or M8199B) would translate into significant performance improvements of the overall PE-AWG system. This notion is based on our analysis of noise and distortions of the PE-AWG system in Section S3.2 of Supplement 1, where we identify the contributions of the electrical AWG as one of the dominant impairments, see Fig. S5d of Supplement 1. Moreover, the current system suffers from strong RF amplifier noise and distortions, originating from the IQM drive amplifiers, see yellow trace in Fig. S5d as well as Section S3.2 of Supplement 1. With the new M8199B offering nearly twice the frequency-dependent output-voltage swing than its older M8194A counterpart at 100 GBd, these IQM drive amplifiers could be significantly tuned down or even omitted completely, which would lead to another significant reduction in noise. The same principle applies to the IQM - the currently used devices have a bandwidth of 30 GHz and could be replaced by more broadband ones^31,32^, which might even be more efficient^31,68,69^ thus obviating the need for drive amplifiers altogether.

Further gains in performance, robustness and scalability can be achieved by consequent use of advanced PICs and photonic-electronic co-integration. For instance, integration may reduce the physical distance between the DAC and the IQM and thus bring down the associated RF path loss, such that driver amplifiers might become obsolete. With respect to coherent down-conversion, the length mismatch between the two paths connecting the OH to the high-speed BPD inputs may be reduced by photonic integration, which would effectively decrease the level of SSBI impairments at lower LOSPR levels, see Fig. 3c and the associated discussion above. As a result, the LOSPR could be reduced, leading to down-converted waveforms with higher electrical power compared to our proof-of-concept demonstration. In the context of integration, the key challenge might be to accommodate all functional components shown in Fig. 1a into a chip-scale system. Due to a wide range of required functionalities, we believe that a chiplet-based integration concept, which can leverage integration platforms with strongly complementary strengths, might have clear advantages over a monolithic integration approach, where all components rely on a single material system. Specifically, frequency-agile narrow-linewidth CW lasers can be implemented by combining III-V-based gain elements with tunable feedback circuits on the silicon photonic^40^ or the silicon nitride platform^70^. High-performance IQMs as well as phase and frequency shifters can be efficiently integrated using for example thin-film lithium niobate^71,72^ or thin-film lithium tantalate^73^. Another approach might leverage a hybrid combination of silicon photonic waveguides and organic electro-optic materials, leading to so-called silicon-organic hybrid (SOH) devices^74^. Passive components such as optical hybrids are available on many platforms and can be co-integrated either with the electro-optic modulators or with the subsequent photodetectors. The ultra-broadband BPD that is used for generating the electrical output signal is another key component. Conventionally, BPDs rely on dedicated III-V structures that offer vast design flexibility^75^. Alternatively, ultra-fast germanium photodiodes that are integrated into silicon photonic circuitry^35^ or plasmonic internal photoemission detectors^39^ have been demonstrated to reach bandwidth well above 100 GHz. A key challenge in complex photonic integrated circuits is related to overcoming optical loss by amplification. The conventional approach is to use semiconductor optical amplifiers, which are compact and highly efficient. These devices, however, might introduce signal distortions due to limited excited-state lifetime and typically feature higher noise figures than fiber-based amplifiers. More recently, so called erbium-doped waveguide amplifiers (EDWAs) relying on ultra-low-loss silicon-nitride (Si_3_N_4_) waveguides have been demonstrated to overcome these limitations, while offering compact footprint and temperature insensitivity^76,77^. All these components may come on dedicated chiplets, which need to be optically connected on a package level, often requiring costly and sensitive active alignment techniques for system assembly. These challenges can be overcome by using 3D-printing techniques based on multi-photon polymerization to structure chip-to-chip waveguides, so called photonic wire bonds^41,78^, or facet-attached micro-lenses (FaML)^42,79^. Note that an integrated PE-AWG will very likely still require an active optical-phase stabilization to guarantee high-quality generation of target waveforms - even though the speed and the magnitude of the unwanted phase drifts is much smaller than for a fiber-optic system implementation such as the one shown in our experiment. However, with smaller overall phase drifts, the phase shifter can be greatly simplified, obviating the need for a large control range or an endless phase shifter.

Another important aspect of an integrated system is related to the impact of optical reflections and backscattering, which might occur on much shorter distances and with potentially higher magnitudes than in fiber-based systems. In this context, the most crucial part of the system illustrated in Fig. 1a is very likely the LO path, i.e., the lower path leading to the feedback-controlled phase shifter. This path, however, is reasonably simple and might be optimized to exhibit sufficiently low back-reflections not to distort the CW laser. Note that we have previously connected DFB lasers to silicon photonic circuits via photonic wire bonds or FaML without any isolators in between, without observing any distortions from parasitic back-reflections^42,78,80^. In contrast to the LO path, the signal path comprises a frequency shifter, which, due to its traveling-wave configuration, is only efficient in one direction and hence acts at least partially as an on-chip isolator. This path might require additional amplification, e.g., after the IQM, and here care must be taken to keep parasitic back-reflections from the subsequent OH and BPD sufficiently low. Interestingly, our current experiments already give some insight into the performance gains that could be expected from an integrated PE-AWG system. As an example, Fig. 3c shows the impact of the BPD skew on the SNDR_PAM_, where the solid lines correspond to simulations based on a simple theoretical model, the validity of which was confirmed by the measurements shown in Fig. 3a, see Subsection “Characterization and performance benchmarking” of Section “Results” for more details. As explained above, for an integrated PE-AWG system, the lengths of the BPD detection paths can be carefully matched, leading to negligible skew. Following the predictions of our experimentally confirmed model in Fig. 3c, it should then be possible to reduce the LOSPR to 4 dB or less without incurring any penalties on the signal quality. This should open design freedom for system implementations without optical amplification in the LO path. These results are fairly encouraging for future system implementations, even though a demonstration of an integrated PE-AWG system as illustrated in Fig. 1 certainly remains a breakthrough in its own right.

The main application potential of our PE-AWG lies in precise electrical waveform generation with unprecedented bandwidth, as e.g., needed for advanced test & measurement equipment. Such equipment is important in many areas of science an engineering, not only for time-domain testing of high-speed electronic and millimeter-wave components or systems such as ultra-broadband wireless transmitters^81^, but also for high-speed electro-optical devices such as modulators that are used in optical transceivers. In fact, the communications industry currently faces the situation that even the most advanced electrical AWGs cannot keep up with real-time oscilloscopes in terms of bandwidth - a prominent problem that should not be underestimated. More specifically, Keysight’s most recent AWG model M8199B features a 3-dB bandwidth of 75 GHz^54^, whereas the Keysight UXR oscilloscope series offers a 3-dB bandwidth up to 113 GHz^82^. The lack of precise and broadband signal sources is a fundamental problem for the industry - high quality waveform generation with the Keysight M8199B is limited to symbol rates of 200 GBd, see Fig. S6 in Supplement 1, while commercial optical transceivers with these symbol rates have already publicly been announced^14^ and systems supporting 240-280 GBd are expected to enter the market soon^83^. In this context optoelectronic signal processing as proposed in our work can be an attractive solution, especially in light of the problem that the test & measurement industry cannot leverage advanced CMOS technology nodes for simple commercial considerations: The mask design for a single 3-nm CMOS chip requires typical R&D investments of the order of 40 Mio. USD^84^, which can be justified for large-volume markets such as optical transceivers, but which are unrealistic for any medium- or low-volume application such as test & measurement equipment. This notion is supported by the fact that, following the introduction of Keysight M8194A based on 16-nm CMOS^5^, all further AWG generations have relied on 55-nm BiCMOS circuits while leveraging complex analog front-ends for combining two underlying DAC channels^8^. This makes further progress not only technically challenging, but probably also expensive. We believe that the proposed PE-AWG concept, possibly in combination with spectral stitching in the optical domain as illustrated in Fig. 1d, can exploit the massive bandwidth potential of photonic circuits and may pave a path towards efficient bandwidth scaling of waveform generators. As discussed above, this approach mainly requires scaling the bandwidth of the balanced photodetector and the subsequent electrical millimeter-wave amplifier instead of scaling the bandwidth of the underlying DAC or a complex electrical front-end. Note also that our scheme offers the conceptual advantage of making long lossy and expensive RF cables obsolete: Ultra-broadband waveforms can be easily transported in low-loss optical fibers without impairing the PE-AWG bandwidth, while precise, phase-preserving conversion from the optical to the electrical domain can be done at the very end, close to the point where the ultra-broadband electrical waveforms are actually needed. As an example, when it comes to testing chip-scale components with RF probes, the PE-AWG scheme would allow for implementing an optoelectronic probe head that performs optical-to-electrical conversion just at the interface to the RF probe. Such schemes would only require local probe tips and chip-chip coupling structures that can be obtained by high-resolution 3D printing techniques^85^.

## Summary

We demonstrate an ultra-broadband photonic-electronic arbitrary-waveform generator (PE-AWG) that relies on quadrature multiplexing of optical waveforms and subsequent phase-stabilized down-conversion in a high-bandwidth balanced photodetector (BPD). The proposed PE-AWG concept allows synthesizing arbitrary electrical waveforms with twice the bandwidth of the electronic digital-to-analog converters (DACs) that are used to generate the drive signals of the underlying optical IQ modulator (IQM). The active phase stabilization is instrumental for targeted signal synthesis and relies on a phase-locked loop (PLL) consisting of a low-bandwidth BPD, a PI controller, and a piezo-driven fiber-optic phase shifter. We provide a detailed characterization of the PE-AWG with respect to electrical output power, signal quality, frequency-dependent distortions, and the precision of the phase stabilization. Notably, the signal quality obtained in our proof-of-concept experiments can already compete with that of commercially available AWG systems. To demonstrate the viability of the scheme, we finally use our PE-AWG to drive a high-speed Mach-Zehnder modulator (MZM) for generating optical on-off-keying (OOK) and PAM4 signals with symbol rates of up to 200 GBd and 190 GBd, respectively. To the best of our knowledge, the underlying 100 GHz-wide waveform represents the most broadband electrical data waveform that was so far generated by a photonic-electronic system. In combination with recently demonstrated phase-stabilized spectral stitching of optical waveforms^30^, the scheme paves a path towards efficient bandwidth scaling of waveform generators with linearly increasing hardware effort exploiting massive parallelization of low-speed DAC arrays and leveraging the tremendous progress of broadband electro-optic devices such as modulators and ultra-high-speed photodetectors.

### Reporting summary

Further information on research design is available in the Nature Portfolio Reporting Summary linked to this article.

## Supplementary information

Supplementary Information

Reporting Summary

Transparent Peer Review file

## Data Availability

Data used to produce the plots within this paper is available at https://zenodo.org/records/15214548. All other data used in this study are available from the corresponding authors upon request.

## Code availability

The code used to produce the plots within this paper has been deposited in Zenodo (https://zenodo.org/records/15214548). Further code is available from the authors upon request.
