# Supplementary material for: Photonic-electronic arbitrary-waveform generation using quadrature multiplexing and active optical-phase stabilization
Source: Nat Commun. 2025 Sep 18;16:8318. doi: 10.1038/s41467-025-61564-w (PMC12446453; doi:10.1038/s41467-025-61564-w)
Supplement: Supplementary file 1 — Supplementary Information [file 41467_2025_61564_MOESM1_ESM.pdf]

Photonic-Electronic Arbitrary-Waveform Generation Using Quadrature Multiplexing and Active Optical-Phase Stabilization (Supplementary Information)

Christoph Füllner[1*], Alban Sherifaj[1], Thomas Henauer[2], Dengyang Fang[1], Daniel Drayss[1], Lennart Schmitz[1], Tobias Harter[1], Tilahun Z. Gutema[1], Thomas Zwick[2], Wolfgang Freude[1], Sebastian Randel[1], Christian Koos[1,3*]

[1]Institute of Photonics and Quantum Electronics (IPQ), Karlsruhe Institute of Technology (KIT), 76131 Karlsruhe, Germany.
[2]Institute of Radio Frequency Engineering and Electronics (IHE), Karlsruhe Institute of Technology (KIT), 76131 Karlsruhe, Germany.
[3]Institute of Microstructure Technology (IMT), Karlsruhe Institute of Technology (KIT), 76344 Eggenstein-Leopoldshafen, Germany.

*Corresponding authors: christoph.fuellner@kit.edu; christian.koos@kit.edu;

This document provides supplementary information to "Photonic-Electronic Arbitrary-Waveform Generation Using Quadrature Multiplexing and an Active Optical-Phase Stabilization". In Section S1, we derive the mathematical relations that form the theoretical base of quadrature multiplexing and that are key to implementing a photonic-electronic arbitrary-waveform generator (PE-AWG) offering twice the bandwidth of the underlying electronic DACs. Section S2 describes the experimental setup used for the proof-of-concept demonstration of the PE-AWG as well as the digital signal processing (DSP) techniques for synthesizing the drive signals $u_\mathrm{I}(t)$ and $u_\mathrm{Q}(t)$ of the in-phase / quadrature modulator (IQM) and for evaluating the PE-AWG performance. Furthermore, Section S2 provides more details on the active optical phase stabilization, covering both the underlying concepts and the accuracy achieved in the experiment. Section S3 gives details of the PE-AWG characterization based on broadband target waveforms, including an analysis of the frequency-dependent signal-to-noise-and-distortion ratio (SNDR), a performance comparison with the underlying fully-electronic AWG (Keysight M8194A), and with other electronic AWG competing schemes. This section further discusses the performance stability of the proposed PE-AWG scheme and the importance of the active phase control. Section S4 summarizes results from single-tone and two-tone measurements. In Section S5, we finally identify various ways to further improve the PE-AWG implementation and performance in the future.

S1 Mathematical description of quadrature multiplexing

In this section we provide a mathematical model of the PE-AWG by deriving the equations that form the basis of quadrature multiplexing. Section S1.1 is a recapitulation of coherent reception in general, Section S1.2 focuses on heterodyne down-conversion in particular, and Section S1.3 explains how the target waveform is generated at the PE-AWG output by appropriate synthesis of the IQM drive signals $u_\mathrm{I}(t)$ and $u_\mathrm{Q}(t)$ and by precise control of the optical phase relation between the resulting broadband optical waveform and the local oscillator (LO) tone. Section S1.4 elaborates on the mathematical base of the active optical phase control concept.

S1.1 Coherent reception

As illustrated in Fig. 1(a) of the main manuscript, a coherent receiver consisting of a 90° optical hybrid (OH) and a high-speed balanced photodetector (BPD1) is employed for heterodyne down-conversion of the broadband optical waveform $a_s(t) = \Re\{\underline{a}_s(t)\}$ with the LO tone $a_\mathrm{LO}(t) = \Re\{\underline{a}_\mathrm{LO}\} = \Re\{A_\mathrm{LO}\,\exp(\mathrm{j}(2\pi f_1 t + \phi_\mathrm{LO}(t)))\}$. Since coherent reception is associated with a linear relation between the broadband optical waveform at the input of the BPD and its electrical output signal, complex notation can be used from here on. Neglecting noise, the optical fields at the outputs of the OH labelled 'I^+' and 'I^-' read [1]

$$\underline{a}_{\mathrm{I}^+}(t) = \sqrt{1-\varepsilon}\,\underline{a}_s(t) + \sqrt{\varepsilon}\,\underline{a}_\mathrm{LO}(t) \tag{S.1}$$

$$\underline{a}_{\mathrm{I}^-}(t) = -\sqrt{\varepsilon}\,\underline{a}_s(t) + \sqrt{1-\varepsilon}\,\underline{a}_\mathrm{LO}(t), \tag{S.2}$$

with ε being the power splitting ratio of the two OH arms. In the following, we assume the ideal case, $\varepsilon = 0.5$. Each of the two photodiodes of BPD1 acts as a square-law detector leading to a photocurrent that comprises three mixing terms and that depends on the impulse response of the respective signal paths h_{I^+} and h_{I^-},

$$i_{\mathrm{I}^+}(t) \propto h_{\mathrm{I}^+} * \left[|\underline{a}_{\mathrm{I}^+}(t)|^2\right] = h_{\mathrm{I}^+} * \left[\underline{a}_{\mathrm{I}^+}(t)\underline{a}^*_{\mathrm{I}^+}(t)\right] = h_{\mathrm{I}^+} * \left[\left(\frac{1}{\sqrt{2}}\underline{a}_s(t) + \frac{1}{\sqrt{2}}\underline{a}_\mathrm{LO}(t)\right)\left(\frac{1}{\sqrt{2}}\underline{a}^*_s(t) + \frac{1}{\sqrt{2}}\underline{a}^*_\mathrm{LO}(t)\right)\right] \tag{S.3}$$

$$= h_{\mathrm{I}^+} * \left[\frac{1}{2}|\underline{a}_s(t)|^2 + \frac{1}{2}|\underline{a}_\mathrm{LO}(t)|^2 + \frac{1}{2}\left(\underline{a}_s(t)\underline{a}^*_\mathrm{LO}(t) + \underline{a}^*_s(t)\underline{a}_\mathrm{LO}(t)\right)\right] \tag{S.4}$$

$$= h_{\mathrm{I}^+} * \left[\frac{1}{2}|\underline{a}_s(t)|^2 + \frac{1}{2}|\underline{a}_\mathrm{LO}(t)|^2 + \Re\{\underline{a}_s(t)\underline{a}^*_\mathrm{LO}(t)\}\right], \tag{S.5}$$

$$i_{\mathrm{I}^-}(t) \propto h_{\mathrm{I}^-} * \left[|\underline{a}_{\mathrm{I}^-}(t)|^2\right] = h_{\mathrm{I}^-} * \left[\underline{a}_{\mathrm{I}^-}(t)\underline{a}^*_{\mathrm{I}^-}(t)\right] \tag{S.6}$$

$$= h_{\mathrm{I}^-} * \left[\frac{1}{2}|\underline{a}_s(t)|^2 + \frac{1}{2}|\underline{a}_\mathrm{LO}(t)|^2 - \Re\{\underline{a}_s(t)\underline{a}^*_\mathrm{LO}(t)\}\right]. \tag{S.7}$$

Here $*$ refers to a convolution operation and * denotes the complex conjugate of respective complex-valued variables. While the third expression in the last line of each relation (signal-LO beating) contains the desired information and the second expression (LO-LO beating) results in a constant current (DC), the first one represents unwanted signal-signal mixing products that spectrally overlap with the down-converted data, thereby deteriorating the information. This effect is commonly known as signal-signal beat interference (SSBI). An ideal BPD with infinite common-mode rejection ratio (CMRR), $h_\mathrm{BPD,1} = h_{\mathrm{I}^+} = h_{\mathrm{I}^-}$, and perfect temporal coincidence of the beating terms of the outputs $i_{+\mathrm{I}^+}$ and i_{I^-} of the two photodiodes ("no skew") fully eliminates such SSBI from the resulting BPD output current $i_\mathrm{BPD,1}(t)$ by subtraction,

$$i_\mathrm{BPD,1}(t) \propto i_{\mathrm{I}^+}(t) - i_{\mathrm{I}^-}(t) = 2h_\mathrm{BPD,1} * \left[\Re\{\underline{a}_s(t)\underline{a}^*_\mathrm{LO}(t)\}\right] \tag{S.8}$$

S1.2 Heterodyne down-conversion

Quadrature multiplexing relies on heterodyne down-conversion of a well-defined optical IQ signal $\underline{a}_s(t) = (u_\mathrm{I}(t) + \mathrm{j}u_\mathrm{Q}(t))\exp\left(\mathrm{j}(2\pi f_2 t + \phi_s(t)\right)$ covering an optical bandwidth $2B$ with an LO tone $\underline{a}_\mathrm{LO}(t) = A_\mathrm{LO}\,\exp(\mathrm{j}(2\pi f_1 t + \phi_\mathrm{LO}(t)))$ that sits exactly at the spectral edge of the IQ signal, $f_2 = f_1 + B$, see Inset ② in Fig. 1(a) of the main manuscript. The time-dependent phases $\phi_s(t)$ and $\phi_\mathrm{LO}(t)$ represent random phase fluctuations and drifts due to imperfect light soruces and due to thermal drifts in the underlying optical setups. By inserting the expressions for $\underline{a}_s(t)$ and $\underline{a}_\mathrm{LO}$ into Eq. (S.8), we obtain

$$i_\mathrm{BPD,1}(t) \propto h_\mathrm{BPD,1} * \left[\Re\{(u_\mathrm{I}(t) + \mathrm{j}u_\mathrm{Q}(t))\exp\left(\mathrm{j}(2\pi B t + \Delta\phi(t)\right)\}\right], \quad \Delta\phi(t) = \phi_s(t) - \phi_\mathrm{LO}(t), \tag{S.9}$$

with $\underline{A}_s(t) = (u_I(t) + ju_Q(t)) \exp(j2\pi Bt)$, see Eq. (1) of the main manuscript. In case the low-pass characteristics of the BPD $h_{BPD,1}$ are of negligible impact on the photocurrent $i_{BPD,1}(t)$ or are fully compensated by proper predistortion, the resulting BPD output current $i_{BPD,1}(t)$ can be written as

$$i_{BPD,1}(t) \propto \Re\{\underline{A}_s(t) \exp(j(\Delta\phi(t)))\}. \tag{S.10}$$

S1.3 Target waveform generation

As discussed in the subsection "Photonic-electronic arbitrary-waveform generator (PE-AWG) concept" of Section 2 of the main manuscript, the waveform generated at the output of the PE-AWG is identical to the target waveform under certain conditions only. First, an active optical phase stabilization must ensure $\Delta\phi \, \epsilon \, \{0, 2\pi, 4\pi \ldots\}$ as the output waveform sensitively depends on the optical phase relation $\Delta\phi$ between the LO tone and the optical IQ signal. In the remaining part of this document, we consider, without loss of generality, only the case $\Delta\phi = 0$ as a representation of an ideally stabilized optical phase. As a second condition, the real-valued drive signals $u_I(t)$ and $u_Q(t)$ of the IQM must be synthesized to satisfy the relations $s(t) = \Re\{\underline{A}_s(t)\}$ and $\widetilde{\underline{A}}_s(f) = 0$ for $f < 0$. Together, these relations imply $\underline{A}_s(t) = s(t) + j\mathcal{H}\{s(t)\}$ with $\mathcal{H}$ being the Hilbert transform

$$\mathcal{H}\{s(t)\} = \frac{1}{\pi} \mathcal{P} \int_{-\infty}^{\infty} \frac{s(\tau)}{t - \tau} d\tau, \tag{S.11}$$

where $\mathcal{P}$ is the Cauchy principal value of the underlying integral. In the following, we insert the two conditions into Eq. (S.10) one after the other to show that the correct target waveform is obtained at the PE-AWG output. With $\underline{A}_s(t) = s(t) + j\mathcal{H}\{s(t)\}$, we can rewrite Eq. (S.10) to

$$\begin{aligned} i_{BPD,1} &\propto \Re\{(s(t) + j\mathcal{H}\{s(t)\}) (\cos(\Delta\phi(t)) + j \sin(\Delta\phi(t)))\} \\ &\propto \Re\{s(t) \cos(\Delta\phi(t)) - \mathcal{H}\{s(t)\} \sin(\Delta\phi(t)) + j\mathcal{H}\{s(t)\} \cos(\Delta\phi(t)) + js(t) \sin(\Delta\phi(t))\} \\ &\propto s(t) \cos(\Delta\phi(t)) - \mathcal{H}\{s(t)\} \sin(\Delta\phi(t)). \end{aligned} \tag{S.12}$$

This expression clearly indicates that the distortion of the PE-AWG output waveform introduced by the phase offset $\Delta\phi$. For a perfectly working active phase stabilization, $\Delta\phi = 0$, the equation simplifies to

$$i_{BPD,1} \propto s(t). \tag{S.13}$$

Note that a reference tone (RT) with a real-valued amplitude s_{RT} needs to be added at the spectral edge of the complex-valued envelope $\underline{A}_s(t)$ of the target waveform $s(t)$ for the active phase stabilization to be functional as explained in the Section S1.4 below. Modifying Eqs. (S.12)-(S.13) accordingly yields

$$\begin{aligned} i_{BPD,1} &\propto s(t) \cos(\Delta\phi(t)) - \mathcal{H}\{s(t)\} \sin(\Delta\phi(t)) + s_{RT} \cos(\Delta\phi(t)), \\ \lim_{\Delta\phi \to 0} i_{BPD,1} &= s(t) + s_{RT} \end{aligned} \tag{S.14}$$

The photocurrent $i_{BPD,1}$ then contains an additional DC component that is maximized for $\Delta\phi \to 0$. It should be noted that this complicates generation of target waveforms with specific DC levels with our current PE-AWG implementation because any DC component in the target waveform would interfere with the DC component s_{RT}, see Eqs. (S.14). These limitations can be overcome by slight modifications of the active phase-stabilization concept, see Section S2.3 for further details.

S1.4 Low-speed BPD output (active optical phase stabilization)

The RT described in the previous section is intentionally added at the spectral edge of the digital version $\underline{A}_{s,n}$[1] of the complex-valued envelope $\underline{A}_s(t)$ during digital signal synthesis and then further processed. The RT is then converted to the analog domain and modulated onto the optical carrier at f_2 along with the IQ drive signals $u_I(t)$ and $u_Q(t)$ associated with the targeted optical waveform. The resulting optical tone then interferes with the LO tone, thereby generating a

[1] $\underline{A}_{s,n} = \underline{A}_s(nT_s)$ with T_s being the sampling period. Throughout the document, all variables with subscript n correspond to discrete-time signals.

beat signal with an amplitude that depends on the relative phase $\Delta\phi(t)$. This error signal is fed to an active optical phase stabilization circuit, which provides a feedback signal to continuously adjust the optical phase of the LO tone $\phi_{\mathrm{LO}}(t)$, ideally via an endless optical phase shifter, see subsection "Active optical-phase stabilization" in Section 2 of the main manuscript. In analogy to the derivation described in Section S1.3 above, we can derive the following expression for the output of the low-speed BPD (BPD2) deployed within the feedback loop and highlighted in turquoise in Fig. 1(a) and Fig. 2 of the main manuscript:

$$
\begin{aligned}
i_{\mathrm{BPD},2} &\propto h_{\mathrm{BPD},2} * \left[\Im\{(\underline{A}_s(t) + s_{\mathrm{RT}})\exp(\mathrm{j}\Delta\phi(t))\}\right] && \text{(S.15)}\\
&\propto h_{\mathrm{BPD},2} * \left[\Im\{(s(t) + s_{\mathrm{RT}})(\cos(\Delta\phi(t)) - \mathcal{H}\{s(t)\}\sin(\Delta\phi(t))) \right. && \text{(S.16)}\\
&\qquad \left. + \mathrm{j}\,(\mathcal{H}\{s(t)\}\cos(\Delta\phi(t)) + (s(t) + s_{\mathrm{RT}})(\sin(\Delta\phi(t)))\}\right] && \text{(S.17)}\\
&\propto h_{\mathrm{BPD},2} * \left[\mathcal{H}\{s(t)\}\cos(\Delta\phi(t)) + s(t)\sin(\Delta\phi(t)) + s_{\mathrm{RT}}\sin(\Delta\phi(t))\right] && \text{(S.18)}
\end{aligned}
$$

For the active optical phase stabilization, only the term $s_{\mathrm{RT}}\sin(\Delta\phi(t))$ is of interest, as it represents the beating of the LO with the RT. Since the optical phase drift is rather slow, its spectral coverage is limited to low frequencies, and a bandwidth in the high kHz or low MHz range is sufficient for BPD2 and the subsequent control circuitry. While the expression of interest $s_{\mathrm{RT}}\sin(\Delta\phi(t))$, which is related to the slowly-varying phase drift, remains essentially unaffected by $h_{\mathrm{BPD},2}$, the remaining part $\mathcal{H}\{s(t)\}\cos(\Delta\phi(t)) + s(t)\sin(\Delta\phi(t))$ is strongly suppressed by the low-pass characteristics of BPD2, in particular when the target waveform is mean-free. A residual portion of the latter part is still transmitted by the low-pass $h_{\mathrm{BPD},2}$ and interferes with the LO-RT beating. This leads to a distortion of the feedback signal, which can be modeled by a noise term $n_{\mathrm{data}}(t)$ in the phase control. Moreover, in a practical implementation with an imperfect BPD2 featuring a limited CMRR, additional interference is, e.g., caused by residual SSBI and noise mixing products. In summary, we can write the voltage fed as an input to the phase control circuit as

$$
u_{\mathrm{BPD},2} \approx C\sin(\Delta\phi(t)) + n_{\mathrm{data}}(t) \approx C\sin(\Delta\phi(t)), \quad \text{for } \Delta\phi(t) \ll 1, \tag{S.19}
$$

see also subsection "Active optical-phase stabilization" in Section 2 of the main manuscript. In this relation, C is a proportionality factor that depends on the power of the RT. The impact of the distortion $n_{\mathrm{data}}(t)$ on the phase control becomes negligible for large C, i.e., strong reference tones, leading to a feedback signal with a high signal-to-noise ratio (SNR). In case of negligible distortions $n_{\mathrm{data}}(t)$, the active optical phase stabilization can simply be configured to minimize the error signal $u_{\mathrm{BPD},2}$. Note that, in case the target waveform $s(t)$ is not mean-free, the distortions $n_{\mathrm{data}}(t)$ will contain DC components that cannot be suppressed by reducing the bandwidth of the low-pass $h_{\mathrm{BPD},2}$ or of a subsequent loop filter that is part of the control circuit. If such waveforms are to be synthesized, the known DC part of the signal must be subtracted by a properly configured control system. Similarly, the control system might be designed to account for other waveform-dependent distortions of the feedback signal that may, e.g., originate from imperfect balancing of BPD2. Our current implementation of the phase control partially accounts for these effects, see Section S2.3 below for a more detailed description.

S2 Detailed experimental implementation

This section describes the proof-of-concept implementation of the PE-AWG. In Section S2.1 the experimental setup consisting of discrete hardware components is discussed. Compared to subsection "Experimental implementation" of Section 2 of the main manuscript, more details are provided with respect to polarization alignment, optical power control, optical filtering, and the frequency shift applied to f_1 in the upper branch of the setup after the 50/50 splitter seen in Fig. 2 of the main manuscript. Section S2.2 reviews the digital pre-processing that is essential for synthesis of a target waveform with the PE-AWG as well as the digital signal processing (DSP) chain used to evaluate the PE-AWG performance. Section S2.3 explains our implementation of the electro-optic phase-locked loop (PLL) that is used to actively control the error signal of Eq. (S.19) to zero and thus to achieve a time-stable phase relation $\Delta\phi(t)$ between the LO tone and the broadband IQ waveform during heterodyne down-conversion. In Section S2.3.1, we present an analysis of the optical phase fluctuations observed in our setup for three cases: an open control loop, i.e., a random optical phase $\Delta\phi(t)$, a loop closed with a digital controller, and a loop closed with an analog control element.

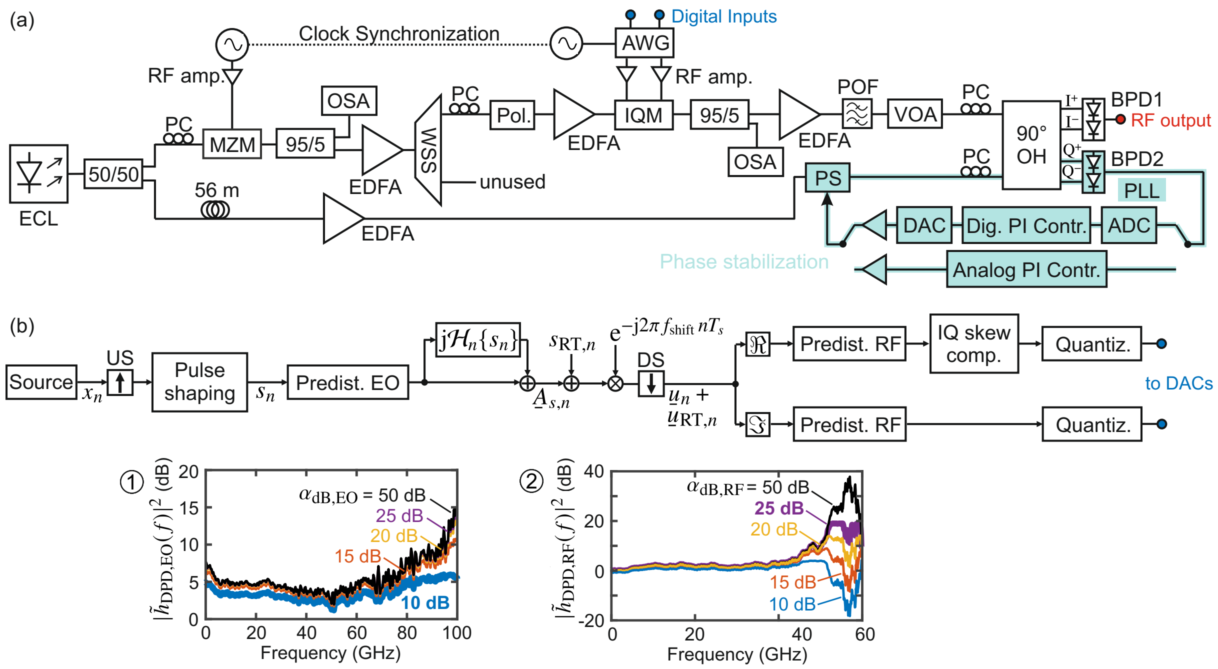

Fig. S1: Experimental implementation of the proposed photonic-electronic arbitrary-waveform generator (PE-AWG) based on discrete hardware components. **(a)** Experimental setup. A low-phase-noise optical tone is generated by an external-cavity laser (ECL) and split in two portions. One portion is routed to the lower arm of the setup and is directly used as an LO tone for down-conversion, whereas the other portion is sent to the upper arm, where a frequency shift by $f_{\text{shift}} \leq B$ is applied by a sine-wave driven Mach-Zehnder modulator (MZM) and a subsequent wavelength-selective switch (WSS) acting as a bandpass filter to suppress the unwanted sidebands and the residual carrier. The frequency-shifted tone is sent to an IQM, which is driven by a pair of channels of a fully-electronic AWG. The MZM drive signal is time-synchronized with the AWG clock. Before superimposing the optical IQ signal with the LO tone in a 90° optical hybrid (90° OH), it is sent through a programmable optical filter (POF) to remove optical out-of-band noise, and a variable optical attenuator (VOA) is used to control the LO-to-signal power ratio (LOSPR). Erbium-doped fiber amplifiers (EDFAs) compensate insertion and modulation losses at various positions in the setup, and polarization controllers (PCs) and polarizers (Pol.) are used to align polarizations. The coherent down-conversion is performed by a commercially available 100-GHz BPD, which is coupled to the in-phase outputs ('I$^+$' and 'I$^-$') of the OH [3]. An electro-optic phase-locked loop (PLL) highlighted in turquoise is used for active stabilization of the relative optical phase between the LO and the optical carrier of the IQ waveform. To this end, the two remaining quadrature outputs ('Q$^+$' and 'Q$^-$') of the OH are used to tap monitoring signals to a low-speed BPD (BPD2), whose output is proportional to $\sin(\Delta\phi(t))$, see Eq. (S.19). The error signal is then digitized (analog-to-digital converter, ADC) and fed to a digital PI controller generating a feedback signal. The digital feedback signal is then converted to an analog RF signal in a low-speed digital-to-analog converter (DAC), amplified and then sent to a piezo-driven fiber-stretecher that adjusts the LO phase. Alternatively, a fully-analog PI controller can be used, see Section S2.3 for details. We generate PAM signals with up to eight amplitude levels at symbol rates between 100 GBd and 200 GBd. **(b)** Block diagram of the DSP chain for digital synthesis of the IQM drive signals $\Re\{\underline{u}(t)\}$ and $\Im\{\underline{u}(t)\}$. As explained in Fig. 1(c), a digital version of the target waveform s_n is generated. Adding its Hilbert transform as an imaginary part eliminates the negative-frequency components. The analytic signal $\underline{A}_{s,n}$ is then frequency-shifted to be centered around zero frequency, and the real and imaginary part of the resulting digital signal $\underline{u}_n$ are fed to the two DAC modules. In addition to the processing blocks shown in Fig. 1(c) of the main manuscript, the sketch shows predistortion filters (Predist. EO and Predist. RF), the IQ time-skew compensation, and quantization stages, that prepare the signal for being fed to the two DACs. The first stage of predistortion filters (Predist. EO) accounts for the frequency response of the electro-optic components, i.e., the IQM and the high-speed BPD at the PE-AWG output (BPD1), whereas the second stage (Predist. RF) compensates the individual frequency responses of the two DAC modules in the AWG, the two RF amplifiers, and the coaxial RF cables to the IQM. Both predistortion filters are derived from a channel response estimated with a least-squares method – the resulting transfer function are indicated in Inset 1 and Inset 2. The quantities $\alpha_{\text{dB,EO}}$ and $\alpha_{\text{dB,RF}}$ are free parameters that are proportional to the SNR observed at the output of the respective hardware components and that allow to tune each of the predistortion filters from a zero-forcing implementation to a solution that minimizes the impact of noise added along the signal path through the system. An heuristic optimization procedure is used to find appropriate values of $\alpha_{\text{dB,EO}} = 10\,\text{dB}$ and $\alpha_{\text{dB,RF}} = 25\,\text{dB}$, that minimize the mean square error (MSE) at the output of the PE-AWG. The transfer functions obtained from this optimization are indicated by the the bold lines in the two insets of the block diagram.

S2.1 Experimental setup

This section describes the experimental setup used for the proof-of-concept demonstration of the PE-AWG with discrete hardware components. We use this implementation to generate two-level, four-level, and eight-level pulse-amplitude modulation signals (PAM2, PAM4, and PAM8) with symbol rates f_s of up to 200 GBd as exemplary target waveforms.

Note that the target waveforms rely on pulse shapes with a root-raised-cosine (RRC) amplitude spectrum and a roll-off ρ=0.05. The full setup is depicted in Fig. S1(a). The optical carrier and the LO tone required for the quadrature multiplexing scheme are derived from a single external-cavity laser (ECL) with a linewidth below 100 kHz. The ECL emits an optical tone at frequency f_1, which is then split in two identical copies by a 3-dB (50/50) coupler. The tone in the upper arm of the setup is frequency-shifted to a frequency $f_2 = f_1 + f_{\text{shift}}$, where the frequency shift f_{shift} is dictated by the target waveform and limited by the bandwidth B of each individual electronic DAC (contained in the electronic AWG), $f_{\text{shift}} \leq B$, see subsection "Photonic-electronic arbitrary-waveform generator (PE-AWG) concept" of Section 2 of the main manuscript. The frequency shift is accomplished by a sine-wave-driven MZM, operated close to the null point, and a subsequent optical bandpass filter with steep transitions suppressing the unwanted sideband at frequency $f_1 - f_{\text{shift}}$ as well as the residual carrier tone at frequency f_1. In our experiments, we use a wavelength-selective switch (WSS) for optical filtering. An erbium-doped fiber amplifier (EDFA) is used in the upper arm to compensate for the modulation loss as well as for the optical insertion loss of the MZM and the WSS. The optical carrier at f_2 is then modulated in amplitude and phase using a commercially-available single-polarization $LiNbO_3$ IQM with a 3-dB bandwidth of ~30 GHz. The associated drive signals are generated by a pair of channels of a fully-electronic AWG (Keysight M8194A) based on CMOS-DACs, each with a sampling rate of 120 GSa/s and a nominal analog bandwidth B of about 45 GHz [2]. The transmitter DSP that is used to generate the digital inputs to the electronic AWG follows the principles explained in subsection "Digital synthesis of IQM drive signals" of Section 2 of the main manuscript and is discussed in more detail in Section S2.2.1. At the output of the AWG ports, we use linear RF amplifiers (RF amp.) with 23 dB gain to increase the electrical signal power prior to feeding the drive signals to the IQM. After the IQM, another EDFA is used to compensate for the insertion loss and modulation loss. We further include a programmable optical filter (POF) in the upper fiber arm of our setup to remove out-of-band noise. In our IM/DD data transmission experiment, see subsection "Application example: IM/DD fiber transmission experiment" of Section 2 of the main manuscript, we also use the POF as an optical pre-equalizer to adjust the spectrum of the waveform at the high-speed PE-AWG output. The optical input powers to the OH as well as the LO-to-signal power ratio (LOSPR) are adjusted by an EDFA in the lower and by a variable optical attenuator (VOA) in the upper arm. Polarization controllers (PC) are used to align the polarization of the LO tone, the optical carrier, and the IQ signals throughout the setup – this would become obsolete in an integrated implementation of the PE-AWG. For coherent down-conversion, we rely on a commercially available 100-GHz indium-phosphide waveguide-integrated BPD [3] with two approximately length-matched fiber pigtails, each connected to one of the photodiodes. Optical spectrum analyzers (OSAs) allow to capture the optical spectrum at various points of the experimental setup. An electro-optic PLL (turquoise) is used for active stabilization of the relative optical phase $\Delta\phi(t)$ between the LO and the optical carrier of the IQ signal, see Section S2.3 for detail.

S2.2 Digital signal processing (DSP)

This section details on the DSP techniques used for the experiments presented in the main manuscript. Section S2.2.1 explains the practical implementation of the digital pre-processing in the PE-AWG, see Fig. 1(c) of the main paper, with special emphasis on the pre-compensation of the frequency-dependent signal transfer characteristics of the underlying hardware components such as the electronic AWG, the IQM, or the BPD. In Section S2.2.2, we discuss how a suitable choice of the exact frequency shift f_{shift} can help in balancing the signal quality at the PE-AWG output over the full bandwidth range. The DSP chain used to process the various data signals and to benchmark the PE-AWG performance in our back-to-back experiments is discussed in Section S2.2.3. In Section S2.2.4, we explain how the the SNDR is calculated from received PAM symbols (SNR_{PAM}) to serve as a performance metric throughout our PAM waveform analyses in the main manuscript and in the discussion provided in Section S3 of this supplementary information.

S2.2.1 Digital pre-processing

Appropriate digital pre-processing is essential for reliable generation of the target waveform through an appropriately predistorted complex-valued envelope $\underline{A}_s(t)$ of the optical signal generated by the associated IQM drive signals $u_{\text{I}}(t)$ and $u_{\text{Q}}(t)$, see subsection "Digital synthesis of IQM drive signals" of Section 2 of the main manuscript. A block diagram of the transmitter DSP is shown in Fig. S1(b). The signal source on the very left is configured to generate a pseudo-random pattern $\mathbf{x}$ consisting of $\sim$ 200 000 PAM symbols with the Mersenne-Twister algorithm [4]. The discrete-time PAM signal x_n is obtained as a periodic repetition of the symbol pattern $\mathbf{x}$ and is initially represented by one sample per symbol. It is then upsampled (US) to twice the sampling rate of the electronic AWG with an upsampling factor that depends on the symbol rate, before being shaped (Pulse shaping) to obtain an RRC amplitude spectrum with a roll-off that is defined by a coefficient ρ. For the results discussed in the main manuscript, we use ρ=0.05, which corresponds to an

excess bandwidth of 5%. The output of the pulse-shaping filter is a discrete-time version s_n of the broadband electronic target waveform $s(t)$ to be generated by the PE-AWG. This real-valued signal is processed in a first predistortion filter compensating for the frequency response of the electro-optic components (Predist. EO), i.e., the high-speed BPD at the PE-AWG output (BPD1). Adding the Hilbert transform of the resulting time-domain signal yields the corresponding analytic discrete-time signal $\underline{A}_{s,n}$, see Eq. (4) in the main manuscript. For our proof-of-concept PE-AWG, we have used a Matlab-internal implementation of the Hilbert transform (Matlab command 'imag(hilbert(...))'). In this scheme, the Hilbert transform is applied in frequency domain after performing an N-point Fast Fourier transform (FFT). In our case, N was identical to the length of our target waveform pattern, e.g., 32768 samples. This approach could also be used when leveraging our PE-AWG approach for high-speed test and measurement equipment, where digital pre-processing can be done offline and only the final result of the signal synthesis, i.e., the discrete-time versions $u_\mathrm{I}(t)$ and $u_\mathrm{Q}(t)$ of the IQM drive signals, is sent to the DAC hardware. In contrast to that, real-time implementations need to rely on reduced FFT sizes or time-domain implementations of the Hilbert. We have previously investigated the complexity and performance of such Hilbert-transform implementations and found that very efficient time-domain schemes using only 32 non-zero coefficients still offer decent performance while significantly reducing the computational complexity [5]. In the light of these results, we do not consider the hardware complexity of the Hilbert transform as a main contributor to the overall PE-AWG complexity. After generating the analytic signal, a discrete-time version of the reference tone (RT) with real-valued amplitude $s_{\mathrm{RT},n}$ required for the phase control is added, see Sections S1.4 and S2.3 for details. As a next step, a frequency shift by f_shift is applied to the digital signal, where f_shift corresponds to the frequency shift applied to the MZM, see subsection "Photonic-electronic arbitrary-waveform generator (PE-AWG) concept" of Section 2 and subsection "Experimental implementation" of Section 2 of the main manuscript. As a consequence, the optical LO tone and the RT will match in frequency before heterodyne down-conversion in BPD1 and BPD2. For a sampling period T_s, the frequency shift results in signals $\underline{u}_n = \underline{A}_{s,n} \exp\left(-\mathrm{j}(2\pi f_\mathrm{shift}\, nT_s)\right)$ and $\underline{u}_{\mathrm{RT},n} = s_{\mathrm{RT},n} \exp\left(-\mathrm{j}(2\pi f_\mathrm{shift}\, nT_s)\right)$. After down-sampling (DS) to the sampling rate of the AWG, the complex-valued signal $\underline{u}_n$ is split into its real part $u_{\mathrm{I},n}$ and imaginary part $u_{\mathrm{Q},n}$, and a second set of independent predistortion filters (Predist. RF) is applied to account for the individual frequency responses of the two DAC modules in the AWG, the two associated RF amplifiers, and coaxial RF cables to the IQM, and the electrical I and Q interfaces of the IQM. Note that the frequency-shifted RT $\underline{u}_{\mathrm{RT},n}$ is subjected to this predistortion step as well. Afterwards, IQ skew caused by path-length mismatches in the two RF paths to the IQM is digitally pre-compensated. The two resulting signals are then quantized and fed to the pair of DACs, where each DAC corresponds to one channel of the electronic AWG, to finally generate the two analog signals $u_\mathrm{I}(t) + \Re\{\underline{u}_\mathrm{RT}(t)\}$ and $u_\mathrm{Q}(t) + \Im\{\underline{u}_\mathrm{RT}(t)\}$ for driving the IQM.

With respect to the digital predistortion of the RF components, the discrete impulse response of each RF path is obtained from a least-squares channel estimation in an electrical back-to-back scenario, where the RF cables are directly connected to a 100-GHz real-time oscilloscope featuring a sampling rate of 256 GSa/s. The discrete-time impulse responses $\mathbf{h}_\mathrm{RF}$ of the RF components and $\mathbf{h}_\mathrm{EO}$ of the electro-optic components from the IQM input to the BPD comprise L_DPD sampling points each and are calculated as $\mathbf{h} = (\mathbf{X}^\mathrm{T}\mathbf{X})^{-1}\mathbf{X}^\mathrm{T}\mathbf{r}$ [6, 7]. In this relation, $\mathbf{r}$ is an N-element vector of the received samples after resampling, timing recovery, and an RRC receive filter, $\mathbf{X}$ is a $N \times L_\mathrm{DPD}$ sliding-window matrix generated from one period of the symbol pattern $\mathbf{x}$, and the superscript T denotes the matrix transpose, see [6] for details. Similarly, the combined impulse response $\mathbf{h}_\mathrm{EO}$ of the electro-optic components from the IQM input to the BPD output is estimated when connecting the output of the PE-AWG as a whole to the oscilloscope, whereby the frequency-dependent attenuation of the RF components are already pre-compensated. For recording the sample vector $\mathbf{r}$ in the experiment, the phase control is continuously activated. The length of the extracted impulse response is chosen to $L_\mathrm{DPD} = 600$ samples both for $\mathbf{h}_\mathrm{EO}$ and for $\mathbf{h}_\mathrm{RF}$, which is needed to account for multiple backreflections in the RF cables between the RF amplifiers and the IQM [2, 6, 8–10]. In an integrated version of the PE-AWG, where the driver amplifiers and the IQM are combined in a common module, the impulse responses can usually be truncated after a much smaller number of coefficients. The coefficients of the respective digital predistortion (DPD) filters $\mathbf{h}_\mathrm{DPD}$ are found as $\mathbf{h}_\mathrm{DPD} = (\mathbf{H}^\dagger\mathbf{H} + \alpha^{-1}\mathbf{I})^{-1}\mathbf{H}^\dagger$[11, 12], which is known as a minimum mean square error (MMSE) predistortion filter, where $\mathbf{H} = \left(\left(\mathbf{0}_0^\mathrm{T}, \mathbf{h}^\mathrm{T}, \mathbf{0}_{L_\mathrm{DPD}-1}^\mathrm{T}\right)^\mathrm{T} \left(\mathbf{0}_1^\mathrm{T}, \mathbf{h}^\mathrm{T}, \mathbf{0}_{L_\mathrm{DPD}-2}^\mathrm{T}\right)^\mathrm{T} \cdots \left(\mathbf{0}_{L_\mathrm{DPD}-1}^\mathrm{T}, \mathbf{h}^\mathrm{T}, \mathbf{0}_0^\mathrm{T}\right)^\mathrm{T} \right)$ is the $(2L_\mathrm{DPD} - 1) \times L_\mathrm{DPD}$ channel matrix of either the RF or the EO components with $\mathbf{h}$ standing for $\mathbf{h}_\mathrm{EO}$ or $\mathbf{h}_\mathrm{RF}$, $\mathbf{0}_m$ denoting a vector of m zeros, $\mathbf{I}$ is the $L_\mathrm{DPD} \times L_\mathrm{DPD}$ identity matrix, and $\dagger$ denotes the conjugate transpose. The free parameter $\alpha = 10^{\alpha_\mathrm{dB}/10}$ is proportional to the SNR observed at the output of the respective hardware components and allows to tune each of the two predistortion filters from a zero-forcing filter, that minimizes intersymbol interference at the PE-AWG output by simply inverting the frequency-dependent roll-off of the band-limited channel ($\alpha \to \infty$), to a solution that minimizes the impact of noise added along the signal path ($\alpha \to 0$). This flexbility is necessary because any bandlimited channel $\mathbf{H}$ turns $\mathbf{h}_\mathrm{DPD}$ into a high pass filter, which increases the peak-to-average power ratio (PAPR) and reduces the overall average signal power for a

maximum signal amplitude that is dictated by the range of the DAC, thereby effectively limiting the SNR of the generated analog signal. Note that in case the intersymbol interference caused by bandwidth limitations is negligible, $\mathbf{H}^{\dagger}\mathbf{H}$ becomes a diagonal matrix and the MMSE filter approaches the matched filter. We heuristically optimize the predistortion filters by maximizing the signal quality at the PE-AWG output when tuning the free parameters $\alpha_{\mathrm{dB,EO}}$ and $\alpha_{\mathrm{dB,RF}}$ for the EO and RF components, respectively, in a joint process. This concept is similar to the one proposed in [13] (Section 3.1.2). We find $\alpha_{\mathrm{dB,EO}} = 10\,\mathrm{dB}$ and $\alpha_{\mathrm{dB,RF}} = 25\,\mathrm{dB}$ as suitable solutions. The frequency response of the predistortion filters is finally obtained as the discrete Fourier transforms $\tilde{\mathbf{h}}_{\mathrm{DPD,EO}}$ and $\tilde{\mathbf{h}}_{\mathrm{DPD,RF}}$ of the respective impulse responses, see Insets 1 and 2 of Fig. S1(b) for plots of the resulting amplitude transfer functions that were obtained for different values of $\alpha_{\mathrm{dB,EO}}$ and $\alpha_{\mathrm{dB,RF}}$. The bold lines correspond to the finally chosen predistortion filters obtained for $\alpha_{\mathrm{dB,EO}}$ =10 dB and $\alpha_{\mathrm{dB,RF}}$ =25 dB. As seen in the left inset, the roll-off of the EO components is only partially pre-compensated. This is necessary as not to cause a too pronounced high-pass characteristic. For the pre-emphasis of the RF components, however, a fairly high value has been chosen for α_{dB} to effectively combat all distortions up to 52 GHz, see the purple curve in the right inset of Fig. S1(b). Note that the pre-compensation treats only linear impairments of the respective channel. Note also that the estimated discrete-time channel impulse responses also include imperfections of the 100-GHz oscilloscope, which might lead to a slightly suboptimal predistortion when considering only the bare PE-AWG without the oscilloscope.

S2.2.2 Choice of a suitable frequency shift

As described in Section S2.1, the frequency shift f_{shift} to be applied during digital signal synthesis is dictated by the target waveform and limited by the bandwidth B of the underlying electronic DAC (i.e. AWG channel), $f_{\mathrm{shift}} \leq B$. In subsection "Photonic-electronic arbitrary-waveform generator (PE-AWG) concept" of Section 2 of the main manuscript, we assumed the extreme case $f_{\mathrm{shift}} = B$, i.e., $f_2 = f_1 + B$, that applies when generating a target waveform that fully exploits the bandwidth of the PE-AWG and its underlying hardware components. For a pure PAM signal without pulse shaping, this corresponds to generating the highest possible symbol rate f_{sym}: in this case $f_{\mathrm{sym}} = 4B$ such that the PE-AWG output waveform covers an electrical bandwidth $2B$. We chose this case in subsection "Photonic-electronic arbitrary-waveform generator (PE-AWG) concept" of Section 2 of the main manuscript to illustrate that quadrature multiplexing leads to a doubling of the analog bandwidth. The application of any practical pulse-shaping filter of non-zero roll-off, however, is associated with an excess bandwidth beyond $f_{\mathrm{sym}}/2$, i.e., beyond B in the most extreme case. Note that this roll-off and the associated excess bandwidth play an important role in digital timing recovery and therefore conservation of signal integrity in the band associated with the excess bandwidth is crucial for proper evaluation of the signal quality at the PE-AWG output.

In addition, smart choice of the digitally applied frequency shift f_{shift} further gives some flexibility to optimize the spectral position of the drive signals $u_{\mathrm{I}}(t)$ and $u_{\mathrm{Q}}(t)$ (with $\underline{u}(t) = u_{\mathrm{I}}(t) + \mathrm{j}\, u_{\mathrm{Q}}(t)$) with respect to the transfer function $\tilde{h}_{\mathrm{RF}}(f)$ of the transmitter electronics, i.e., the electronic AWG, the RF amplifiers, the RF cables, and the RF circuitry of the IQM. More specifically, proper choice of the spectral position of $u_{\mathrm{I}}(t)$ and $u_{\mathrm{Q}}(t)$ may allow to find an advantageous trade-off between the bandwidth limitations of the various system components, which is illustrated in more detail in Fig. S2. Figure S2(a) shows a sketch of the spectrum of the target waveform in grey, representing a PAM signal with a symbol rate of $f_{\mathrm{sym}} = 4B = 200\,\mathrm{GBd}$ and a roll-off parameter ρ=0.1, which has been chosen twice as high as in our experiments for better graphical illustration. The sketched waveform spectrum covers a spectral range from $-110\,\mathrm{GHz}$ to 110 GHz, including the 10% of excess bandwidth. The dotted lines refer to spectral components at negative frequencies that are simply the complex conjugate of their positive-frequency counterparts and thus carry only redundant information. These spectral components are eliminated by applying the Hilbert transform, see Section S2.2.1. The resulting analytic signal $\underline{A}_{s,n}$ whose spectrum is sketched in blue in Fig. S2(a) is then subjected to three exemplary frequency shifts $f_{\mathrm{shift}} \in \{f_{\mathrm{sym}}/4, f_{\mathrm{sym}}/4 \times (1+\rho), f_{\mathrm{sym}}/4 \times (1+2\rho)\}$, see Fig. S2(b), which shows the amplitude spectra $|\tilde{\underline{U}}_n(f)|$ of the frequency-shifted complex-valued signals $\underline{u}_n(t)$, where the tilde denotes variables in the Fourier space. For the exemplary symbol rate of 200 GBd and the roll-off parameter of ρ =0.1, the three frequency shifts amount to 50 GHz, 55 GHz, and 60 GHz. Note that we omit the addition of the RT for simplicity. In Fig. S2(c), we display the combined transfer function $\tilde{h}_{\mathrm{RF}}$ of the electronic AWG, the RF driver amplifiers, the RF cables, and the IQM circuitry (orange) along with the transfer function $\tilde{H}_{\mathrm{EO}}$ of the high-speed balanced photodetector (BPD1) (purple) that is used to finally translate the generated optical waveform back to the electrical domain. Note that the amplitude response of the electronic AWG, the RF driver amplifiers, and the IQM decays symmetrically for positive and negative frequency components, whereas the transfer function of BPD1 is centered about the left edge of the signal spectrum at $f = -f_{\mathrm{shift}}$. The overall transfer function is obtained by a multiplication of the two, $\tilde{h}_{\mathrm{tot}} = \tilde{h}_{\mathrm{RF}}\, \tilde{h}_{\mathrm{EO}}$, and is illustrated in red in Fig. S2(d). The shape of the overall transfer function $\tilde{h}_{\mathrm{tot}}$ depends on the frequency shift f_{shift}. For better comparison,

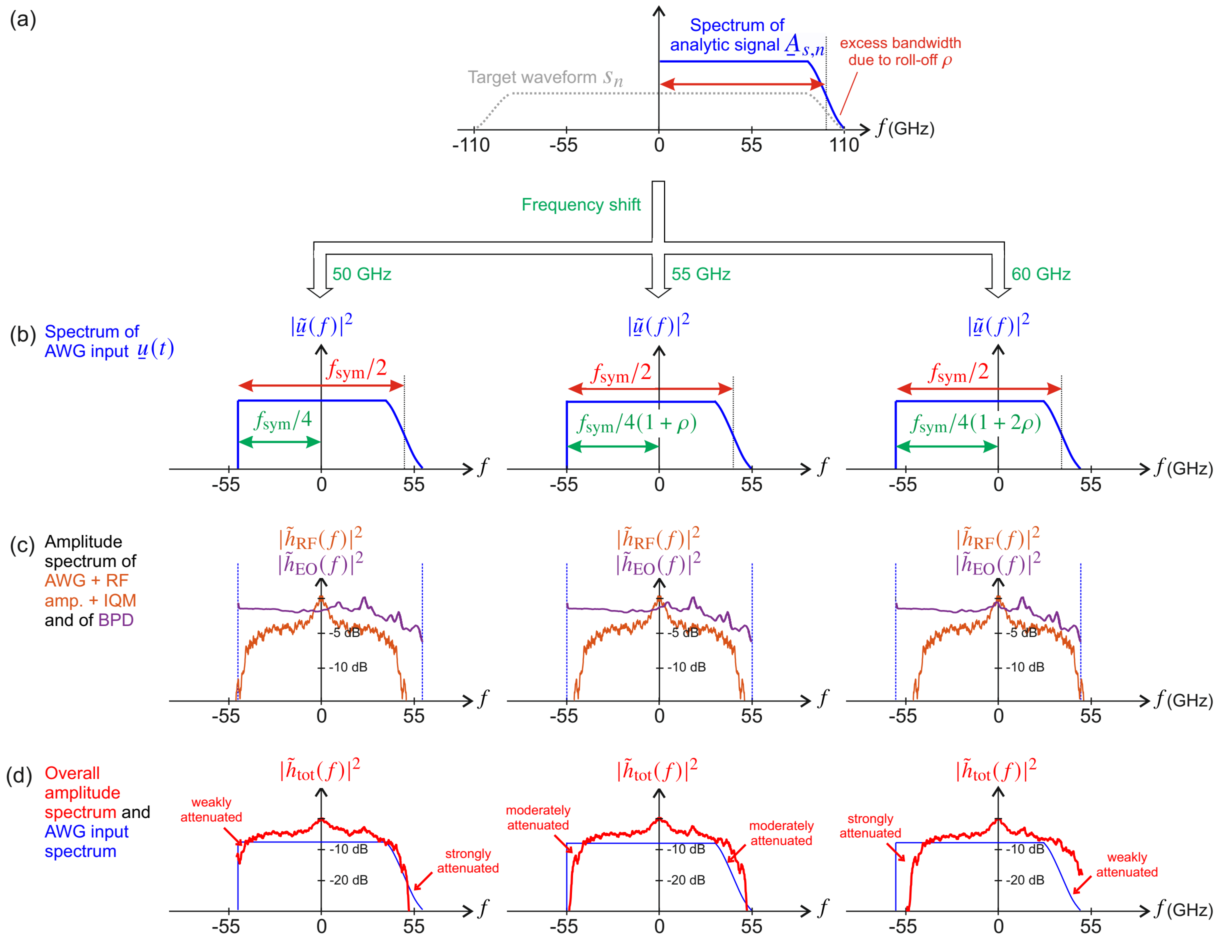

Fig. S2: Illustration of appropriate choice of the digitally applied frequency shift $f_{\mathrm{shift}} \leq B$, which defines the spectral distance between the LO tone at f_1 and the carrier of the optical IQ signal at f_2. **(a)** Sketch of the spectrum of the target waveform in grey, representing a PAM signal with a symbol rate of $f_{\mathrm{sym}} = 4B = 200\,\mathrm{GBd}$ and a roll-off parameter ρ=0.1. Smart choice of the frequency shift gives some flexibility to optimize the spectral position of the drive signals $u_{\mathrm{I}}(t)$ and $u_{\mathrm{Q}}(t)$, see **(b)**, with respect to the transfer function $\tilde{h}_{\mathrm{RF}}(f)$ of the transmitter electronics, i.e., the electronic AWG, the RF amplifiers, the RF cables, and the RF circuitry of the IQM, as sketched in **(c)**. In contrast, the transfer function $\tilde{h}_{\mathrm{EO}}$ of BPD1 as sketched in purple in (c) is always centered about the left edge of the signal spectrum at $f = -f_{\mathrm{shift}}$ so that the applied frequency shift affects the magnitude of the overall achieved frequency response $\tilde{h}_{\mathrm{tot}}(f)$, compare the red curves in **(d)**. The choice of f_{shift} hence allows to find an advantageous trade-off between the bandwidth limitations of the various system components. For our experiments, we use $f_{\mathrm{shift}} = f_s/4(1+\rho)$ as it balances the signal quality at the PE-AWG output over the full bandwidth range. Note that the frequency f_{shift} provided by the sine-wave generator shown in Fig. S1 needs to be adapted according to the digitally applied shift.

we also repeat the spectrum of the target waveform shown in Fig. S2(b) in Fig. S2(d). This illustration shows that for $f_{\mathrm{shift}} = f_{\mathrm{sym}}/4 = 50\,\mathrm{GHz}$ (left), the strongest attenuation occurs in the roll-off region of the waveform, i.e., between 45 GHz and 55 GHz. For $f_{\mathrm{shift}} = f_{\mathrm{sym}}/4(1+2\rho) = 60\,\mathrm{GHz}$ (right), in contrast, the frequency-dependent attenuation caused by various components mostly impairs the negative-frequency components from $-45\,\mathrm{GHz}$ to $-55\,\mathrm{GHz}$, whereas $f_{\mathrm{shift}} = f_{\mathrm{sym}}/4(1+\rho) = 55\,\mathrm{GHz}$ (center) is a compromise, for which the frequency-dependent suppression is in good approximation equally distributed between negative- and positive-frequency components. In our experiments, we use the latter setting, $f_{\mathrm{shift}} = f_{\mathrm{sym}}/4(1+\rho)$ to balance the signal quality at the PE-AWG output over the full bandwidth range. The LO tone is later added at the low-frequency edge of the optical IQ signal, i.e. the frequency shift provided by the sine-wave generator in the experimental setup, see Fig. S1, is adjusted accordingly.

S2.2.3 Digital post-processing

For evaluating the performance of the PE-AWG, we connect its output directly to a 100-GHz real-time oscilloscope (Keysight UXR1004A). The digital receive signal features an original length of 2 million samples (Sa), corresponding to a time duration of approximately 20 µs. It is then digitally re-sampled to 2 Sa/sym, before a feed-forward timing recovery is

used to compensate for the deviations between the receiver clock and the transmitter clock. The timing error is estimated with the algorithm proposed by Barton and Al-Jalili [14], which exploits the spectral redundancy of a pulse-shaped PAM signal caused by the excess bandwidth. The timing offset is then removed by applying a Lagrange interpolator in a Farrow structure. As a next step, we apply a receive filter, which is identical to the pulse-shaping filter described in Section S2.2.1. Neglecting any further signal impairments, these two filters form both a matched filter pair that maximizes the SNR and a Nyquist filter that guarantees zero inter-symbol interference (ISI) at the optimum sampling points. After down-sampling to 1 Sa/sym, an optional linear adaptive equalizer with L_{eq} coefficients may be used to suppress residual ISI that has not yet been eliminated by the predistortion of the drive signals $u_{\mathrm{I}}(t)$ and $u_{\mathrm{Q}}(t)$ to the IQM. In this case, the equalizer coefficients are updated blindly according to the Sato algorithm [15] in a first processing stage, which is followed by a second stage relying on the least-mean-squares (LMS) algorithm for a decision-directed update of the coefficients [16]. Finally, the demodulated receive signal is compared with the symbol pattern $\mathbf{x}$ for performance evaluation.

S2.2.4 Signal-to-noise-and-distortion ratio for PAM signals

For evaluating the performance of our PE-AWG, we use the signal-to-noise-and-distortion ratio $\mathrm{SNDR_{PAM}}$ estimated from received PAM symbols after digital post-processing[2]. The $\mathrm{SNDR_{PAM}}$ is a more suitable and reliable quality metric than the bit error ratio (BER) as it can be applied to signals for which no or only very few bit errors are found. More specifically, the $\mathrm{SNDR_{PAM}}$ is more sensitive to performance degradation than the BER in the sense that increasing distortions might already lead to a measurable $\mathrm{SNDR_{PAM}}$ decrease long before a change of the BER can be detected. Assuming data-aided reception, i.e., knowledge about the transmit pattern, the $\mathrm{SNDR_{PAM}}$ expressed in decibels (dB) is defined as

$$\mathrm{SNDR_{PAM}} = 10 \times \log\left(\frac{\overline{|U_{\mathrm{id}}|^2}}{\overline{|U_{\mathrm{meas}} - U_{\mathrm{id}}|^2}}\right). \tag{S.20}$$

In this relation, the symbols U_{id} and U_{meas} refer to the targeted (ideal, id) and the actually measured (meas) voltage amplitudes at the output of the PE-AWG, and the overbar denotes an average over many symbols with subscript $n = 1 \ldots N$. The quantity $\overline{|U_{\mathrm{id}}|^2} = \frac{1}{N}\sum_{n=1}^{N} |U_{\mathrm{id},n}|^2$ can thus be considered as the average power of N randomly transmitted symbols, whereas $\overline{|U_{\mathrm{meas}} - U_{\mathrm{id}}|^2} = \frac{1}{N}\sum_{n=1}^{N} |U_{\mathrm{meas},n} - U_{\mathrm{id},n}|^2$ represents the averaged power of the noise and distortions of the associated N received PAM symbols.

In Fig. 3(d) of the main manuscript, we use the $\mathrm{SNDR_{PAM}}$ of our PE-AWG as a metric to compare the performance of our approach to that of other signal-generation concepts that were used to generate PAM or quadrature amplitude modulation (QAM) signals in the associated literature. In case of PAM signals, we estimated the corresponding $\mathrm{SNDR_{PAM}}$ either directly from reported signal-to-noise ratios or similar metrics, or we used the reported Q factors or bit-error ratios (BER) to reconstruct the underlying SNDR. The same approach was applied for benchmarking with experiments, in which QAM signals were generated. For QAM signals, the so-called constellation SNR (CSNR) is a widely used metric [18–20], which can be directly related to the $\mathrm{SNDR_{PAM}}$ introduced above. Specifically, if the in-phase and the quadrature components of a QAM signal are generated by a pair of identical, but statistically independent waveform generators, then the CSNR of the resulting QAM signal is identical to the $\mathrm{SNDR_{PAM}}$ that each of the waveform generators would individually produce for an amplitude-modulated PAM signal having the same number of levels as each of the QAM components.

S2.3 Active optical phase stabilization

As discussed in subsection "Photonic-electronic arbitrary-waveform generator (PE-AWG) concept" of Section 2 of the main manuscript, active stabilization of the LO phase with respect to the signal is key for generation of arbitrary target waveforms with high fidelity. The phase control is achieved by a feedback loop as highlighted in turquoise in Fig. S1, which allows to establish an electro-optic phase-locked loop (PLL) as briefly discussed in subsection "Active optical-phase stabilization" of Section 2 of the main manuscript. In our proof-of-concept implementation, the PLL comprises a low-speed BPD (BPD2) that serves as an error detector, an analog or digital proportional-integral (PI) controller that forms the loop filter (LF), and an electrically controlled phase shifter (PS), that relies on mechanical stretching of optical fibers and that is used as the actuator. For superimposing the optical IQ signal and the LO tone, we use a 90° optical hybrid (OH), see subsection "Active optical-phase stabilization" of Section 2 of the main manuscript. While the in-phase (I) output ports of the OH, labeled by 'I$^+$' and 'I$^-$' in Fig. S1, are connected to a high-speed BPD (BPD1) to

[2] In our previous publication [17] we referred to this metric as the one-dimensional constellation SNR (CSNR). To avoid confusion with the CSNR for complex-valued signals, we decided for a re-naming.

generate the target waveform, the quadrature outputs ‘Q^+’ and ‘Q^-’ are used to tap monitoring signals to BPD2, which generates the error signal. Note that not necessarily 50% of the optical power need to be tapped to BPD2. The output of BPD2, $u_{\mathrm{BPD},2}(t)$, features an electrical bandwidth of 15 kHz, thus averaging over many transmitted symbols in our experiment. $u_{\mathrm{BPD},2}(t)$ is then proportional to $\sin(\Delta\phi(t))$ as derived in Eq. (S.19), Section S1.4, and can be used to feed either an analog PI controller ('Analog PI Contr.' in Fig. S1(a)) or a 15-kHz analog-to-digital converter (ADC) with a sampling rate of 1 MSa/s, which is followed by a digital PI controller ('Dig. PI Contr.' in Fig. S1(a)) implemented on a microprocessor. For the digital PI controller, the digital feedback signal generated at the output is converted to the analog domain by a 320-kHz DAC with a resolution of 12 bit. The voltage $u_{\mathrm{BPD},2}(t)$ already depends nonlinearly on the optical phase offset $\Delta\phi(t)$, see Eq. (S.19), and provides an s-shaped transfer characteristic, which inherently drives the phase to the desired operating point, characterized by an approximately linear relationship between the phase offset and the error signal. Note that the s-shaped characteristic might also include a DC offset, which may result from an imperfect balancing of the low-speed BPD and which needs to be subtracted before the signal is fed to the controller. The gain factors of the P and I element of the controller are heuristically optimized with the Ziegler-Nichols tuning method [21]. As a last step, the feedback signal is boosted by a voltage amplifier and finally applied to the piezo-driven PS which acts on the LO tone. As an alternative, the phase correction could as well be applied in the signal arm of the setup or directly in the corresponding DSP chain. The feedback loop is configured to minimize the phase offset $\Delta\phi(t)$ and converges to a phase difference of $0, 2\pi, 4\pi \dots$. Note that the interference of the LO and the RT, that generates the error signal, also appears at the high-speed output of the PE-AWG (i.e. BPD1), but with a cosine relation $C\cos(\Delta\phi(t))$, see Sections S1.3 and S1.4 for details. The active optical phase stabilization hence leads to a maximization of this term for $\Delta\phi(t) \in \{0, 2\pi, 4\pi \dots\}$, leading to an undesired DC component in the generated signal in our experiment. As a result, generation of target waveforms $s(t)$ with specific DC levels is difficult with our current PE-AWG implementation since any DC component of the target waveform will always interfere with the DC component that is exploited for phase control. Using pilot tones with small frequency offsets to the LO tone instead of the RT at the LO frequency could be an option to avoid such interference and render generation of specific DC levels possible. This approach might come at the price of distorting this very component of the target waveform, but the distortion might be kept small and might hence be tolerable in applications of practical interest. Note also that the synthesizer providing the drive signal to the MZM in Fig. S1 is time-synchronized with the AWG clock, thereby avoiding any deviation from the targeted frequency offset f_{shift} between the LO tone and the optical carrier, which would lead to a constantly increasing phase offset that could hardly be compensated by a piezo-based phase shifter with limited control range (55π). Future implementations of PE-AWG may be based on endless optical phase shifters as offered by a low-speed IQ modulator that can be even integrated onto the PIC as shown in Fig. 1 of the main manuscript. In our experiments, we equalize the fiber lengths in the signal and the LO arm of the optical setup to avoid additional variations of the phase difference $\Delta\phi(t)$ in case of frequency fluctuations or drift of the laser source. To this end, we insert patch cords with a total length of approximately 56 m in the LO path, see Fig. S1(a), dictated by the fiber lengths of the EDFAs in the signal paths. In a PIC, semiconductor optical amplifiers (SOAs) would be used instead, and matching of optical path lengths to µm precision does not pose a challenge. Note that, in the specific case of communication signals, the distortions induced by the optical phase drift can also be mitigated by adaptive linear equalizers [17] at the receiver side, which may additionally relax the complexity of the transmitter implementation, see Section S3.4.

S2.3.1 Characterization of optical phase fluctuations

To characterize the phase fluctuations in our optical setup, we first operate the system with open control loop and record the error signal $u_{\mathrm{BPD},2}(t)$ with a real-time oscilloscope over an observation period of 5 s as displayed in Fig. S3(a) in black (uncontr.). In Fig. S3(b), we show a zoom-in version of the first 20 ms of the trace shown in Subfigure (a). Since the optical phase evolves smoothly and the sampling period of the oscilloscope (sampling rate 200 kSa/s) is much shorter than the time constant of the phase fluctuations, we attribute the maxima and minima of $u_{\mathrm{BPD},2}(t)$ to extreme values of $\sin(\Delta\phi(t))$, i.e., to phase differences of $\Delta\phi(t) = \pm\frac{\pi}{2}, \pm\frac{3\pi}{2}, \pm\frac{5\pi}{2}, \dots$. In our recording, the changes by π take times of the order of ∼30 ms or more, but phase changes of a few degrees can happen much faster. In Figs. S3(a) and (b), we also plot the monitoring signal $u_{\mathrm{BPD},2}(t)$ recorded when the PI controller is activated (contr.). The digital implementation of the PI controller refers to the red curve, whereas the yellow curve represents the fully analog PI controller. We find that the slow phase drift is eliminated in both cases, but the optical phase evolution looks much less noisy for the analog PLL. Note that the closed-loop traces in Fig. S3(a) and (b) were recorded in the steady state of the system with the phase-control loop being fully effective and that they do not contain any initialization period. We have not exactly measured the initialization time of the control loop in our proof-of-concept setup, but it is in the order of a few milliseconds. Admittedly,

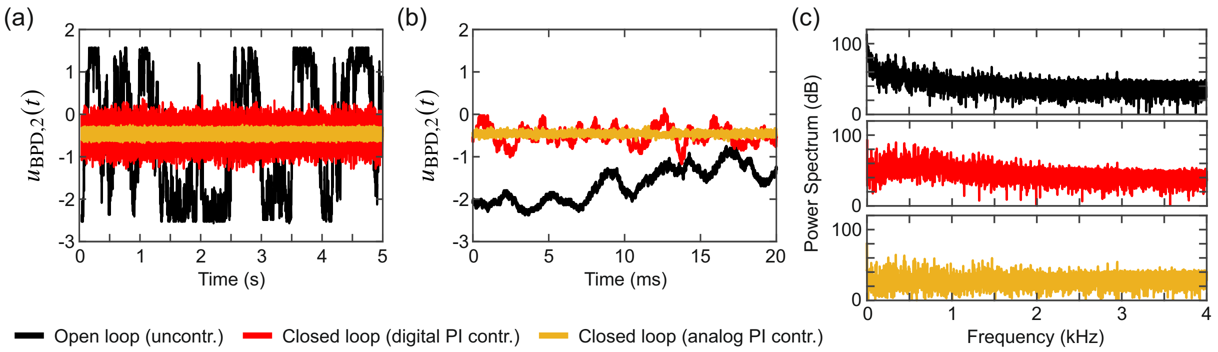

Fig. S3: Characterization of the active optical phase stabilization. **(a)** Error signal recorded with a low-speed oscilloscope at the output of BPD2 over a duration of 5 s. The black curve shows the evolution of the error signal in case of an open loop, i.e., a freely drifting optical phase. In contrast, the red and yellow curves are obtained when the phase stabilization is activated and a digital (red trace) or analog (orange trace) proportional-integral controller (PI contr.) is used, respectively. **(b)** Zoom-in of the first 20 ms of the waveform in (a). **(c)** Power spectrum corresponding to the waveforms shown in (a). For the digital PI controller, we observe a removal of the low-frequency phase drift but find that the signal is deteriorated by noise mainly covering a range from 0.25 kHz to 1 kHz. This effect is attributed to the latency of the digital controller implementation, which an be improved by using different controller hardware. The analog PI controller works more effectively and leads to a very flat spectrum.

the initialization time is very long compared to the symbol period of the generated high-bandwidth PAM waveforms (5 ps for 200 GBd) and compared to very short pulses or bursts that may be generated with the PE-AWG. A strategy to continuously maintain the locking even for very short target waveforms could be the permanent generation of a DC offset or a pilot tone that keeps the phase control loop active. The effectiveness of the phase control and the difference between the digital and the analog implementation can also be seen in the corresponding spectra depicted in Fig. S3(c). For the spectrum related to the digital PI controller (red), additional frequency components are clearly visible between 0.25 kHz and 1.5 kHz as compared to the flat yellow spectrum associated with the analog PI controller. We attribute this to the improved dynamics of the PLL in case of the analog PI controller. Specifically, while the bandwidths are similar for both controller implementations (digital: ∼8 kHz, analog: 10 kHz), the digital PI controller causes a significant latency of the order of 500 µs, whereas the delay is of the order of only ≤50 ns for the analog counterpart. We believe that the latency could be significantly reduced if implementing the digital version of the PI controller on a dedicated microcontroller such as a Raspberry Pi or a field-programmable gate array (FPGA). As pointed out in Section S3.4, while the analog PI controller results in a more stable $\mathrm{SNDR}_{\mathrm{PAM}}$ level, the noisy phase evolution associated with the digital PI controller does not translate into a substantial performance degradation, and decently stable electronic waveforms and SNR levels are achieved at the PE-AWG output even if the digital PI controller is employed. This may also be partly attributed to the timing-recovery algorithm that partially mitigates distortions induced by the imperfect phase control, see Section S3.4.3 for more details. In a fully-integrated version of the PE-AWG, we expect the optical phase drift to be even slower than in our lab setup with discrete components. We therefore believe that a micro-controller-driven optical phase adaptation is sufficiently fast for the active optical phase stabilization required in the PE-AWG.

S3 Characterization and performance benchmarking with broadband waveforms

This section provides an in-depth characterization of the PE-AWG that goes beyond the discussions in the main manuscript and that is based on broadband PAM target waveforms. In Section S3.1, the frequency dependence of noise, distortions, and the signal-to-noise-and-distortion ratio (SNDR) are analyzed, which allows to derive distinct SNDR penalties for quantifying a specific type of impairment such as linear distortions, nonlinear distortions, or noise. In Section S3.2 we go a step further and compare the power spectral densities of noise and distortions of the PE-AWG and the underlying electronic AWG (Keysight M8194A) in more detail. In both cases, we also consider noise introduced by the real-time oscilloscope used for analyzing the generated waveforms. We identify distinct hardware components and physical effects in our current PE-AWG setup as the root cause of the various distortions that come on top of those of the purely electronic AWG. Section S3.3 extends the performance benchmarking to a larger set of commercially available DACs and AWGs as well as to research-level and prototype-level waveform-generator concepts reported in the literature. Finally, Section S3.4 presents a statistical analysis of the PE-AWG performance for various waveforms and discusses specific applications scenarios in which the active optical phase control can be omitted.

S3.1 Frequency-dependent signal-to-noise-and-distortion ratio (SNDR)

In Fig. S4, we investigate the frequency-dependent impact of linear distortions, nonlinear distortions, and noise on the waveforms generated with our proof-of-concept PE-AWG, assuming different levels of predistortions applied during digital synthesis of the IQM drive signals. More specifically, we consider the case without any predistortion, the case of a predistortion that compensates for all linear signal impairments, and the (rather theoretical) case of a predistortion that undoes both linear and nonlinear impairments and leaves random noise as the only relevant detrimental effect. We further determine the frequency-dependent signal-to-noise-and-distortion ratio (SNDR) of the generated waveforms. For our analysis, we use 190-GBd[3] PAM4 data signals with a pulse shape having an RRC spectrum with roll-off factor of $\rho = 0.05$ as exemplary target waveforms. These waveforms are based on an N-element pseudo-random sequence, which was generated with the Mersenne-Twister algorithm [4] and which corresponds to an associated sequence $\mathbf{x}$ of PAM4 symbols. We record the associated PE-AWG output with an oscilloscope. The oscilloscope is not part of the device under test and additionally impairs the measured signal quality because of its limited effective number of bits (ENOB) that is comparable to that of the employed electronic AWG channels. To minimize the distortions caused by the oscilloscope, we set its full-scale voltage U_{FS} to 1.25× the peak-to-peak voltage at the PE-AWG output. The recorded waveforms are processed offline to extract the received PAM4 symbols $\mathbf{r}$. In the following, the term 'received PAM4 symbol' corresponds to the sampled value of the waveform in the center of the corresponding symbol slot, obtained after timing recovery, applying a receive filter with an RRC spectrum, resampling to one sample per symbol, and scaling of the amplitude to match the power of the symbol pattern $\mathbf{x}$ associated with the target waveform, see Section S2.2.3 for details on the DSP. Note that no adaptive equalization is used to extract the received PAM4 symbols. The received PAM4 symbols $\mathbf{r}$ are then used to calculate the symbol-by-symbol error

$$\mathbf{e}_{\mathrm{tot}} = \mathbf{r} - \mathbf{x}. \tag{S.21}$$

This error is then separated into a contribution by linear impairments and a contribution by nonlinear impairments and noise. To quantify the contribution by linear signal distortions, we estimate the L-element discrete-time impulse response $\hat{\mathbf{h}}_{\mathrm{PE-AWG}}$ of the PE-AWG system using a least-squares approach [6],

$$\hat{\mathbf{h}}_{\mathrm{PE-AWG}} = (\mathbf{X}^{\mathrm{T}}\mathbf{X})^{-1}\mathbf{X}^{\mathrm{T}}\mathbf{r}. \tag{S.22}$$

In this relation, $\mathbf{X}$ is a $N \times L$ circulant matrix generated from the symbol pattern $\mathbf{x}$ with a sliding window of L elements, and $(\mathbf{X}^{\mathrm{T}}\mathbf{X})^{-1}\mathbf{X}^{\mathrm{T}}$ is the generalized inverse (pseudo-inverse) of $\mathbf{X}$. Note that the estimated PE-AWG impulse responses $\hat{\mathbf{h}}_{\mathrm{PE-AWG}}$ contain the respective predistortion that was applied during digital synthesis of the IQM drive signals. Applying the estimated linear channel to the symbol pattern $\mathbf{x}$ associated with the target waveform results in the expected received symbols $\hat{\mathbf{r}}$ that contain only linear distortions,

$$\hat{\mathbf{r}} = \mathbf{X}\hat{\mathbf{h}}_{\mathrm{PE-AWG}}. \tag{S.23}$$

The linear distortions $\mathbf{e}_{\mathrm{lin}}$ can hence be found by subtracting the symbol pattern $\mathbf{x}$ from the actually received symbols $\hat{\mathbf{r}}$,

$$\mathbf{e}_{\mathrm{lin}} = \hat{\mathbf{r}} - \mathbf{x}. \tag{S.24}$$

We finally subtract the linear distortions from the overall distortions to obtain the nonlinear distortions and the noise as the remaining error,

$$\mathbf{e}_{\mathrm{nln}} = \mathbf{e}_{\mathrm{tot}} - \mathbf{e}_{\mathrm{lin}} = \mathbf{r} - \hat{\mathbf{r}}. \tag{S.25}$$

Next, we use Welch's method [22] to estimate the power spectral densities (PSD) of the symbol pattern $\mathbf{x}$, of the received symbols $\mathbf{r}$, of the linear distortions $\mathbf{e}_{\mathrm{lin}}$, of the nonlinear distortions and noise $\mathbf{e}_{\mathrm{nln}}$, as well as of the total error $\mathbf{e}_{\mathrm{tot}} = \mathbf{e}_{\mathrm{lin}} + \mathbf{e}_{\mathrm{nln}}$. The PSDs are indicated in terms of dBmHz^{-1} of electrical power per Hz of bandwidth. Finally, we calculate the frequency-dependent SNDR as the ratio of the PSDs of $\mathbf{x}$ and $\mathbf{e}_{\mathrm{tot}}$. The results are depicted in Fig. S4, which is organized in three columns: The first column consisting of Figs. S4(a), (b), and (c) refers to the case where no predistortion is applied to the digital waveforms fed to the AWG such that the frequency-dependent roll-offs of the

[3] This symbol rate is more representative for the PE-AWG performance than 200 GBd, as in the latter case, the performance is already impaired by the roll-off of our 100-GHz oscilloscope.

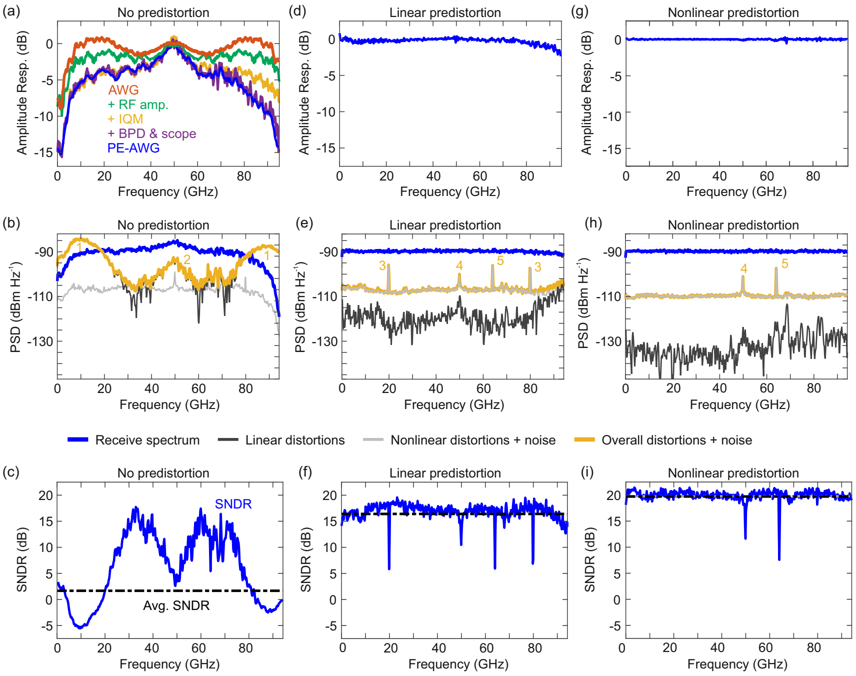

Fig. S4: Analysis of the frequency-dependent distortions and the performance of the PE-AWG using a 190-GBd PAM4 waveform as a test signal. **(a)** Estimated amplitude transfer function of the PE-AWG (blue trace) in absence of any predistortion, along with the measured accumulated amplitude transfer functions for various subsets of the PE-AWG hardware setup. The brown curve describes the individually measured response of the electronic AWG only, the green curve additionally includes the measured frequency-dependent losses of the subsequent RF amplifiers, the yellow curve additionally includes the distortions of the IQM, and the purple curve those of the BPD and the oscilloscope. In other words: The purple curve represents the expected transfer function of the overall system, obtained from the individual measurements of the various components, which coincides fairly well with the directly measured transfer function depicted in blue. **(b)** Estimated power spectral densities (PSD) of the generated waveform (blue) along with the associated overall distortions and noise (yellow) as obtained by comparing the PSD of the actually generated waveform as measured with the oscilloscope to the PSD of the ideal target waveform. The nonlinear distortions and noise (light grey) can be extracted by first estimating the linear system response from the measured signal and by comparing the correspondingly corrected waveform to the actually generated one. This also reveals the linear distortions (dark grey) as the deviation of the ideal target waveform from the one obtained after applying the linear system response. In the absence of any predistortion, linear impairments clearly limit the PE-AWG performance. **(c)** Estimated SNDR as a function of frequency, as obtained from relating the PSD of the generated waveform, blue trace in Subfigure (b), to the overall distortions and noise (yellow trace in (b)). The dashed line corresponds to the average SNDR, obtained by considering the full spectral power of the signal, noise, and distortions. **(d)** Estimated amplitude response of the PE-AWG (blue trace) when applying a linear predistortion scheme to the digital waveforms fed to the AWG. The resulting amplitude transfer function has less than 3 dB roll-off up to 95 GHz. **(e)** Estimated PSD of the generated waveform (blue) along with the associated overall distortions and noise (yellow), the nonlinear distortions and noise (light grey, coinciding well with the yellow curve), and the residual linear distortions (dark grey) in analogy to Subfigure (b). The linear predistortion reduces the PSD of the linear distortions such that nonlinear distortions and noise become the dominant impairments from DC to 80 GHz. Beyond 80 GHz, the linear distortions are not suppressed very well, which is a consequence of the specific design of the predistortion filters. We identify several tones that contribute to the overall distortions, namely clock tones of the underlying electronic AWG ('3'), the down-converted optical carrier that is not fully suppressed by the automatic bias control ('4'), and an oscilloscope clock tone ('5'). **(f)** Estimated SNDR as a function of frequency, again obtained from relating the PSD of the generated waveform to that of the overall distortions and noise. The linear predistortion improves the average SNDR to 16.4 dB (dashed line) and leads to a relatively weak frequency dependence of the SNDR except for the spectral positions of the spurious tones indicated in Subfigure (e), at which strong drops of the SNDR are visible. **(g)** Estimated amplitude response of the PE-AWG (blue trace) when applying an idealized nonlinear digital predistortion. The nonlinear predistortion relies on a pattern-dependent look-up table (PD-LUT) that compensates the deterministic error per symbol, so that only time-dependent deterministic errors remain and the system performance becomes noise-limited. It should hence be considered as an upper bound for the performance achievable with practical nonlinear predistortion schemes such as a Volterra-type pre-equalizers [10] or neural networks [23]. **(h)** Estimated PSD of the generated waveform (blue) along with the associated overall distortions and noise (yellow), the nonlinear distortions and noise (light grey, coinciding extremely well with the yellow curve), and the residual linear distortions (dark grey) in analogy to Subfigure (b). The PD-LUT compensates both nonlinear and residual linear distortions over the entire frequency range. Note however, that the down-converted optical carrier ('4') and the clock tone of the oscilloscope ('5') cannot be eliminated by predistortion as their amplitude and phase vary over time. **(i)** Estimated SNDR as a function of frequency, again obtained from relating the PSD of the generated signal to that of the overall distortions and noise. The PD-LUT improves the SNDR level to 19.7 dB. A tailored nonlinear predistortion might hence further improve the PE-AWG performance as compared to a purely linear predistortion.

PE-AWG hardware components remain fully uncompensated. The second column, Figs. S4(d), (e), and (f), shows the curves measured when using the linear predistortion scheme as discussed in Section S2.2.1, whereas the third column, Figs. S4(g), (h), and (i), is obtained from using a nonlinear predistorter that is explained later in this section. Each column displays the estimated magnitude of the one-sided amplitude transfer function $|\hat{\mathbf{H}}_{\mathrm{PE-AWG}}|$ in the first row along with the various above-mentioned PSDs in the second and the frequency-dependent SNDR in the third row.
Without any digital predistortion, first column, the PE-AWG output is characterized by a strong frequency-dependent attenuation for high modulation frequencies of the IQM used to generate the optical signal. This leads to a strong decay of the associated amplitude transfer function by approximately 15 dB close to DC and close to the highest generated frequency of approximately 95 GHz, see blue curve in Fig. S4(a). The spectral shape, which is approximately symmetrical with respect to the down-converted optical carrier frequency $f_{\mathrm{shift}} = 49.875$ GHz, is a direct consequence of quadrature multiplexing as introduced in subsection "Photonic-electronic arbitrary-waveform generator (PE-AWG) concept" of Section 2 of the main manuscript, for which any uncompensated bandwidth limitation of the electronic AWG, the RF amplifiers, or the IQM affects both high and low RF frequencies of the generated signal alike. The various accumulated contributions to these bandwidth limitations are indicated by different colors in Fig. S4(a). Specifically, we plot the measured amplitude transfer functions for various subsets of the PE-AWG hardware setup, where the brown curve refers to the amplitude response of the electronic AWG (M8194A) and shows a peaking at frequency components separated by 20 to 40 GHz from the down-converted optical carrier at $f_{\mathrm{shift}} = 49.875$ GHz and a strong decline of the response beyond that. The RF amplifiers introduce additional attenuation in the regions of the peaking such that a rather flat overall transfer function results, see green curve. The yellow curve further includes the frequency-dependent losses of the IQM, that amount to 3 dB at a 30 GHz distance from the down-converted optical carrier. In contrast to the transmitter electronics (AWG and RF amplifiers), the IQM features a response with a very smooth roll-off up to 50 GHz that can be easily compensated digitally without causing too much increase of the PAPR. Above RF frequencies of 60 GHz, the bandwidth limitation of the high-speed BPD comes into play, leading to the amplitude transfer function indicated by the purple curve. The attenuation introduced by the BPD increases with frequency and leads to a distortion that is spectrally asymmetric with respect to the down-converted optical carrier frequency $f_{\mathrm{shift}} = 49.875$ GHz. Note that the purple curve, which is obtained from the product of the individually measured transfer functions of the AWG, the RF amplifiers, the IQM, the BPD and the oscilloscope, coincides pretty well with the directly measured transfer function indicated in blue. In the absence of any digital predistortion, linear impairments clearly limit the PE-AWG performance. This can be seen in Fig. S4(b), where the yellow curve, representing the PSD of the overall error, pretty much follows the dark grey curve, associated with the linear impairments. The linear distortions directly translate into a low, partly negative and strongly frequency-dependent SNDR as depicted in Fig. S4(c). The average SNDR, obtained by considering the full spectral power of the generated waveform, as well as of the noise, and the distortions, is indicated by a dashed black line in Fig. S4(c). It is identical to the $\mathrm{SNDR}_{\mathrm{PAM}}$ measured from the received data and amounts to 1.7 dB.
Application of the two linear predistortion filters h_{RF} and h_{EO} as introduced in Section S2.2.1, second column in Fig. S4, results in an PE-AWG amplitude transfer function with less than 3 dB roll-off up to 95 GHz, see Fig. S4(d). Furthermore, the linear predistortion reduces the PSD of the linear distortions to a level below -110 dBm/Hz for frequencies up to 80 GHz, see Fig. S4(e), so that nonlinear distortions and noise become the dominant impairments in this spectral region. Beyond 80 GHz, the linear distortions are not suppressed very well – this is typical for minimum-mean-square error (MMSE) (pre-)equalizers, which find an optimum trade-off between distortions caused by bandwidth limitations along the analog signal path and distortions caused by too strong pre-amplification of high-frequency signal components and the resulting increase of the PAPR. In Fig. S4(e), we further identify several strong tones that contribute to the distortions and that have detrimental impact on the PE-AWG performance. Two tones ('3') separated by 30 GHz from the down-converted optical carrier at $f_{\mathrm{shift}} = 49.875$ GHz are caused by leakage of the 30-GHz clock of the electronic AWG. The down-converted residual optical carrier ('4') itself is another undesired distortion. An ideal automatic bias control settles exactly at the minimum transmission point of the IQM and should thereby completely suppress the carrier under the assumption that the IQM has a sufficient extinction ratio. The bias control used in our experiments, however, could not perfectly track the DC bias drift. Another prominent clock tone located at 64 GHz ('5') is added by the oscilloscope used to record the signal, however is not part of the actual PE-AWG output. Although all these tones contain only little power due to their narrow spectral width, they can significantly deteriorate the performance of the PE-AWG in a communication system because receiver-based linear adaptive equalizers tend to converge to a suboptimal solution in case of such distortions, either by trying to compensate it, which they cannot do, or by locking onto one of the tones. In our experiments we find that the interference of the target waveform with the residual down-converted optical carrier is the most crucial impairment among those and can provoke $\mathrm{SNDR}_{\mathrm{PAM}}$ degradations of more than 1 dB at $\mathrm{SNDR}_{\mathrm{PAM}}$ levels of approximately 15 to 20 dB as obtained in our experiments. Therefore, a high-quality bias control and a high

extinction ratio of the IQM are essential for high performance. Figure S4(f) indicates again the frequency-dependent as well as the average SNDR of the 190 GBd PAM4 signal. Overall, the linear predistortion improves the average SNDR at the PE-AWG output to 16.4 dB, dashed black line in Fig. S4(f), and leads to a relatively weak frequency dependence of the SNDR except for the spectral positions of the spurious tones indicated in Fig. S4(e), at which strong drops of the SNDR are visible. Note that the average SNDR can be further enhanced to 16.9 dB with an adaptive equalizer applied to the received signal (not shown in Fig. S4).

To quantify which fraction of the error $\mathbf{e}_{\mathrm{nln}}$ associated with the PSD displayed in light grey in Fig. S4(e) is related to deterministic effects and can be compensated by a proper calibration, we implement an idealized nonlinear digital predistortion that relies on a pattern-dependent look-up table (PD-LUT) that is applied in addition to the previously described linear predistortion. To this end, we evaluate the total error between the time-dependent symbol pattern $\mathbf{x}$ and the associated received symbols $\mathbf{r}$ recorded with an oscilloscope connected to the PE-AWG output over three pattern periods. We repeat the procedure for 50 recordings while keeping the PAM4 symbol pattern $\mathbf{x}$ unchanged, i.e., we fix the seed of the random number generator. We then align all 150 captured patterns in time and average such that the noise is removed and only the time-dependent deterministic error $\mathbf{e}_{\mathrm{nl}}$ remains. The deterministic error is then subtracted from the same symbol pattern $\mathbf{x}$ as to generate an ideally predistorted transmit signal. In essence, such a predistortion corresponds to a PD-LUT that accounts for a channel memory equivalent to the pattern length of $N = 100{,}000$ symbols. Undoubtedly, such a long LUT memory is unrealistic for any practical DSP implementation, and the coefficients of such a LUT cannot be determined as easily in a real-world system. The results obtained with the PD-LUT should hence be considered as an upper bound for the performance achievable with more advanced nonlinear predistortion schemes such as a Volterra-type pre-equalizers [10] or neural networks [23]. The results obtained from the PD-LUT-based predistortion are shown in the last column of Fig. S4. As seen in Fig. S4(h), the PD-LUT also compensates both nonlinear and residual linear distortions over the entire frequency range. This leads to a flat amplitude response, see Fig. S4(g), and a significantly enhanced SNDR level of 19.7 dB, see dashed black trace in Fig. S4(i), which is an improvement of > 3 dB compared to the linear predistortion. Specifically, the PSD of $\mathbf{e}_{\mathrm{tot}}$ consists of a noise floor at approximately -110 dBmHz^{-1} and of two remaining spurious tones ('4') and ('5'). We attribute the noise floor to noise from the transmitter electronics, i.e., the electronic AWG channels and the RF amplifiers, to acquisition noise from the oscilloscope (ADC), as well as to down-converted optical noise emitted by the EDFAs in the PE-AWG setup. The shot noise from the BPD is negligible in comparison to these noise contributions. Compared to the clock tones of the electronic AWG ('2') that are suppressed by the PD-LUT, the down-converted optical carrier ('4') is not eliminated as its amplitude varies over time due to a drifting IQM bias. Compensation could hence only be achieved with adaptive predistortion coefficients. Similarly, the clock tone of the oscilloscope ('5') has a random, time-variant phase offset with respect to the transmit signal and can hence not be generally removed by predistortion. Still, from the clear improvements obtained by the PD-LUT, we expect that a tailored nonlinear predistortion might improve the PE-AWG performance, even if limited to a realistic memory covering only a few symbol durations.

S3.2 Comparison of the PE-AWG and the electronic AWG

In the previous section, we investigated the contribution of linear effects, nonlinear effects, and noise to the overall error at the PE-AWG output. As a next step, we analyze the contributions of different system components to the overall distortions and noise found at the PE-AWG output. We further extend this analysis to a comparison of the waveform-generation performance of the PE-AWG to that of the underlying fully-electronic AWG (Keysight M8194A) to gain additional insights. As widely used quantitative benchmarks, we extract and compare the effective number of bits (ENOB) for both waveform generators, see Section S3.2.1 below. In addition, we determine the tolerance of the PE-AWG to IQ skew and IQ imbalance, see Section S3.2.2. In Fig. S5(a), we display in orange the PSD of a generated 100 GBd PAM4 waveform and of the overall noise and distortions obtained in an electrical back-to-back system where the electronic AWG (Keysight M8194A) is directly connected to a 100-GHz real-time oscilloscope by a coaxial cable. The PAM data signals are designed to have the same voltage swing as the IQM drive signals in our PE-AWG experiment. As in the case of Fig. S4(e), the frequency response of the AWG and the cable are mitigated by a linear MMSE predistortion filter, which is applied to the waveform before loading the latter into the memory of the electronic AWG. The contributions of noise and distortions are extracted using the same approach as described for Fig. S4, i.e., by rescaling and resampling the received symbols $\mathbf{r}$ associated with the generated waveform and by then subtracting them from the symbol pattern $\mathbf{x}$ associated with the target waveform. However, this time an additional adaptive linear equalizer is used to reduce the impact of the linear impairments that remain after MMSE predistortion, i.e., to remove the impairments that can straightforwardly be compensated by equalization. Considering the full spectral power of the generated waveform as well

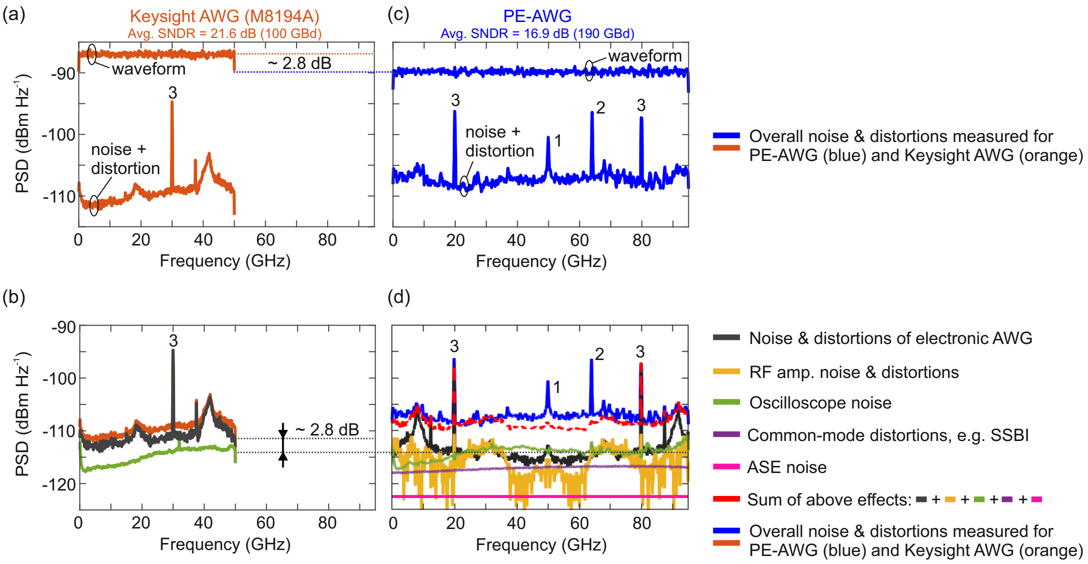

Fig. S5: Comparison of the performance of the PE-AWG to that of the underlying fully-electronic AWG (Keysight M8194A). We measure PAM4 signals with symbol rates of 100 GBd for the electronic AWG and of 190 GBd for the PE-AWG and compare the power spectral densities (PSD) of the generated waveforms and of the underlying distortions that remain after applying a linear predistortion scheme along with an adaptive linear equalizer. All waveforms are generated with the same RF power and captured with a 100-GHz real-time oscilloscope, where we use the same oscilloscope settings (bandwidth, sampling rate, and voltage scale) in both cases for better comparison. **(a)** PSD of a 100-GBd PAM4 waveform generated with the fully electronic AWG along with the estimated PSD of the associated overall noise and distortions. An average SNDR of 21.6 dB is obtained when relating the average PSD of the waveform to that of the overall noise and distortion. A strong clock tone of the AWG can be observed at 30 GHz and is labelled ('3') in analogy to Fig. S4. **(b)** Estimated PSD of the overall noise and distortions (orange trace) along with various subsets of distortions and noise for the fully-electronic AWG. The green curve corresponds to the oscilloscope noise measured from 0 to 50 GHz after passing through the DSP blocks to account for normalization effects and noise enhancement by the adaptive linear equalizer. Subtracting this noise from the orange trace, we obtain the PSD of the noise and distortions introduced by the electronic AWG, see dark grey curve. **(c)** PSD of a 190-GBd PAM4 waveform generated with the PE-AWG and PSD of the estimated overall noise and distortions. For the same RF power fed to the oscilloscope, the PSD of the overall signal reduces by approximately 2.8 dB as the signal is spread over approximately 1.9× the bandwidth. **(d)** Estimated PSD of the overall noise and distortions (blue trace) along with various subsets of distortions and noise for the PE-AWG. As seen, in our current implementation of the PE-AWG, several hardware imperfections along the signal path lead to additional performance degradation, and the bandwidth growth thus comes at the cost of an SNDR penalty of 4.7 dB. In analogy to Subfigure (b), the dark grey trace refers to the noise and distortions introduced by the electronic AWG channels - the spectral shape corresponds to a flipped version of the grey curve in Subfigure (b) between DC and 49.875 GHz and to a non-flipped version beyond 49.875 GHz, where the PSD level is reduced by 2.8 dB as the same RF power is again spread out over a 1.9× larger bandwidth. The yellow curve represents the PSD of the considerable noise and distortions added by the RF driver amplifiers used to boost the output signals of the electronic AWG channels to a power level that fits to the π-voltage of the IQ modulator. For the PE-AWG, we find an increased impact of the oscilloscope noise on the output waveform compared to the case of the purely electronic AWG, see green trace, as noise is accumulated over a larger bandwidth. While the amplified spontaneous emission (ASE) noise emitted by the optical amplifiers in the PE-AWG setup is of no relevant concern, see magenta trace and Inset Ⓓ of Fig. 2 in the main manuscript, distortions due to imperfect balancing of the high-speed BPD and the associated optical hybrid in terms of transmission and delay are noticeable, leading to common-mode distortions such as SSBI and degrading the SNDR further, see purple curve. The dashed red curve represents the expected PSD of the overall system, obtained by summing up the individual noise and distortion sources discussed so far. It coincides well with the directly measured PSD of the overall PE-AWG noise and distortions depicted in blue.

as of the noise and the distortions, we obtain an average SNDR of 21.6 dB. We verify this result by comparing it to the $\mathrm{SNDR_{PAM}}$ that we calculate in the time domain from the received PAM symbols of the test waveform according to Eq. S.20, leading to good agreement. In Fig. S5(b), we again plot the PSD of the overall noise and distortions (orange trace) along with various subsets of distortions and noise. Specifically, the green curve corresponds to the oscilloscope noise measured from 0 to 50 GHz by operating the oscilloscope without any input signal applied, i.e., the device input was simply terminated with a 50 Ω resistor. For a fair comparison, this measurement relies on the same oscilloscope settings (sampling rate, bandwidth, voltage scaling) as used as when evaluating the PE-AWG performance. To account for normalization effects and noise enhancement by the adaptive linear equalizer, the noise is further passed through all DSP blocks used for the PE-AWG performance evaluation. For a detailed model of the oscilloscope and a discussion of its noise characteristics, we refer to [24]. By subtracting the PSD of the oscilloscope noise (green trace) from the PSD of the total noise and distortions (orange trace), we obtain the PSD of the noise and distortions introduced by the electronic

AWG, see dark grey curve. Besides featuring a strong clock tone at 30 GHz, labeled '3' in Fig. S5(b), the PSD of the AWG noise and distortions increases with higher frequency, which we attribute to noise enhancement caused by the adaptive equalizer and to intrinsic frequency-dependent distortions of the electronic AWG itself arising, e.g., from its peaking amplifier. As a next step, we connect the PE-AWG output to the oscilloscope, generate a 190 GBd PAM4 waveform and again evaluate the PSDs of the generated broadband waveform as well as those of the associated overall noise and distortions, see blue curves in Fig. S5(c). For a fair comparison, the settings of the experiment using the AWG only, see previous paragraph, and the experimental performance evaluation of the PE-AWG described here are chosen such that the generated PAM4 waveforms have the same RF power. Moreover, the electronic AWG is operated at its maximum sampling rate of 120 GSa/s in both cases. Using in addition the same oscilloscope settings (bandwidth, sampling rate, voltage scale), we thus observe an approximately 2.8 dB ($10 \times \log(1.9)$ dB) lower PSD level for the generated PE-AWG waveform in comparison to the 100 GBd PAM4 waveform shown in Fig. S5(a), because the same RF power is spread over 190 GHz of bandwidth rather than over 100 GHz. Note that we decided to use a symbol rate of 190 GBd for the PE-AWG signaling experiment, even though this does not perfectly reflect the potential doubling of the symbol rate and bandwidth. However, at a symbol rate of 200 GBd, the measured signal quality would be additionally impaired by the strong roll-off of the frequency response of the 100-GHz oscilloscope, especially because each PAM waveform comes with an excess bandwidth due to the non-zero roll-off of the underlying root-raised cosine pulse spectra, see Section S2.2.2. This strong attenuation at frequencies beyond 100 GHz cannot be effectively undone by a linear predistortion or an adaptive linear equalizer, and we therefore decided to avoid the problem by using a slightly lower symbol rate.

An ideal PE-AWG relying on quadrature multiplexing of two electronic waveform generator channels would not introduce any additional noise and distortions compared to the underlying electronic generator as both the signal power as well as the noise and distortion power are effectively spread over a larger bandwidth while their ratio remains unchanged. Such an ideal PE-AWG would just come at the cost of additional hardware components and power consumption. Real-world implementations, however, lead to performance degradations due to several hardware imperfections along the photonic-electronic signal path, comprising optical noise, SSBI, or additional frequency-dependent loss. In Fig. S5(d), we again plot the PSD of the overall noise and distortions (blue trace) along with the PSD associated with certain parts of our signal chain. Using Welch's method, the power spectral densities are finally plotted from the time-domain traces. In analogy to Fig. S5(b), the dark grey trace now refers to the overall noise and distortion introduced by the electronic AWG channels. Its spectral shape corresponds to a flipped version of the dark grey curve from Subfigure (b) between DC and 49.875 GHz and a non-flipped version beyond 49.875 GHz - a result of the heterodyne down-conversion of the optical IQ signal with an LO tone located exactly at the lower-frequency edge of the optical spectrum. The PSD level for the noise and distortion is reduced by approximately 2.8 dB compared to that depicted in Fig. S5(b), since the same RF power is spread out over a bandwidth that is 1.9× larger as discussed above. The orange curve in Fig. S5(d) represents the PSD of the noise and distortion added by the RF driver amplifiers used to boost the output signals $u_\mathrm{I}(t)$ and $u_\mathrm{Q}(t)$ of the electronic AWG channels to a power level that is adapted to the π-voltage of the IQ modulator. The PSD of the RF amplifier noise and distortions is calculated by comparing the results obtained in an electrical back-to-back measurement comprising the electronic AWG, the RF driver amplifiers, and the oscilloscope with the previously mentioned experiment where the output of the electronic AWG was directly connected to the oscilloscope, see dark grey curve in Fig. S5(b). Note that the extracted noise and distortions are sent through the DSP chain to account for normalization effects and noise enhancement in both cases (with and without RF driver amplifiers) before the PSDs are calculated, compared, and unfolded around the residual optical carrier at a frequency of 49.875 GHz in Fig. S5(d). For the PE-AWG, we find an increased impact of the oscilloscope noise on the output waveform compared to the case of the purely electronic AWG, see green trace in Fig. S5(d), as the voltage scaling remains unchanged but noise is accumulated over a larger bandwidth. This implies that the measured SNDR is an underestimation of the true PE-AWG capabilities and that the SNDR penalty compared to the electronic AWG is overestimated. This is a problem common to all high-symbol-rate demonstrations reported in the literature as the required oscilloscope bandwidth scales with the symbol rate and the oscilloscope noise cannot be neglected for currently available high-speed oscilloscope models whose ENOB is similar to that of commercially-available AWG models [25, 26]. The PSD of the amplified spontaneous emission (ASE) noise from the optical amplifiers in the PE-AWG setup, see magenta trace, is estimated from an OSNR measurement, based on a measurement of the optical spectrum performed right before the BPD input, and by assuming a spectrally flat noise floor. Again, noise and distortions are sent through all relevant DSP blocks. More importantly, the imperfection of the high-speed BPD leads to unsuppressed common-mode terms such as SSBI that degrade the SNDR further, see purple curve in Fig. S5(d). The spectral shape and power of the SSBI was obtained by receiving the optical IQ signal associated with generation of the PAM4 target waveform with the high-speed BPD without adding the optical LO tone and subsequently sending the SSBI through all relevant DSP blocks. The dashed red curve represents the expected

Subsystem / effect	SNDR (M8194A)	SNDR (PE-AWG)
AWG	22.9 dB	22.9 dB
AWG + osc.	21.6 dB	21.2 dB
AWG + osc. + RF amp.		19.3 dB
AWG + osc. + RF amp. + SSBI		18.7 dB
AWG + osc. + RF amp. + SSBI + ASE		18.5 dB
AWG + osc. + RF amp. + SSBI + ASE + Misc.		16.9 dB

Table S1: Average SNDR estimated for the various noise and distortion contributions in signaling experiments performed with the electronic AWG (Keysight M8194A) only (second column) and with the associated PE-AWG (third column). Note that the estimated performance of the PE-AWG is subject to an underestimation as compared to that of the electronic AWG since oscilloscope (osc.) noise is accumulated over a larger bandwidth in case of the PE-AWG. The various rows of the table refer to different sections of the respective signal path. "osc." refers to the ultra-broadband real-time oscilloscope, "RF amp." refers to the RF amplifiers,, "SSBI" to the SSBI of the the imperfect BPD, "ASE" to the optical amplifiers that add amplified spontaneous emission (ASE) noise along the signal path of the PE-AWG, and "Misc." summarizes the contributions of a series of hardware components of the PE-AWG, which were not analyzed in further detail.

PSD of the overall system, when considering the individual noise and distortion sources discussed so far. It coincides relatively well with the directly measured PSD of the overall PE-AWG noise and distortion depicted in blue. The remaining difference between the blue and red trace can be attributed to a variety of physical effects such as presence of the imperfectly suppressed optical carrier, marked by ('1') in Fig. S5(d), ADC clock tones ('2'), imperfect equalizer convergence due to these tones and hence residual linear distortions, imperfect polarization adjustment at the input of the optical hybrid, which leads to reduced power of the single-polarization signal while the incident ASE is present for both polarizations and is hence unchanged, imperfect suppression of the ASE noise in the region of the image band at frequencies below the LO tone, which result from an imperfect filter shape of the POF [27], limited optical carrier-to-noise ratio (OCNR) of the frequency comb used for modulation and coherent down-conversion, small imbalances of amplitudes and phases of the in-phase (I) and quadrature (Q) components generated by the IQM, residual IQ skew between the IQM drive signals $u_{\mathrm{I}}(t)$ and $u_{\mathrm{Q}}(t)$, enhanced transmitter noise due to the increase of the PAPR when pre-compensating the frequency response of the electro-optic components (IQM and BPD), as well as a finite extinction ratios of the IQM. Among these effects, we observed the imperfectly suppressed optical carrier to be one of the more critical effects, which can cause SNDR degradations of more than 1 dB. A high-quality bias control and a high extinction ratio of the IQM are thus essential for the implementation of the PE-AWG. Considering the full spectral power of the generated waveform as well as the power of all the discussed noise and distortion contributions, we obtain an average SNDR of 16.9 dB for the 190-GBd PAM4 waveform, which corresponds to a penalty of 4.7 dB compared to the SNDR of 21.6 dB that was obtained for the 100-GBd waveform generated directly with the underlying fully-electronic AWG, see previous paragraph. By relating the power of the various noise and distortion contributions of the PE-AWG to the overall power of the generated waveform, we can calculate SNDR levels associated with these contributions, see Table S1, and then conclude on the SNDR penalty associated with certain physical effects and hardware components. Using the SNDR of 21.6 dB achieved for the 100-GBd waveform generated directly with the underlying fully-electronic AWG as a baseline, we find an overall penalty of 4.7 dB. Out of this penalty, 0.4 dB are caused by the additional oscilloscope noise that is unavoidably captured and that cannot be attributed to the signal source. A considerable portion of 1.9 dB is attributed to the RF driver amplifiers, which are known as a main source of nonlinear distortions in optical transceivers and for which similar penalties have been reported before [28]. The presence of nonlinear distortions in the RF amplifiers was also confirmed by investigations of our PE-AWG scheme via two-tone measurements, see Section S4 below. However, such distortions can be (partially) compensated by a nonlinear predistortion scheme as discussed in the previous section. Accounting for SSBI as an additional distortion reduces the SNDR by another 0.6 dB, which fits well to Fig. 3(c) of the main manuscript. ASE noise has a rather small impact on the performance. The remaining SNDR penalty of 1.6 dB is finally caused by a number of additional noise and distortion effects (Misc.) that lead to a deviation of the blue trace from the red trace in Fig. S5(d) as discussed in the previous paragraph.

S3.2.1 Effective number of bits (ENOB)

The effective number of bits (ENOB) is a metric that is frequently used to quantify the quality of waveform generation by a DAC or an AWG. It is defined via the SNDR for a full-scale sine wave as a test signal, $\mathrm{ENOB} = (\mathrm{SNDR_{dB}} - 1.76\,\mathrm{dB})/6.02$ [29]. Note that this test signal has a PAPR of 3 dB, which needs to be considered when estimating the ENOB from a

broadband waveform as test signal that usually has a higher PAPR,

$$\text{ENOB} = \frac{\text{SNDR}_{\text{dB}} - 4.77\text{dB} + \text{PAPR}_{\text{dB}}}{6.02}, \tag{S.26}$$

where PAPR_{dB} denotes the PAPR (in dB) of the test signal for which the SNDR was measured. In our measurements, the evaluated waveforms exhibited a PAPR of 10 dB in case of 100 GBd PAM4 generated with the fully-electronic AWG and of 9.5 dB for the 190 GBd PAM4 generated with the PE-AWG, respectively. Using Eq. (S.26), the 22.9 dB SNDR estimated for a waveform generated with the electronic AWG, see Table S1, correspond to an ENOB of 4.7, which agrees relatively well with the value reported in [2]. Note that this ENOB is slightly lower than the ENOB of 5.4 specified by the manufacturer [25], which refers to operating the AWG with differential outputs rather than using it in single-ended configuration as done here. For the PE-AWG, the SNDR level of 16.9 dB, see Table S1, still includes the oscilloscope noise. For a fair comparison with the fully-electronic AWG, we first calculate the SNDR obtained when subtracting the oscilloscope noise power from the PSD of noise and distortions, which amounts to 17.3 dB. Inserting the relevant numbers into Eq. (S.26) yields an ENOB of approximately 3.7 for the PE-AWG. Note, however, that this ENOB is only an estimation and might not be entirely accurate – in case of dominant nonlinear distortions, the measured distortions and hence the ENOB depends on the exact test signal, and Eq. (S.26) might not lead to consistent results for test signals with vastly different PAPR values. Still, we feel that the estimated ENOB gives a first orientation and represents already a quite decent result for a first proof-of-concept experiment, while there is considerable room for further improving the performance by overcoming or mitigating the various imperfections of our experimental setup.

S3.2.2 IQ skew and IQ imbalance

In the previous subsection, we have listed IQ skew, caused, e.g., by timing mismatch between the drive signals $u_\text{I}(t)$ and $u_\text{Q}(t)$, IQ gain imbalance, e.g., due to amplitude mismatch between the drive signals, and IQ phase imbalance of the IQM as potential impairments that can occur in the optical waveform-generation part of the PE-AWG. To quantify their impact on the overall PE-AWG performance, we deliberately added these effects and individually identified the associated 1-dB penalty point with respect to the SNDR_PAM measured for an optimized reference configuration. For the optimized reference, we minimized the IQ skew to below 0.1 ps, aligned the electrical powers of the IQM drive signals, and used a bias controller to guarantee a phase imbalance below $\pm 2^\circ$ [30]. For studying the impact of non-idealities, the IQ skew was added digitally, the IQ gain imbalance was induced by adjusting the electrical output power of the two electronic AWG channels, and the IQ phase imbalance was introduced by manual adjustment of the IQM bias. For a PAM4 waveform at 190 GBd, we find $\sim$1 ps, $\sim$2 dB, and $\sim 10^\circ$ as the 1-dB penalty points of the IQ skew, the IQ gain imbalance, and the IQ phase mismatch, which is in good agreement with the values reported in [31] for 4-level QAM signals. Note, however, that the requirements for IQ skew optimization depend on the symbol rate of the optical IQ waveform, which is only half of the symbol rate that is obtained at the PE-AWG output after heterodyne down-conversion.

S3.3 Performance comparison with competing schemes

We finally compare the results obtained with our PE-AWG to the performance of other waveform-generator concepts. In Fig. S6, we depict the SNDR_PAM as a function of the symbol rate obtained for various commercially available DACs and AWGs and for other research-type waveform generators reported in the literature and we compare these results to the signal quality achieved with our proof-of-concept PE-AWG. Note that these results were achieved after receiver-side adaptive equalization, similar to the scheme used in our work, see Section S2.2.1 for details. Note also that our definition of the SNDR_PAM is directly related to the constellation SNR (CSNR) for QAM signals and hence allows a direct comparison of PAM and QAM experiments, see Section S2.2.4 for details. In Fig. S6, we consider only electrical waveform generators and disregard optical arbitrary-waveform generation (OAWG) schemes [32] or optical time-division multiplexing (OTDM) schemes [33]. We further omit demonstrations exploiting sophisticated Faster-than-Nyquist (FTN) concepts that rely on dedicated coding schemes to boost the symbol rate for a given analogue bandwidth [34, 35]. Results obtained in electrical back-to-back experiments are represented by square markers, whereas circular markers refer to optical back-to-back configurations, for which the performance of the underlying electrical waveform generator can typically be expected to be 1-2 dB higher if RF amplifiers have been used to boost the electrical signal [28]. If data points are not labeled with a reference, the SNDR_PAM has been measured in our own experiments. The blue markers highlight the SNDR_PAM levels of the PE-AWG discussed in this manuscript. The SNDR_PAM measured for the underlying electronic AWG (Keysight M8194A) alone is depicted as a single red data point at a symbol rate of 100 GBd. Note that the evaluation of some waveform generator concepts included nonlinear equalization techniques [9, 36, 37], while others

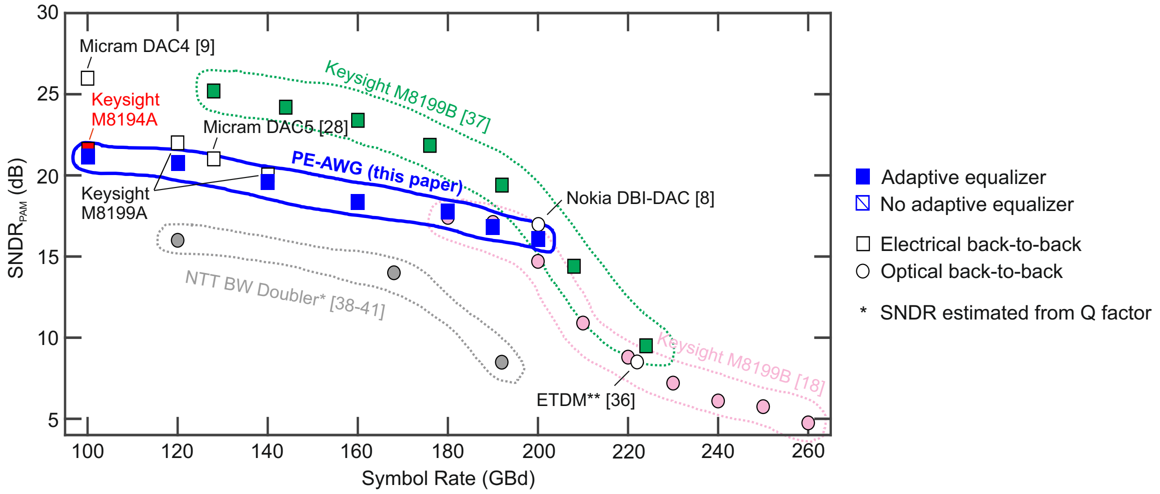

Fig. S6: SNDR$_{\mathrm{PAM}}$ as a function of the symbol rate measured for the proof-of-concept implementation of the proposed PE-AWG (blue markers) and comparison to commercially available DACs and AWGs and to results reported in the literature for other signal-generation concepts. The SNDR$_{\mathrm{PAM}}$ measured for the underlying electronic AWG (Keysight M8194A) alone is highlighted by a red data point at 100 GBd. Square markers refer to results measured in electrical back-to-back experiments, whereas circular markers denote optical back-to-back experiments, for which the performance of the underlying electrical waveform generator can be expected to be 1-2 dB higher [28]. If no reference for the data is given, the SNDR$_{\mathrm{PAM}}$ levels have been measured in our own laboratory. Note that for some waveform generator concepts, no SNDR$_{\mathrm{PAM}}$ data has been available. We therefore estimated the SNDR$_{\mathrm{PAM}}$ level from the Q factor (*) or the BER (**). We find that our proof-of-concept system can already compete with commercially available DACs and AWGs as well as with alternative bandwidth-enhancing concepts such as digital bandwidth interleaving (DBI) for example. Our concept is only outperformed by the recently released Keysight M8199B AWG [43], see green and pink markers [18, 37], which was not available at the time of our measurements.

relied on the use of linear filters only. Further note that for some waveform generator concepts, no SNDR$_{\mathrm{PAM}}$ data has been available. In these cases, we estimated the SNDR$_{\mathrm{PAM}}$ level from the Q factor (*) or from the BER (**), assuming that the performance has been limited by AWGN noise, which should give a reasonably reliable estimate. Overall, we find that, from 100 to 140 GBd, the performance of our first-generation PE-AWG is already on a par with several commercially available electronic waveform generators such as the underlying CMOS-based Keysight AWG model M8194A [2], the Micram DAC5 [28], or the Keysight M8199A. However, the PE-AWG reported herein features a much higher bandwidth and can generate symbol rates up to 200 GBd. At symbol rates beyond 140 GBd, the PE-AWG is only outperformed by Keysight's most recent AWG model M8199B, which relies on latest advancements in SiGe technology and which was not available during the time of our measurements. Still, although the PE-AWG is based on a more bandwidth-limited CMOS DAC and is built from discrete components, its signal quality at symbol rates of 180 GBd and above comes close to that of the Keysight M8199B. Moreover, the PE-AWG remains superior to most other bandwidth-enhancing concepts reported in the literature such as the bandwidth-doubling concepts proposed in [38–41] or active electrical time-division multiplexing by an InP 2:1 selector chip [36]. In comparison to the digital-bandwidth-interleaving DAC (DBI-DAC) [8, 19], we find a SNDR$_{\mathrm{PAM}}$ penalty of $\sim$ 1.5 dB at 200 GBd. However, we remark that the SNDR$_{\mathrm{PAM}}$ of the PE-AWG is an underestimation of the true SNDR$_{\mathrm{PAM}}$ at 200 GBd, as it is deteriorated by the roll-off of our 100-GHz real-time oscilloscope as opposed to the 113-GHz oscilloscope used in [8, 19, 42], see also discussion in Section S3.2 above. Keysight's most recent AWG model M8199B passively interleaves two SiGe DACs and features a nominal 3-dB bandwidth of 75 GHz as well as an ENOB of $\geq$ 5.0 up to 100 GHz [43]. As a consequence, the signal quality drops sharply for symbol rates beyond 200 GBd. Specifically, the M8199B system was used for generation of symbol rates up to 260 GBd, but SNDR$_{\mathrm{PAM}}$ levels were limited to rather low values of approximately 5 dB (see pink markers in Fig. S6, [18]). In a long-term perspective, a PE-AWG relying on quadrature multiplexing can double the bandwidth compared to the underlying electronic DAC as long as sufficiently broadband electro-optic components are available. In this context, the PE-AWG concept can benefit from advancements of high-bandwidth BPDs [44–48], where high optical input powers and high responsivities are key. Moreover, the proposed PE-AWG concept could profit from ongoing research activities in the field of broadband and compact electro-optic modulators as needed for future transceiver generations regardless of the electrical waveform generation [49–51].

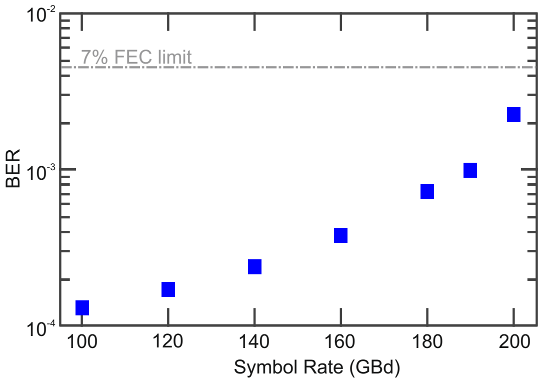

Fig. S7: BER as a function of the symbol rate measured in an electrical back-to-back experiment when generating electrical PAM4 waveforms with our PE-AWG. The output of the PE-AWG was directly connected to a 100-GHz oscilloscope. The dashed line refers to the 7% FEC limit. Note that a symbol-spaced adaptive linear equalizer was used in the receiver DSP to compensate for residual intersymbol interference (ISI) that has not yet been eliminated by the predistortion of the IQM drive signals. The displayed BER levels correspond to the SNDR levels represented by the filled blue square markers in Fig. S6.

S3.3.1 Bit-error ratio (BER) performance

As discussed in Section S2.2.4, we have decided to use the $\mathrm{SNDR}_{\mathrm{PAM}}$ as a metric for the waveform quality achieved at the PE-AWG output. In contrast to the bit error ratio (BER), the SNDR is a more reliable performance metric for waveforms for which no or only very few bit errors are found. For completeness, Fig. S7 shows the BER levels obtained for PAM4 signals with various symbol rates generated by the PE-AWG. The BER was extracted after receiver-side linear equalization in an electrical back-to-back system when generating PAM4 waveforms at various symbol rates. The displayed BER levels correspond to the SNDR levels represented by the filled blue square markers in Fig. S6 and in Fig. 3 of the main manuscript. We find that all BER levels are well below the threshold for hard-decision FEC with 7% overhead [52], see dashed line in Fig. S7 and Fig. S6, and clearly lower than the BER in excess of 4.5×10^{-3} measured in the optical transmission experiment as discussed in subsection "Application example: IM/DD fiber transmission experiment" of Section 2 of the main manuscript.

S3.4 Optical phase fluctuations - impact and compensation

In Section S2.3, we have described the implementation of our active optical phase stabilization and discussed the characterization of the phase fluctuations in our setup with and without activated control loop. However, we did not investigate the effect of the optical phase fluctuations and of the active phase stabilization on the PE-AWG performance yet, specifically their impact on the $\mathrm{SNDR}_{\mathrm{PAM}}$. The following sections are dedicated to this analysis. Section S3.4.1 focuses on the evaluation of $\mathrm{SNDR}_{\mathrm{PAM}}$ statistics and compares them for an activated phase control circuit and for a freely drifting optical phase. As pointed out in Section S3.4.2, the optical phase fluctuations $\Delta\phi(t)$ are equivalent to a time-dependent linear filtering of the waveform generated with the PE-AWG that only changes the phase relationship among the frequency components (all-pass filter). In the specific case of communication signals, this effect can be undone by a receiver-based linear adaptive equalizer that is often required anyway [17], see Section S3.4.3. Therefore, the active optical phase stabilization may be omitted in a communication system whereas it is imperative in high-fidelity arbitrary-waveform generation.

S3.4.1 Performance stability

As discussed in Section S2.3, optical phase drift is a slow process with time constants in the order of milliseconds, which corresponds to frequency components of 1 kHz or less, see Fig. S3(c). High-speed real-time oscilloscopes, however, are frequently used to record a limited data set for offline processing and performance evaluation. The latest Keysight oscilloscope used in our experiments (Keysight UXR1004A) features a memory depth of 200 MPts at a sampling rate of 256 GSa/s in its standard configuration [53] without special memory options, which restricts recording times to less than one millisecond. As a result, a high signal quality shown for a single waveform is no evidence that the underlying waveform generation concept works in general, since it might just happen to be reported for a randomly "correct" phase relation. To account for the randomness of the phase fluctuations in an adequate way and to validate our active optical phase stabilization concept, we therefore evaluate $\mathrm{SNDR}_{\mathrm{PAM}}$ statistics at the PE-AWG output for a multitude of PAM4 signals, taken at various times and with various symbol rates. Comparable results were observed for PAM2 and PAM8

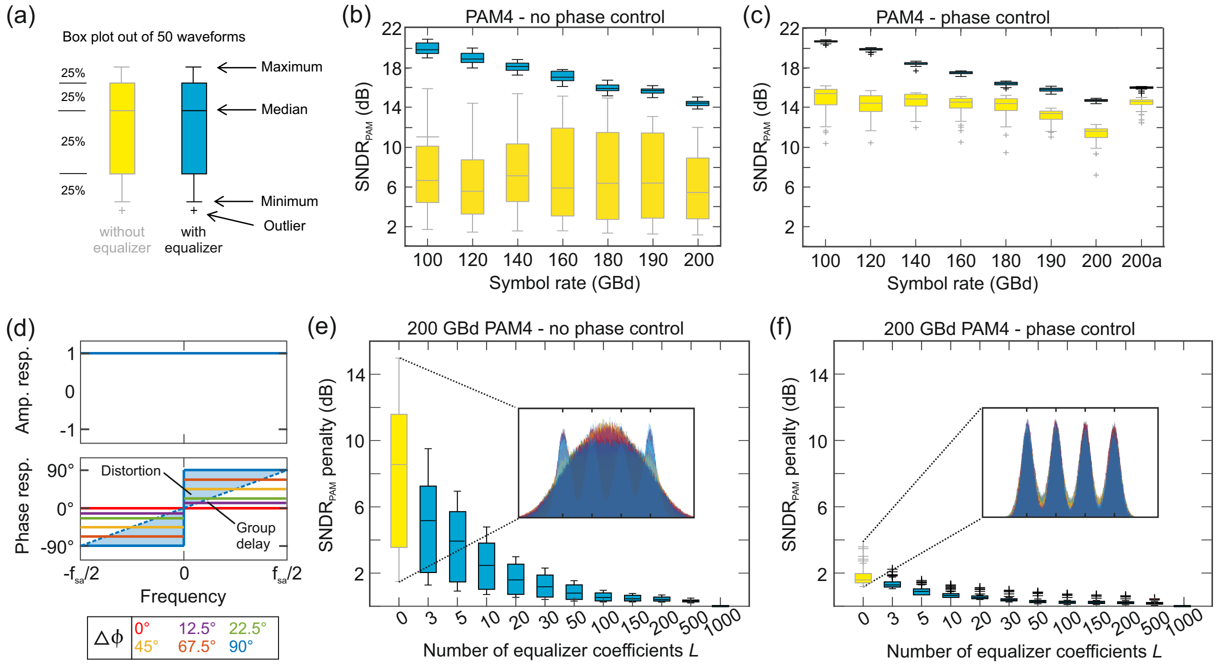

Fig. S8: Analysis of the impact of the optical phase fluctuations and of the active phase stabilization on the PE-AWG performance quantified by the $\mathrm{SNDR_{PAM}}$. **(a)** Explanation of the box plots as used in Subfigures (b), (c), (e), and (f) representing the $\mathrm{SNDR_{PAM}}$ statistics for 50 independent receive waveforms, which were recorded at intervals of several seconds. The horizontal line in the middle of each box represents the median of the measured $\mathrm{SNDR_{PAM}}$ values while the boundaries of the box correspond to the lower and the upper quartile and the whiskers at the top and the bottom of the box indicate the maximum and the minimum value of the data set, excluding some individual outliers that are identified based on the ratio of the respective distance to the box and the corresponding box height [54] and that are indicated as crosses. **(b)** $\mathrm{SNDR_{PAM}}$ statistics obtained without active phase stabilization, i.e., for a freely drifting optical phase as a function of the generated symbol rate. Box plots with yellow filling refer to the case where no receiver-based adaptive linear equalizer is applied, whereas such an equalizer with $L_{\mathrm{eq}} = 100$ coefficients is applied for the box plots with blue filling. The adaptive equalizer can mitigate the phase-induced distortions and substantially reduces the $\mathrm{SNDR_{PAM}}$ variatons. **(c)** $\mathrm{SNDR_{PAM}}$ statistics obtained with activated phase control as a function of the generated symbol rate. At a symbol rate of 200 GBd, the active phase control leads to a remarkable decrease of the $\mathrm{SNDR_{PAM}}$ variations from 13 dB, see Subfigure (b), to less than 4 dB when a digital PI controller is used and to about 2 dB including outliers for an analog PI controller ('200a'), see yellow box and associated whiskers at the very right of the subfigure. A combination of the active phase stabilization with an adaptive linear equalizer can improve the SNDR stability even further. **(d)** Effect of the optical phase fluctuations $\Delta\phi(t)$ on the waveform generated by the PE-AWG. The phase fluctuations are equivalent to a time-dependent linear filtering of the waveform generated with the PE-AWG that only changes the phase relationship among the frequency components (all-pass filter). The phase response of the all-pass filter can be divided into two contributions: a linear phase modulation over frequency, that changes the group delay at the BPD output and that is indicated by a dashed blue line, and an additional distortion that predominantly affects low-frequency components and vanishes at the Nyquist frequency $\pm\frac{f_{\mathrm{sa}}}{2}$, and that is indicated by shaded blue regions. In the specific case of communication signals, this all-pass filtering can be undone by a receiver-based linear adaptive equalizer. **(e)** Dependence of the $\mathrm{SNDR_{PAM}}$ penalty on the number L_{eq} of applied equalizer coefficients for a 200-GBd PAM4 waveform and a freely drifting optical phase. The SNDR penalty expectedly reduces with increasing L_{eq}. The inset shows the superposition of all 50 PAM4 histograms if no equalizer is used, with each histogram displayed in a different color. The optical phase fluctuations distort the histogram and the four peaks associated with the four PAM levels are only visible for a few recordings with coincidentally small phase offset $\Delta\phi$, whereas in most cases the histogram is deteriorated to the point of being unrecognizable as that of a PAM4 signal. **(f)** Dependence of the $\mathrm{SNDR_{PAM}}$ penalty on the number of applied equalizer coefficients L_{eq} for a 200-GBd PAM waveform and an activated phase control based on an analog PI controller. While the $\mathrm{SNDR_{PAM}}$ variations are already small for $L_{\mathrm{eq}} = 0$ and only slightly improved for an increasing number of equalizer coefficients L_{eq}, the overall $\mathrm{SNDR_{PAM}}$ penalty reduces with increasing L_{eq} as the equalizer can compensate residual linear distortions that have not been considered in the pre-distortion and that are unrelated to the optical phase drift. Even in absence of linear equalization, the histogram is clearly recognizable as that of a PAM4 signal.

signals, but are not discussed here. Specifically, for each experimental setting, we evaluate the $\mathrm{SNDR_{PAM}}$ for 50 receive waveforms recorded at intervals of several seconds, each with a recording length of $\sim$4 µs. The statistics of the resulting $\mathrm{SNDR_{PAM}}$ are displayed as box plots in Fig. S8. For a better interpretation of these box plots, we depict an exemplary one in Fig. S8(a) and label the essential elements. The horizontal line in the middle of each box represents the median of the $\mathrm{SNDR_{PAM}}$ and is used as a common baseline for all evaluations. The boundaries of the box correspond to the lower and the upper quartile, i.e. the median of the lower and the upper half of the $\mathrm{SNDR_{PAM}}$ data set. The whiskers at the top and the bottom of the box indicate the maximum and the minimum value of the data set, excluding some individual

outliers that are identified based on the ratio of the respective distance to the box and the corresponding box height [54] and that are indicated as crosses, see, e.g., Fig. S8(c). In Figs. S8(b) and (c), we compare the SNDR statistics for an uncontrolled optical phase and for an activated phase control based on a digital PI controller, respectively. For a symbol rate of 200 GBd, we also substitute the digital PI controller by a superior analog model, indicated by the data point '200a' in Fig. S8(c), see Section S2.3.1 for details of the phase controllers. Box plots with yellow filling correspond to the $\mathrm{SNDR}_{\mathrm{PAM}}$ statistics obtained when not using any adaptive linear equalizer in the receiver DSP, whereas the boxes with blue filling represent the case of an adaptive linear equalizer with $L_{\mathrm{eq}} = 100$ coefficients. The active phase control helps in increasing the stability of the PE-AWG performance quite remarkably: While the $\mathrm{SNDR}_{\mathrm{PAM}}$ evaluated without equalizer and without phase control varies by up to 13 dB, see yellow boxes and associated whiskers in Fig. S8(b), an activated phase control can reduce the variation to less than 4 dB if we neglect a few outliers that are caused by a reset of either the fiber stretcher or the PI controller during the recording time, see yellow boxes and associated whiskers in Fig. S8(c). Such resets become necessary from time to time because of the limited total phase shift of the fiber stretcher and the limited range of both ADC and DAC that are implemented together with the digital PI controller. With an endless phase shifter [55] such outliers are not expected to occur. Note that the activated optical phase control mainly improves the average $\mathrm{SNDR}_{\mathrm{PAM}}$ level and reduces the $\mathrm{SNDR}_{\mathrm{PAM}}$ variations, while the best $\mathrm{SNDR}_{\mathrm{PAM}}$ achieved per symbol rate is similar with and without phase control. This is no surprise because the evaluation of a large number of recordings will with a high probability include some waveforms with a coincidentally small phase offset $\Delta\phi$ even if the phase control is not activated. Further note that the maximum $\mathrm{SNDR}_{\mathrm{PAM}}$ levels shown for each symbol rate in Fig. S8(c) are not identical to those in Fig. S6 because the statistics were evaluated at an earlier point in time when the PE-AWG system was not fully optimized yet, while the data points depicted in Fig. S6 were measured with the fully-optimized system. The only exception is the data obtained with the analog PI controller, see right-most box plots in Fig. S8(c) ('200a'), as those were measured for a fully-optimized system as well. When substituting the digital PI controller by the analog model, we observe that the $\mathrm{SNDR}_{\mathrm{PAM}}$ fluctuations at 200 GBd decrease to about 2 dB including outliers. This is a remarkable performance stability and makes us believe that our active phase stabilization is well suited for high-fidelity photonic-electronic waveform generators, especially when implemented as a photonic integrated circuit, in which optical path lengths are short and hence optical phase fluctuations are less prominent. Moreover, Figs. S8(b) and (c) reveal that, in case of communication signals, an adaptive equalizer can cause a tremendous improvement of the $\mathrm{SNDR}_{\mathrm{PAM}}$ stability even if no optical phase control is applied - to a level on par with that achieved with the analog PI controller. Still, a combination of the active optical phase stabilization and the adaptive linear equalizer yields better stability and seems to be the preferred solution. In this case, the $\mathrm{SNDR}_{\mathrm{PAM}}$ does not vary by more than 0.5 dB at a symbol rate of 200 GBd.

S3.4.2 All-pass filter characteristics

This section provides an explanation why the receiver-based linear equalizer can cope with the distortions caused by the temporal phase drift. Under the assumption that the relative phase $\Delta\phi(t)$ between the optical signal and the LO tone does not change during the recording, we can treat $\cos(\Delta\phi(t))$ and $\sin(\Delta\phi(t))$ as constants. For symbol rates from 100 GBd to 200 GBd, the above assumption that $\cos(\Delta\phi(t))$ and $\sin(\Delta\phi(t))$ are constants is justified even when recording millions of symbols as the optical phase offset $\Delta\phi(t)$ drifts only over time scales of milliseconds and hence changes slowly compared to the recording length. In this case, the waveform generated at the PE-AWG output can be written as

$$i_{\mathrm{BPD},1} \propto s(t)\cos(\Delta\phi) - \mathcal{H}\{s(t)\}\sin(\Delta\phi), \tag{S.27}$$

where H denotes the Hilbert transform, see Eq. (S.12). Note that we again omitted the reference tone (RT) u_{RT} for simplicity. Making use of the fact that the Hilbert transform corresponds to a multiplication with a transfer function $\tilde{H}_{\mathcal{H}}(f) = -\mathrm{j}\,\mathrm{sgn}(f)$ in the Fourier domain [56], the Fourier transform of the recorded photocurrent can be written as

$$\tilde{I}_{\mathrm{BPD},1}(f) = \tilde{S}(f)\cos(\Delta\phi) + \mathrm{j}\,\mathrm{sgn}(f)\tilde{S}(f)\sin(\Delta\phi), \tag{S.28}$$

$$= \begin{cases} \tilde{S}(f)\big(\cos(\Delta\phi) - \mathrm{j}\sin(\Delta\phi)\big) & \text{for } f < 0 \\ \tilde{S}(f)\big(\cos(\Delta\phi) + \mathrm{j}\sin(\Delta\phi)\big) & \text{for } f > 0 \end{cases} \tag{S.29}$$

$$= \begin{cases} \tilde{S}(f)\exp(-\mathrm{j}\Delta\phi) & \text{for } f < 0 \\ \tilde{S}(f)\exp(\mathrm{j}\Delta\phi) & \text{for } f > 0 \end{cases}. \tag{S.30}$$

Note that $\mathrm{sgn}(f)$ is a generalized function which cannot be described by a specific value at $f = 0$, and the same applies to $\tilde{I}_{\mathrm{BPD},1}(f)$. As can be seen, the phase response of the PE-AWG is distorted by $\Delta\phi$ at positive frequencies and by $-\Delta\phi$ at negative frequencies, see Fig. S8(d), whereas the amplitude response does not change. This can be interpreted by a filtering of the output of the PE-AWG by a linear all-pass, which does not result in any loss of energy (fading) and which is a deterministic effect that can be compensated by an appropriately designed equalizer, see Section S3.4.3 for details. As illustrated in Fig. S8(d), we can split the phase response of this all-pass filter into two parts: a linear increase of the spectral phase, that corresponds to a group delay of $i_{\mathrm{BPD},1}$ and that is indicated by the dashed blue line in Fig. S8(d), and an additional distortion that predominantly affects low-frequency components and that vanishes at the Nyquist frequency $\pm\frac{f_{\mathrm{sa}}}{2}$ where f_{sa} denotes the sampling frequency, see shaded blue region in Fig. S8(d). Note that the group delay is always eliminated by the timing-recovery algorithm applied in our receiver DSP even if no adaptive linear equalizer is used. This means that the SNDR statistics shown in Fig. S8(b) and (e) do not reflect the additional random group delay caused by the optical phase changes.

S3.4.3 Necessity of the active optical phase stabilization

In principle, the linear all-pass filtering induced by the optical phase fluctuations as discussed in the previous section can be fully undone by an appropriately designed digital linear equalizer. Specifically, this equalizer is as well an all-pass filter and exhibits the frequency response

$$\tilde{H}_{\mathrm{eq,ideal}}(f) = \begin{cases} \exp(\mathrm{j}\Delta\phi) & f < 0 \\ \exp(-\mathrm{j}\Delta\phi) & f > 0. \end{cases} \tag{S.31}$$

Yet, as the optical phase $\Delta\phi(t)$ drifts over time, its exact realization within a given observation time (i.e. oscilloscope recording) is random and so is the associated distortion of the target waveform. Therefore, the optical phase drift needs to be tracked continuously in a real-time system, which requires an adaptive equalizer that continuously adapts its coefficients according to the optical phase fluctuations to approximate the frequency response in Eq. (S.31). Such a receiver-side compensation can be realized in communication systems, where only the end-to-end performance matters and the signal quality at the transmitter interface is of no particular interest. In high-speed optical transmission systems, tailored DSP chips including adaptive linear equalizers are state of the art and are anyway needed to mitigate the effects of bandwidth limitations and polarization mode dispersion (PMD) on the receive signal. The compensation of the phase-drift-induced distortions would then be only one additional task to be performed by the already existing equalizer, thereby reducing the hardware complexity of the PE-AWG. Still, two conditions need to be fulfilled to make such an adaptive equalizer an attractive alternative or supplement to implementing an optical phase control in an optical transmission system. First, the equalizer's adaptation rate needs to be fast enough to not loose phase lock. In the CMOS-based DSP circuits of modern high-speed optical transceivers, the DSP algorithms are parallelized and run at a clock rate much lower than the symbol rate. The equalizer coefficients are updated only once per clock cycle. Assuming a typical clock rate of 500 MHz [57], the equalizer still adapts 5000 times per ms, which should be good enough to track the phase evolution that occurs tyically on ms time scales. The fact that optical polarization rotates at a similar speed [58] and that commercially used equalizers can compensate PMD supports this notion. Second, the number of equalizer coefficients required to approximate the frequency response in Eq. (S.31) should be sufficiently small as to keep power consumption moderate and to avoid a re-design of the equalizer or the overall DSP engine. To address this aspect, we analyze the $\mathrm{SNDR}_{\mathrm{PAM}}$ penalty as a function of the number of equalizer coefficients L_{eq} for the exemplary case of 200-GBd PAM4 signals, see Fig. S8(e). The best $\mathrm{SNDR}_{\mathrm{PAM}}$ obtained for $L_{\mathrm{eq}} = 500$ is used as the benchmark to which the penalties refer. In analogy to Section S3.4.1, we contrast the scenario of an uncontrolled optical phase drift, Fig. S8(e), with the case of an actively stabilized phase, this time relying in an analog PI controller, see Figs. S8(f). Yellow boxes again refer to the case where no adaptive equalizer is applied ($L_{\mathrm{eq}} = 0$), and the insets in Figs. S8(e) and (f) show a superposition of the associated PAM4 histograms, where each histogram is displayed in a different color. As also illustrated in Fig. 1(b) of the main manuscript, the optical phase fluctuations distort the histogram and the four peaks associated with the four PAM levels are only recovered for a few recordings with coincidentally small phase offset $\Delta\phi$. However, when the phase control is activated, the four peaks are clearly distinguishable in all histograms. The $\mathrm{SNDR}_{\mathrm{PAM}}$ penalty measured without phase control decreases strongly with an increasing number of equalizer coefficients. If the number L_{eq} of taps exceeds 100, the variation of the $\mathrm{SNDR}_{\mathrm{PAM}}$ penalty may even fall below the SNDR penalty variation obtained for the case with optical phase control, but without equalizer - this can be observed by comparing the height of boxes with blue filling in Fig. S8(e) to the height of the box with yellow filling in Fig. S8(f). It is noteworthy that while the SNDR penalty variations are

comparable for the two cases, the absolute SNDR_{PAM} penalty achieved without phase control and $L_{\text{eq}} = 100$ equalizer coefficients is well below that observed with the optical phase control and $L_{\text{eq}} = 0$. We attribute this to the compensation of residual linear distortions by the equalizer that have not been compensated by the applied pre-distortion scheme and that are unrelated to the optical phase drift. This notion is supported by the SNDR_{PAM} penalty reduction observed when increasing the number of equalizer coefficients L_{eq} for an activated phase stabilization, see Fig. S8(f). Overall, the measurements confirm that even in an optical setup with discrete components and fiber lengths of tens of meters in each arm, the equalizer can very well eliminate distortions induced by the optical phase fluctuations. Towards a high number of equalizer coefficients, the SNDR_{PAM} penalties and penalty variations decrease towards zero for both scenarios, with a slight SNDR_{PAM} and stability advantage remaining when using an active phase control. We conclude that the best performance and performance stability is achieved when combining an active phase stabilization and a digital equalizer. The active optical phase stabilization also reduces the required number of equalizer coefficients. As an example, the optical PLL combined with an equalizer with $L_{\text{eq}} = 20$ coefficients performs better than an equalizer with $L_{\text{eq}} = 50$ coefficients (including outliers) or $L_{\text{eq}} = 150$ coefficients (neglecting outliers), respectively, that is not supported by an optical phase control. The root cause of the increased number of outliers in that case is a matter of ongoing research.

S4 Two-tone measurements

To demonstrate the capability of the PE-AWG to generate waveforms other than the truly arbitrary broadband PAM signals discussed before, we conduct two-tone experiments, where two monochromatic tones are generated by the resulting spectrum with all linear and nonlinear distortions are analyzed. This method is particularly well suited to reveal presence and strength of nonlinear distortions, which lead to mixing products and higher harmonics of the two generated test tones, thus giving a more direct insight into the signal quality. We start our experiments by using the PE-AWG to generate two target tones at frequencies f_{T1} and f_{T2} with a frequency offset $\Delta f_T = f_{T2} - f_{T1} = 1\,\text{GHz}$. These frequencies f_{T1} and f_{T2} are swept from 4 GHz and 5 GHz to 98 GHz and 99 GHz, respectively, while keeping the frequency offset Δf_T constant. As a second parameter, we tune the output power of the underlying electrical AWG from $-16\,\text{dBm}$ (100 mV_{pp}) to $-6.5\,\text{dBm}$ (300 mV_{pp}), which leads to different RF drive powers at the IQM inputs and reveals the various (nonlinear) distortions. Note that we use fixed-gain IQM drive amplifiers, such that an increase of the AWG output voltage directly translates into an increase of the drive voltage coupled to the IQM as long as the devices do not saturate. For each measurement, we capture the signals at the PE-AWG output with a 100-GHz real-time oscilloscope and evaluate the power spectral density (PSD) over the full frequency range from DC to 100 GHz. An exemplary PSD is shown in Fig. S9(a) for the case f_{T1} =66 GHz and f_{T2} =67 GHz. Besides the two generated tones (Tone 1, Tone 2; red and orange markers), several distinct distortions are visible in the PSD: The residual optical carrier (light green marker), signal-signal-beat interference (SSBI, dark green marker), a 30-GHz clock tone of the AWG as well as its subharmonics and mixing products (yellow markers), second- and third-order harmonics of the two tones in the IQM drive signals (brown and light blue markers), as well as third-order mixing products $2f_{T1} - f_{T2}$ and $2f_{T2} - f_{T1}$ (magenta markers) caused by nonlinearities of the RF driver amplifiers and the subsequent IQM, mixing products of the residual optical carrier and the two target tones (purple markers), and the undesired IQ image of the two target tones (blue markers). Additional distortions such as higher-order mixing products and their IQ images are represented by grey markers. As a reference, we also plot the PSD obtained when connecting the output of one of the IQM drive amplifiers directly to the real-time oscilloscope, see Fig. S9(b), indicating that quite a few of the observed distortions are already present in the IQM drive signals. Note that the horizontal axis of Fig. S9(b) refers to the frequency components of the IQM drive signals that are modulated onto the optical carrier at frequency f_2. In contrast to that, the frequencies found at the PE-AWG, Fig. S9(a), result from a down-conversion with an optical LO tone at $f_1 = f_2 - f_{\text{shift}}$. We have thus shifted the spectrum in Fig. S9(b) by $f_{\text{shift}} = f_2 - f_1 = 50\,\text{GHz}$ for better comparison of the various distortions shown Fig. S9(a) and Fig. S9(b). To quantify the impact of distortions and noise on the overall performance of the PE-AWG, we calculate the signal-to-noise-and-distortion ratio (SNDR) and the associated signal-to-distortion ratio (SDR) and signal-to-noise ratio (SNR) as performance metrics

$$\text{SNDR} = \frac{P_{\text{s}}}{P_{\text{dist}} + P_{\text{n}}}, \tag{S.32}$$

$$\text{SNR} = \frac{P_{\text{s}}}{P_{\text{n}}}, \tag{S.33}$$

$$\text{SDR} = \frac{P_{\text{s}}}{P_{\text{dist}}}, \tag{S.34}$$

$$\text{where} \frac{1}{\text{SNDR}} = \frac{1}{\text{SNR}} + \frac{1}{\text{SDR}}. \tag{S.35}$$

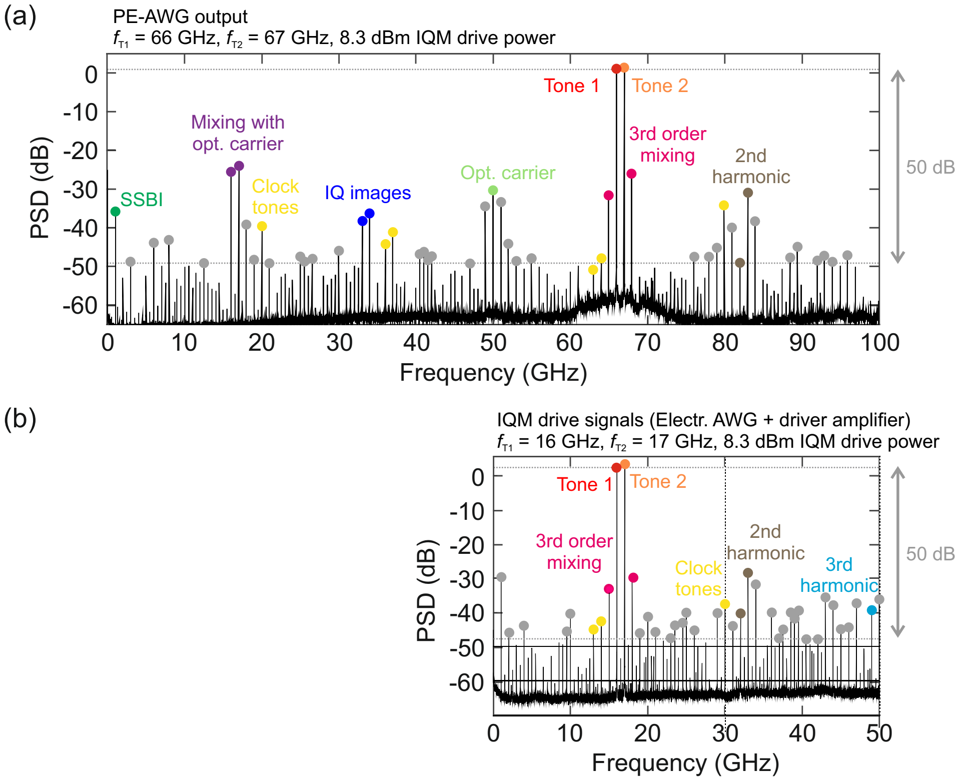

Fig. S9: Power spectral densities (PSDs) obtained when generating two target tones at frequencies f_{T1} =66 GHz and f_{T2} =67 GHz. Besides the two generated tones (red and orange markers), the PSD reveals several distinct distortions: Residual optical carrier (light green marker), signal-signal-beat interference (SSBI, dark green marker), 30-GHz clock tone of the electronic AWG and associated mixing products (yellow markers), harmonics of the two target tones (brown and light blue markers), third-order mixing products caused by nonlinearities of the RF driver amplifiers and the subsequent IQM (magenta markers), mixing products of the residual optical carrier and the two target tones (purple markers), and IQ images of the two target tones (blue markers). Note that the terms '2nd harmonic' and '3rd harmonic' correspond to the respective harmonics generated in the IQM drive electronics, see Subfigure (b). Additional discrete tones that are less than 50 dB below the level P_{T1} of the tone at frequency f_{T1} are represented by light grey markers. **(a)** PSD of the PE-AWG output. **(b)** PSD of the output of one of the IQM drive amplifiers acting as a reference and an indication of what distortions are introduced by the electronics of the PE-AWG system. Note that the horizontal axis of Subfigure (b) refers to the frequency components of the IQM drive signals that are modulated onto the optical carrier at frequency f_2. In contrast to that, the frequencies found at the PE-AWG, Subfigure (a), result from a down-conversion with an optical LO tone at $f_1 = f_2 - f_{\text{shift}}$. We have thus shifted the spectrum in Subfigure (b) by $f_{\text{shift}} = f_2 - f_1$ =50 GHz for better comparison of the various distortions shown in Subfigures (a) and (b).

In these relations, the signal power $P_{\text{s}} = P_{T1} + P_{T2}$ is the sum of the powers P_{T1} and P_{T2} of the two tones marked by an orange and a red marker in Fig. S9(a), respectively. The power of the distortions P_{dist} is defined as the total power of all discrete tones that are less than 50 dB below the level P_{T1} of the tone at frequency f_{T1} – these tones are indicated by markers in Fig. S9(a) and Fig. S9(b). The noise power P_{n} is finally the remaining power in the signal, having an overall power of P_{tot},

$$P_{\text{n}} = P_{\text{tot}} - P_{\text{s}} - P_{\text{dist}} \tag{S.36}$$

Note that the differentiation between noise and distortions via the 50 dB ratio is somewhat arbitrary, but still helps in understanding the impact of nonlinearities on the overall SNDR. In a next step of our analysis, we investigate the evolution of the SNR, the SDR, and the resulting SNDR with increasing output power of the electrical AWG, blue, cyan, and red traces, respectively, in Fig. S10. The first two rows of Fig. S10 display the SNR, the SDR, and the SNDR at the PE-AWG output as a function of the electrical power used to drive each of the IQM arms ('IQM drive power') for different frequencies f_{T1} of the first target tone. As a reference, we measure the same metrics for the IQM drive signals found at the output of the respective amplifiers, see third row of Fig. S10 ('Electrical AWG + IQM drive amplifier'), as well as for the AWG output signals that were generated by the electrical AWG and fed to the inputs of the IQM drive amplifiers, see fourth row of Fig. S10 ('Electrical AWG'). Within the first and the second row, we have aligned the

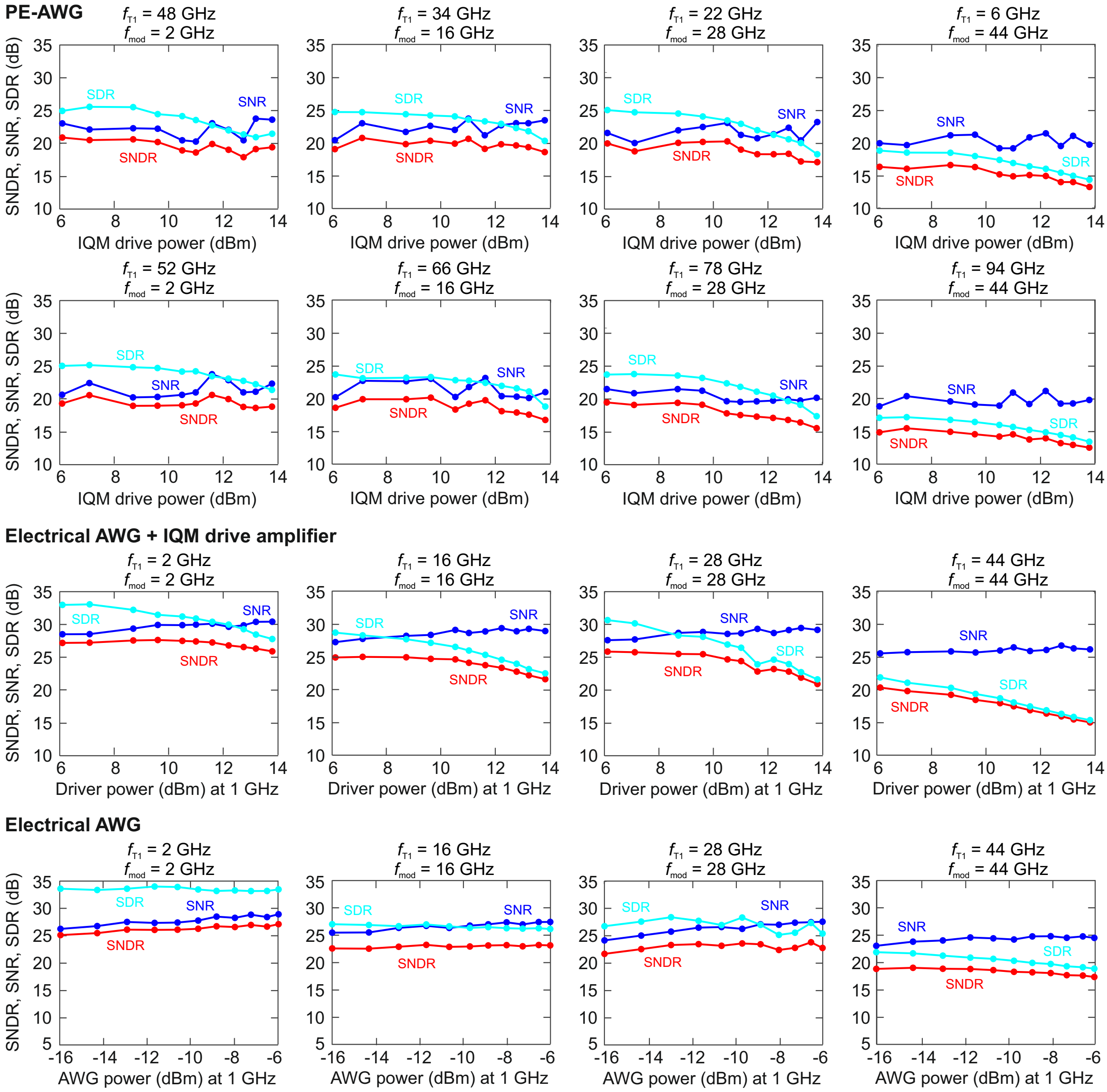

Fig. S10: Signal-to-noise ratio (SNR, blue traces), signal-to-distortion ratio (SDR, cyan traces) and the resulting signal-to-noise-and-distortion ratio (SNDR, red traces) displayed as a function of the electrical power for various carefully selected frequencies as estimated from the two-tone measurements. The first two rows illustrate the performance of the PE-AWG ('PE-AWG'), the third row corresponds to the IQM drive signals ('Electrical AWG + IQM drive amplifier'), and the fourth row corresponds to the signals that are generated by the underlying electrical AWG and fed to the IQM drive amplifiers ('Electrical AWG'). Within the first and the second row, we have aligned the various measurements such that those belonging to f_{T1} and $2f_{\text{shift}} - f_{T1}$ align with the corresponding measurement of the electrical AWG with or without IQM drive amplifier, operated at a modulation frequency $f_{\text{mod}} = |f_{T1} - f_{\text{shift}}|$. The horizontal axis refers to the power of the IQM drive signals for the first three rows, and to the electrical input power of the IQM drive amplifiers for the fourth one. We observe that the PE-AWG performance is limited by distortions (SDR, cyan) in case of high modulation frequencies f_{mod} and by noise (SNR, blue) at low modulation frequencies. By comparing the first two rows with the third row, we find that the IQM drive amplifiers represent the main source of nonlinear distortions to the entire PE-AWG system.

various measurements such that those belonging to f_{T1} and $2f_{\text{shift}} - f_{T1}$ align with the corresponding measurement of the electrical AWG operated at a modulation frequency $f_{\text{mod}} = |f_{T1} - f_{\text{shift}}|$ with or without IQM drive amplifier. We observe that the PE-AWG performance is limited by distortions (SDR, cyan) in case of high modulation frequencies f_{mod} and by noise (SNR, blue) at low modulation frequencies. The SNR of the PE-AWG largely lies between 20 dB and 25 dB for reasonable electrical power levels, which suggests that the SNDR of a broadband target waveform can be improved to this range if all distortions are compensated. This result matches well with the SNDR_{PAM} of 21.6 dB that

we have achieved for a 190-GBd PAM4 waveform when applying an idealized nonlinear predistortion that essentially suppresses all nonlinear distortions, see Section S3.1 for details. For the IQM drive signals ('Electrical AWG + IQM drive amplifier'), we find that nonlinear distortions (SDR, cyan) dominate the signal quality at high modulation frequencies f_{mod} (16 GHz or higher in our plots). This behavior is particularly pronounced at $f_{\mathrm{mod}} = 44\,\mathrm{GHz}$, which we attribute to the fact that the transfer function of the drive amplifiers exhibits a slight resonance-like peak at this frequency, which might lead to additional nonlinear distortions. As expected, the SDR decreases with increasing IQM drive power because the IQM drive amplifiers go into saturation and the IQM nonlinearity becomes more pronounced. As the very same evolution is found in the PE-AWG performance, see subfigures for $f_{T1} = 6\,\mathrm{GHz}$ or 94 GHz as an example, we conclude that the nonlinear distortions are a main limitation for the PE-AWG performance at high modulation frequencies f_{mod}. Finally, we evaluate the contribution of the electrical AWG to the mentioned distortions, see last row of Fig. S10. We find that the SNDR of the electrical AWG is dominated by distortions at high modulation frequencies as well, which is presumably caused by an internal peaking amplifier. Still, the IQM drive amplifiers represent the main source of nonlinear distortions to the entire PE-AWG system. This confirms our notion that a replacement or omission of the IQM drive amplifiers may lead to a significant performance improvement, see subsection "Further improvements, integration, and use cases of PE-AWG systems" of Section 3 of the main manuscript.

S5 Potential performance improvements

The experiments discussed in this paper represent a first proof-of-concept demonstration and clearly leave room for further improving the quality of the generated waveforms. Specifically, besides ensuring that the measured signal quality is not limited by the performance of the underlying oscilloscope, the results discussed in Section S3.1 and Section S3.2 let us believe that the performance of the PE-AWG output can be further improved, e.g., by the following adaptations:

- Replacement of the used electronic AWG (Keysight M8194A) by another device with a lower and smoother frequency-dependent roll-off. Specifically, using the latest commercial device (Keysight M8199B) would allow to use less aggressive predistortion and thereby reduce the PAPR and the noise level of the generated IQM drive signals considerably.
- The same applies to the IQM - the currently used devices have a bandwidth of 30 GHz - and could be replaced by more broadband modulators, e.g., based on thin-film lithium niobate [59, 60].
- Substitution of the deployed RF driver amplifiers by more linear ones and/or use of a more efficient modulator technology [49–51] that does not require any RF driver amplifier at all should reduce nonlinear distortions.
- Application of a tailored digital nonlinear predistortion scheme to reduce residual nonlinear distortions from the transmitter electronics and driver amplifiers.
- Regarding photonic-electronic down-conversion, a reduction of the optical path length mismatch between the OH and the high-speed BPD (BPD1) inputs by photonic integration could mitigate SSBI at lower LOSPR levels. This would allow for increasing the optical signal power incident on the BPD, leading to waveform generation with higher electrical output power without impairing the signal quality.
- The programmable optical filter (POF) in front of the BPD could be configured to partially undo the frequency-dependent attenuation of the preceding hardware components in the analog domain as in [18], thereby allowing for a less pronounced digital predistortion and an effectively reduced PAPR and accordingly increased SNDR of the modulator drive signals.
- Replacement of the used electronic AWG (Keysight M8194A) by a higher-bandwidth electronic AWG such as the latest Keysight model (M8199B) and replacement of the high-speed BPD by a more broadband one such as [44] would allow generating symbol rates beyond 200 GBd.

References

[1] R.-J. Essiambre, G. Kramer, P. Winzer, G. J. Foschini, and B. Goebel, “Capacity limits of optical fiber networks,” J. Lightw. Technol. **28**(4), 62-701 (2010).

[2] A. Matsushita, M. Nakamura, S. Yamamoto, F. Hamaoka, and Y. Kisaka, ”41-Tbps C-Band WDM Transmission With 10-bps/Hz Spectral Efficiency Using 1-Tbps/λ Signals,” J. Lightwave Technol., **38**(11), 2905-2911 (2020).

[3] P. Runge, G. Zhou, F. Ganzer, S. Keyvaninia, S. Mutschall, A. Seeger, R. Klötzer, S. Wünsch, and G. Ropers, ”100 GHz Balanced Photodetector Module,” Proc. Conference on Lasers and Electro-Optics (CLEO), paper STu3B.4 (2018).

[4] M. Matsumoto and T. Nishimuram, ”Mersenne Twister. A 623-Dimensionally Equidistributed Uniform Pseudorandom Number Generator,” ACM Trans. Model. Comput. Simul. **8**(1) (1998).

[5] C. Füllner, M. M. H. Adib, S. Wolf, J. N. Kemal, W. Freude, C. Koos, and S. Randel, ”Complexity Analysis of the Kramers-Kronig Receiver,” J. Lightwave Technol. **37**(17), 4295-4307 (2019).

[6] S. Randel, S. Corteselli, P. J. Winzer, A. Adamiecki, A. Gnauck, S. Chandrasekhar, A. Bielik, L. Altenhain, T. Ellermeyer, U. Dümler, H. Langenhagen, and R. Schmid, ”Generation of a Digitally Shaped 55-GBd 64-QAM Single-Carrier Signal Using Novel High-Speed DACs,” Proc. Optical Fiber Comm. Conf. (OFC), paper M2A.3 (2014).

[7] A. H. Sayed, *Fundamentals of Adaptive Filtering*, Wiley (2003).

[8] X. Chen, S. Chandrasekhar, S. Randel, G. Raybon, A. Adamiecki, P. J. Pupalaikis, and P. J. Winzer, ”All-Electronic 100-GHz Bandwidth Digital-to-Analog Converter Generating PAM Signals up to 190 Gbaud,” J. Lightwave Technol. **35**(3), 411-417 (2017).

[9] K. Schuh, F. Buchali, W. Idler, T. A. Eriksson, L. Schmalen, W. Templ, L. Altenhain, U. Dümler, R. Schmid, M. Möller, and K. Engenhardt, ”Single Carrier 1.2 Tbit/s Transmission over 300 km with PM-64 QAM at 100 Gbaud,” Proc. Optical Fiber Comm. Conf. (OFC), paper Th5B.5 (2017).

[10] P. W. Berenguer, M. Noelle, L. Molle, T. Raman, A. Napoli, C. Schubert, and J. K. Fischer, ”Nonlinear Digital Pre-distortion of Transmitter Components,” J. Lightwave Technol. **34**(8), 1739-1745 (2016).

[11] R. N. McDonough and A. D. Whalen,” *Detection of Signals in Noise*, Academic (1995).

[12] M. R. McKay, I. B. Collings, and A. M. Tulino,”Achievable Sum Rate of MIMO MMSE Receivers: A General Analytic Framework,” IEEE Trans. Inf. Theory **56**(1) 396-410 (2010).

[13] A. Napoli, B. Berenguer, T. Rahman, G. Khanna, M. M. Mezghanni, L. Gardian, E. Riccardi, A. Chiado Piat, S. Calabro, S. Dris, A. Richter, J. K. Fischer, B. Sommerkorn-Krombholz, and B. Spinnler, ”Digital Pre-Compensation Technqiues Enabling High-Capacity Bandwidth Variable Transponders,” Opt. Commun. **409**(15), 52-65 (2018).

[14] S. K. Barton and Y. O. Al-Jalili, ”A Symbol Timing Recovery Scheme Based on Spectral Redundancy,” Proc.IEE Colloquium on Advanced Modulation and Coding Techniques for Satellite Communications (1992).

[15] Y. Sato, ”A Method of Self-Recovering Equalization for Multilevel Amplitude-Modulation Systems,” IEEE Trans. Commun. **23**(6), 679-682 (1975).

[16] N. Benvenuto and G. Cherubini, *Algorithms for Communications Systems and Their Applications*, Wiley (2002).

[17] C. Füllner, A. Sherifaj, T. Henauer, D. Fang, D. Drayß, W. Freude, C.Koos, and S. Randel, ”160-GBd Photonic-Electronic PAM Transmitter Based on IQ Multiplexing,” Proc. OptoElectronics and Communications Conference/International Conference on Photonics in Switching and Computing (OECC/PSC), paper MB2-2 (2022).

[18] S. Almonacil, H. Mardoyan, F. Jorge, F. Pittalà, M. Xu, B. Krüger, F. Blache, B. Duval, L. Chen, Y. Yan, X. Ye, A. Ghazisaeidi, S. Rimpf, Y. Zhu, J. Wang, M. Goix, Z. Hu, M. Duthoit, M. Gruen, X. Cai, and J. Renaudier,

"260-GBaud Single-Wavelength Coherent Transmission over 100-km SSMF based on Novel Arbitrary Waveform Generator and Thin-Film Niobate I/Q Modulator," J. Lightwave Technol. **41**(12), 3674 - 3679 (2023).

[19] X. Chen, J. Cho, G. Raybon, D. Che, KW Kim, E. Burrows, P. Kharel, C. Reimer, K. Luke, L. He, and M. Zhang, "Single-Wavelength and Single-Photodiode 700 Gb/s Entropy-Loaded PS-256-QAM and 200-GBaud PS-PAM-16 Transmission over 10-km SMF," Proc. European Conf. on Optical Comm. (ECOC), paper Th3A.2 (2020).

[20] T. Henauer, A. Sherifaj, C. Füllner, W. Freude, S. Randel, T. Zwick, and C. Koos, "200 GBd 16QAM Signals Synthesized by an Actively-Phase-Stabilized Optical Arbitrary Waveform Generator (OAWG)," Proc. Optical Fiber Comm. Conf. (OFC), paper M2I.2 (2021).

[21] J. G. Ziegler and N. B. Nichols, "Optimum Settings for Automatic Controllers," Trans. Am. Soc. Mech. Eng. **64**, 759-768 (1942).

[22] P. D. Welch, "The Use of Fast Fourier Transform for the Estimation of Power Spectra: A method Based on Time Averaging Over Short, Modified Periodograms," IEEE trans. audio electroacoust. **15**(70) (1967).

[23] V. Bajaj, F. Buchali, M. Chagnon, S. Wahls, and V. Aref, "Deep Neural Network-Based Digital Pre-Distortion for High Baudrate Optical Coherent Transmission," J. Lightwave Technol. **40**(3), 597-606 (2022).

[24] D. Drayss, D. Fang, C. Füllner, G. Lihachev, T. Henauer, Y. Chen, H. Peng, P. Marin-Palomo, T. Zwick, W. Freude, T. J. Kippenberg, S. Randel, and C. Koos, "Non-sliced optical arbitrary waveform measurement (OAWM) using soliton microcombs," Optica **10**(7), 888-896 (2023).

[25] Keysight, "M8194A 120 GSa/s Arbitrary Waveform Generator Version 1.0," (2023). [Online]. Available: https://www.keysight.com/de/de/assets/7018-06341/data-sheets/5992-3361.pdf

[26] Keysight, "Infiniium UXR-Series Oscilloscopes Data Sheet," (2024). [Online]. Available: https://www.keysight.com/us/en/assets/7018-06242/data-sheets/5992-3132.pdf

[27] E. Ip, "Optical Coherent Detection and Digital Signal Processing of Channel Impairments," in *Handbook of Optical Fibers*, G.-D. Peng (ed.), Springer Nature Singapore Pte Ltd., ch. **I-4**, 47–217 (2019).

[28] F. Buchali, V. Aref, R. Dischler, M. Chagnon, K. Schuh, H. Hettrich, A. Bielik, L. Altenhain, M. Guntermann, R. Schmid, and M. Möller, "128 GSa/s SiGe DAC Implementation Enabling 1.52 Tb/s Single Carrier Transmission," J. Lightwave Technol. **39**(3), 763-770 (2021).

[29] IEEE Standards Association, "IEEE Standard for Terminology and Test Methods of Digital-to-Analog Converter Devices," IEEE Std 1658-2011 (2012).

[30] Plugtech Precision Systems Ltd., "Ultra Compact DP-IQ Modulator Bias Controller MBC-DPIQ-02," (2023). [Online]. Available: http://plugtech.hk/data/documents/MBC-DPIQ-ENG_20201225.pdf

[31] Md. S. Faruk and S. J. Savory, "Digital signal processing for coherent transceivers employing multilevel formats," J. Lightw. Technol. **35**(5), 1125-1141 (2017).

[32] D. Drayss, D. Fang, A. Sherifaj, H. Peng, C. Füllner, T. Henauer, G. Lihachev, W. Freude, S. Randel, T. Kippenberg, T. Zwick, and C. Koos, "Optical Arbitrary Waveform Generation and Measurement (OAWG/OAWM) Enabling 320 GBd 32QAM Transmission," Proc. Conference on Lasers and Electro-Optics (CLEO), paper STh5C.8 (2023).

[33] T. Richter, E. Palushani, C. Schmidt-Langhorst, R. Ludwig, L. Molle, M. Nölle, and C. Schubert, "Transmission of Single-Channel 16-QAM Data Signals at Terabaud Symbol Rates," J. Lightwave Technol. **30**(4), 504-511 (2011).

[34] J. B. Anderson, F. Rusek, and V. Öwall, "Faster-Than-Nyquist Signaling," Proc. IEEE **101**(8), 1817-1830 (2013).

[35] D. Che and X. Chen, "Faster-Than-Nyquist Signaling up to 300-GBd PAM-4 and 570-GBd OOK Suitable for Co-Packaged Optics," Proc. European Conf. on Optical Comm. (ECOC), paper Th3C2.6 (2021).

[36] W. Heni, B. Baeuerle, H. Mardoyan, F. Jorge, J. M. Estaran, A. Konczykowska, M. Riet, B. Duval, V. Nodjiadjim, M. Goix, J.-Y. Dupuy, M. Destraz, C. Hoessbacher, Y. Fedoryshyn, H. Xu, D. L. Elder, L. R. Dalton, J. Renaudier, and J. Leuthold, "Ultra-High-Speed 2:1 Digital Selector and Plasmonic Modulator IM/DD Transmitter Operating at 222 GBaud for Intra-Datacenter Applications," J. Lightwave Technol. **38**(9), 2734-2739 (2020).

[37] M. Nakamura, M. Nagatani, T. Jyo, F. Hamaoka, M. Mutou, Y. Shiratori, H. Wakita, and T. Kobayashi, "Over 2-Tb/s Net Bitrate Single-Carrier Transmission Based on 130-GHz-Bandwidth InP-DHBT Baseband Amplifier Module," Proc. European Conf. on Optical Comm. (ECOC), paper Th3C.1 (2022).

[38] M. Nakamura, F. Hamaoka, M. Nagatani, Y. Ogiso, H. Wakita, H. Yamazaki, T. Kobayashi, M. Ida, H. Nosaka, and Y. Miyamoto, "192-Gbaud Signal Generation Using Ultra-Broadband Optical Frontend Module Integrated with Bandwidth Multiplexing Function," Proc. Optical Fiber Comm. Conf. (OFC), paper Th4B.4 (2019).

[39] M. Nagatani, H. Wakita, H. Yamazaki, Y. Ogiso, M. Mutoh, M. Ida, F. Hamaoka, M. Nakamura, T. Kobayashi, Y. Miyamoto, and H. Nosaka, "A Beyond-1-Tb/s Coherent Optical Transmitter Front-End Based on 110-GHz-Bandwidth 2:1 Analog Multiplexer in 250-nm InP DHBT," IEEE J. Solid-State Circuits **55**(9), 2301-2315 (2020).

[40] F. Hamaoka, M. Nakamura, M. Nagatani, H. Wakita, H. Yamazaki, T. Kobayashi, H. Nosaka, and Y. Miyamoto, "Electrical Spectrum Synthesis Technique using Digital Pre-Processing and Ultra-Broadband Electrical Bandwidth Doubler for High-Speed Optical Transmitter," Electron. Lett. **54**(24), 1390-1391 (2018).

[41] F. Hamaoka, M. Nakamura, M. Nagatani, T. Kobayashi, A. Matsushita, H. Wakita, H. Yamazaki, H. Nosaka, and Y. Miyamoto, "120-GBaud 32QAM Signal Generation Using Ultra-Broadband Electrical Bandwidth Doubler," Proc. Optical Fiber Comm. Conf. (OFC), paper M2H.6 (2019).

[42] X. Chen, S. Chandrasekhar, P. J. Winzer, P. J. Pupalaikis, I. Ashiq, A. Khanna, A. Steffan, and A. Umbach, "180-GBaud All-ETDM Single-Carrier Polarization Multiplexed QPSK Transmission over 4480 km," Proc. Asia Communications and Photonics Conference (ACP), paper As4A.1 (2016).

[43] Keysight, "M8199B 256 GSa/s Arbitrary Waveform Generator Version 1.1," (2023). [Online]. Available: https://www.keysight.com/de/de/assets/3122-1928/data-sheets/M8199B-256-GSas-Arbitrary-Waveform-Generator.pdf

[44] S. Lischke, A. Peczek, J. S. Morgan, K. Sun, D. Steckler, Y. Yamamoto, F. Korndörfer, C. Mai, S. Marschmeyer, M. Fraschke, A. Krüger, A. Beling, and L. Zimmermann, "Ultra-Fast Germanium Photodiode with 3-dB Bandwidth of 265 GHz," Nat. Photon. **15**, 925-931 (2021).

[45] S. Koepfli, M. Eppenberger, Md S.-B. Hossain, M. Baumann, M. Doderer, M. Destraz, P. Habegger, E. De Leo, W. Heni, C. Hoessbacher, B. Baeuerle, Y. Fedoryshyn, and J. Leuthold, ">500 GHz Bandwidth Graphene Photodetector Enabling Highest-Capacity Plasmonic-to-Plasmonic Links," Proc. European Conf. on Optical Comm. (ECOC), paper Th3B.5 (2022).

[46] P. Runge, F. Ganzer, J. Gläsel, S. Wünsch, S. Mutschall, and M. Schell, "Broadband 145 GHz Photodetector Module Targeting 200 GBaud Applications," Proc. Optical Fiber Comm. Conf. (OFC), paper M2A.1 (2020).

[47] S. Muehlbrandt, A. Melikyan, T. Harter, K. Köhnle, A. Muslija, P. Vincze, S. Wolf, P. Jakobs, Y. Fedoryshyn, W. Freude, J. Leuthold, C. Koos, and M. Kohl, "Silicon-plasmonic internal-photoemission detector for 40 Gbit/s data reception," Optica **3**(7), 741-747 (2016).

[48] T. Harter, S. Muehlbrandt, S. Ummethala, A. Schmid, S. Nellen, L. Hahn, W. Freude, and C. Koos, "Silicon–plasmonic integrated circuits for terahertz signal generation and coherent detection," Nat. Photon. **12**, 625-633 (2019).

[49] C. Kieninger, Y. Kutuvantavida, D. L. Elder, S. Wolf, H. Zwickel, M. Blaicher, J. N. Kemal, M. Lauermann, S. Randel, W. Freude, L. R. Dalton, and C. Koos, "Ultra-high electro-optic activity demonstrated in a silicon-organic hybrid modulator," Optica **5**(6), 739-748 (2018).

[50] C. Eschenbaum, A. Mertens, C. Füllner, A. Kuzmin, A. Schwarzenberger, A. Kotz, G. Ramann, M. Chen, J. Drisko, B. Johnson, J. Zyskind, J. Marcelli, M. Lebby, W. Freude, S. Randel, and C. Koos, "Thermally stable silicon-organic hybrid (SOH) mach-Zehnder modulator for 140 GBd PAM4 transmission with sub-1 V drive signals," Proc. European Conf. on Optical Comm. (ECOC), paper Th3B.2 (2022).

[51] C. Kieninger, C. Fuellner, H. Zwickel, Y. Kutuvantavida, J. N. Kemal, C. Eschenbaum, D. L. Elder, L. R. Dalton, W. Freude, S. Randel, and C. Koos, "Silicon-organic hybrid (SOH) Mach-Zehnder modulators for 100 GBd PAM4 signaling with sub-1dB phase-shifter loss," Opt. Express **28**(17), 24693-24707 (2020).

[52] A. Graell i Amat and L. Schmalen, "Forward Error Correction for Optical Transponders," in *Springer Handbook of Optical Networks, 1st ed.*, B. Mukherjee, I. Tomkos, M. Tornatore, P. J. Winzer, and Y. Zhao (eds.), Springer, ch. **7**, 177-257 (2020).

[53] Keysight, "Infiniium UXR-B Series Oscilloscopes," (2023). [Online]. Available: https://www.keysight.com/de/de/assets/3123-1313/data-sheets/Infiniium-UXR-B-Series-Oscilloscopes.pdf

[54] P. F. Velleman and D. C. Hoaglin, *Applications, Basics, and Computing of Exploratory Data Analysis, 2nd ed.*, Duxbury Press (1981).

[55] C. R. Doerr, "Proposed Architecture for MIMO Optical Demultiplexing Using Photonic Integration," IEEE Photon. Technol. Lett. **23**(21), 1573-1575 (2011).

[56] A. Oppenheim and H. Aihara, *Discrete-Time Signal Processing, 2nd ed.*, Prentice-Hall (1999).

[57] C. R. S. Fludger, "Digital Signal Processing for Coherent Transceivers in Next Generation Optical Networks," Proc. European Conf. on Optical Comm. (ECOC), paper Tu3.1.1 (2014).

[58] H. Bülow, W. Baumert, H. Schmuck, F. Mohr, T. Schulz, F. Küppers, and W. Weiershausen, "Measurement of the maximum speed of PMD fluctuation in installed field fiber," Proc. Optical Fiber Comm. Conf. (OFC), paper WE4 (1999).

[59] C. Wang, M. Zhang, X. Chen, M. Bertrand, A. Shams-Ansari, S. Chandrasekhar, P. J. Winzer, and M. Loncar, "Integrated Lithium Niobate Electro-Optic Modulators Operating at CMOS-Compatible Voltages," Nature **562**, 101-104 (2018).

[60] M. Xu, Y. Zhu, F. Pittala, J. Tang, M. He, W. C. Ng, J. Wang, Z. Ruan, X. Tang, M. Kuschnerov, L. Liu, S. Yu, B. Zheng, and X. Cai, "Dual-Polarization Thin-Film Lithium Niobate In-phase Quadrature Modulators for Terabit-per-Second Transmission," Optica **9**(1), 61-62 (2022).
