# Supplementary material for: Photonic-electronic arbitrary-waveform generation using quadrature multiplexing and active optical-phase stabilization
Source: Nat Commun. 2025 Sep 18;16:8318. doi: 10.1038/s41467-025-61564-w (PMC12446453; doi:10.1038/s41467-025-61564-w)
Supplement: Supplementary file 3 — Transparent Peer Review file [file 41467_2025_61564_MOESM3_ESM.pdf]

nature portfolio

Peer Review File

Photonic-Electronic Arbitrary-Waveform Generation Using Quadrature Multiplexing and Active Optical-Phase Stabilization

Corresponding Author: Professor Christian Koos

This file contains all reviewer reports in order by version, followed by all author rebuttals in order by version.

Attachments originally included by the reviewers as part of their assessment can be found at the end of this file.

Version 0:

Reviewer comments:

Reviewer #1

(Remarks to the Author)
This manuscript has presented a photonic-electronic arbitrary waveform generator (PE-AWG) based on photonic quadrature modulation using two half-speed electronic digital-to-analog converters (DACs). The broadband digital-to-analog converters are essential for next generations of optical communication systems. Therefore, this manuscript came in a timely manner. The concept is well described. The performance of the PE-AWG is well characterized using the signal to noise and distortion ratio (SNDR). The analysis of the sources of degradation and the comparison to commercially available electronic AWG in the supplementary information is very detailed and useful to understand the PE-AWG methodology. Both digital and analog phase stabilization schemes are discussed and presented. And the preprocessing and postprocessing DSPs are designed in a way to reveal the true performance of the PE-AWG and are well described. To be honest, this manuscript excels in technical details and analysis, and the performance of the PE-AWG is very impressive in terms of the bandwidth and ENOB.
I would like to recommend this manuscript be published in Nature Communications. But I have a few concerns and comments that I would like to take this opportunity to discuss with the authors, before the manuscript can be published.
1. The authors name their ultra-broadband generator PE-AWG. But the characterization, analysis, and experiment are done by generating broadband on-off keying or pulse-amplitude modulation signals. I would like to see the performance of the PE-AWG with a two-tone signal generation, as a starting point of characterize an AWG. I get the idea of the generation of a broadband signal is way difficult than sine signals and SNDR based characterizations can reflect the performance with the detailed analysis the authors made in the supplementary information. However, it would be more convenient to use a much simpler signal generation, for example, a two-tone signal, to see any linear or nonlinear distortions and residual noise of the AWG. In addition, to name the generator an AWG instead of a photonic-assisted digital-to-analog converter, some two-tone sine signal generation is probably a necessity and a good starting point. Like the introduction section pointed out, some ultra-short events in scientific experiments can be emulated using an ultrafast AWG. But the short events to be emulated do not necessarily to be an ultra-broadband OOK or PAM signal with Nyquist shaping.
Please at least comment this somewhere in the paper and elaborate any potential issues, for example, postprocessing DSP (esp. adaptive equalization) is not possible for arbitrary waveform generation like a two-tone sine signal.
2. I would against the authors to claim 200 GBd PAM signal generation in the abstract, since the BER is not under the FEC threshold, as shown in Figure 4d. The claim should be 190 GBd instead.
3. The ADC and DAC in the PSCC module in Figure 1a are labeled wrong. It should be an ADC after BPD2, instead of a DAC. And before the phase shifter (PS), it should be a DAC instead of ADC. I would also recommend using different filling patterns for the solid and dashed portions of the spectra to better illustrate the evolvement of the spectra in Figure 1a and 1c.
4. The bandwidth scaling by spectral stitching is only discussed in the main text with Figure 1d, not implemented. It’s nice to have the discussion of locking all the phases using cascaded PSCCs. But there are other issues such as equalization of the slices. I would suggest putting the bandwidth scaling to the supplementary information.
5. The authors used a DC based active optical phase stabilization scheme, where the reference tone is put to the edge of the signal spectrum so that a DC component is generated after heterodyne detection and used for locking the phase of the two optical signals. However, some unwanted components, most significantly, Csin(delta_phi(t)), at DC would affect the component of interest, i.e., the LO-RT beat note, S_{RT}sin(delta_phi(t)). I wonder why not use a pilot-tone based control

mechanism so that the unwanted components at DC can be blocked. For example, put the RT with a 4-kHz offset to the edge of the signal spectrum? Then a digital filter can extract the LO-RT beat note at 4-kHz and use it as the error signal.
6. How long the memory is for the Hilbert transformation in the digital synthesis of the IQ signals?
7. The PE-AWG concept is described with photonic integration in mind. However, the implementation of this PE-AWG is based on discrete devices in this work. Although some parts for an integrated version of the PE-AWG are discussed in S2.1. But it would be a good idea to further discuss potential pros and cons of integrating everything on the same chip. For example, usually, IQ modulators work in the linear region and the result is a higher loss. Considering the coupling losses and the sensitivity of the on-chip BPDs, I guess on-chip amplification is needed. Although there are some demonstrations very lately in literatures on on-chip amplification, it is very preliminary.
8. The sampling rate of the DSO should be mentioned in the main text in Section 7.
9. The definition of LOSPR is defined in the supplementary information, not in the main text in Section 7.1.
10. It is not clearly stated whether the BER values presented in Section 7.2 are measured or derived from the SNDR.
11. I would suggest to briefly state the reason of why an analog phase-control implementation works better, in section 7.2. I can find the reason (shorter loop length) in the supplementary information, but it's nice to have in the main text.
12. It is vague to state that the LMS equalizer (can) compensates the residual chromatic dispersion in Section 8, since the transmission system is IM/DD. Does the author mean, the envelope of the transfer functon(incl. photodetection) of the residual dispersion is compensated, and the whole signal spectrum is within the main lobe of the transfer function?
13. The authors claimed that the performance of the transmission can be improved by a nonlinear predistortion of the PAM signal in section 8. I don't know if this is feasible or not, since the ENOB of the PE-AWG is only 3.7 bits.
14. Why not show eyediagrams of the PAM/OOK signals, even if the eyediagrams are oversampled results of the 1-sps equalized signals after receiver DSP?
15. Figure S3b, is the initialization process of the phase lock loop included in the first 20ms results from the output signals of the BPD2? What is the length of the digital and the analog control loops?
16. It is not clear how the distortion spectra are obtained for example in Figure S4 and S5, i.e., how was the output/intended waveforms set for the PE-AWG? For example, is the green curve in Figure S5, i.e., the oscilloscope noise, derived from subtracting the received 1-sps symbols with the expected symbols?
17. Is the remaining difference between the blue and red traces in Figure S5 correspond to the "misc" in Table S1? I can not find it in page S17.
18. There's a reference missing in the caption of Figure S6.

Reviewer #2

(Remarks to the Author)
This paper describes a method of effectively doubling the bandwidth of a given Digital to Analog Converter (DAC) technology by combining the outputs of two DACs. Rather than interleaving their outputs in time, an optical signal with two independent sidebands is created (using a complex optical modulator), and this is received using a heterodyne coherent receiver with a local oscillator (LO) placed at the lower edge of the lower sideband (or upper edge of the upper sideband). The receiver thus creates an electrical signal with the same bandwidth as the optical signal, but shifted upwards by half its width. The LO is generated by shifting the original laser's tone upwards using an intensity modulator and filter (a serrodyne frequency shifter is mentioned as an alternative).

The system is demonstrated using digital data, and an intensity modulated direct-detection system running at 200 Gbaud is constructed by taking the electrical output of the above system. The performance is plotted against commercial and near-commercial DACs, and other techniques.
This is a very well written paper with a large amount of precise detail, especially in the Supplementary Section, where I found the detailed characterizations of the performance of the system that I desired.

The system uses two optical paths: one for the frequency-shifted and modulated laser, and a direct path for the LO. The direct path actually includes optical phase control, to ensure the output is from the Inphase output of the coherent receiver. The Quadrature output is used for the phase control.

This manuscript reminds me of previous work on utilising the bandwidth of two DACs to create a single-sideband optical signal, which was then mixed with an offset carrier, to create a doubled-bandwidth electrical signal. It the previous work, only one optical path would be required, and so no phase control, and a far-simpler optical arrangement and only a single modulator [1]. The innovation there was to use the main modulator (Complex or IQ modulator) to create the two independent sidebands symmetrically around the original laser tone, f1, as in this paper; however, the LO was a shifted version of f1. This was easily created using the same modulator, by adding sine and cosine signals to the Inphase and Quadrature drives of the complex modulator (using resistive power combiners), to perform serrodyne frequency shifting. A single photodiode was used to mix the shifted-LO with the optical signal, which of course creates SSBI unless methods to move the SSBI out of the signal bandwidth are used, or the carrier is strong, or Kramers-Kronig reception is used. This SSBI was cancelled using balanced receivers and a self-coherent receiver, though [2]. The outcome was the same, in that the bandwidths of two modulators were used to create a doubled-bandwidth electrical signal. It could be argued that the LO in this case was really a "carrier"; however, this technique was later used in a self-coherent experiment, where the carrier was input to the LO input of a coherent receiver. Fig.4 of [2] is even closer to the present manuscript, apart from the frequency shifter being in the LO path rather than the signal path.

So, the manuscript is original, in that it's an alternative technique. However, its claim in the Summary: "the first of its kind and (that?) allows synthesizing arbitrary optical waveforms with twice the bandwidth of electronic digital-to-analog converters

(DACs)" is not entirely true if the "and" is supposed to imply a "that". I certainly this that it should acknowledge some of the history of the field [1,2].

That said, does this technique offer any advantage over the previous technique? The separate path for the LO may avoid a polarization controller required for the coherent-receiver version of the previous technique. Are there other advantages? The combining of many bands is similar to the coherent OFDM "superchannel" ideas, [3], for example.

In conclusion, I think if some history is acknowledged, then this MS makes a good contribution to the field.

Some smaller comments:
Fig. S6 has some additional result from recent industry DACs. These seem to go against the claim in Fig. 3's caption that "… is on par with commercially available waveform generators, while featuring a much higher bandwidth". Indeed, the Supplementary section explicitly states this, but is hidden from people who won't read the "small print".

Fig 3 caption. "eletrical"

Fig. 2: +1, -1 +j -j. These real-world signals (in wires) are usually called Inphase and Quadrature, to avoid confusion with mathematical constructs which are partly imaginary.

Fig. 1a. These isometric drawings of non-real things are just annoying as the visual cortex has preferred directions. They make me want to bend my neck in an impossible way! The mention of wire bonding in the text is almost fooling me into think that you made it, and is unnecessary, because you could equally describe the whole "imaginary" fabrication process.

A lot of the theory is just RF single sideband generation. That is, if you drive a complex modulator (two mixers with a 90-deg. shift in one path) with an analytic signal (a signal and its Hilbert transform), you get a single sideband, to which you can add a carrier. Of course, there has been a lot of interest in optical SSB, particularly for DD systems. Is this part of the story, particularly as you demonstrate an IM/DD system (though it is IM not field modulated?)?

References

B. J. C. Schmidt, A. J. Lowery and L. B. Du, "Low sample rate transmitter for direct-detection optical OFDM," 2009 Conference on Optical Fiber Communication, San Diego, CA, USA, 2009, pp. 1-3, doi: 10.1364/OFC.2009.OWM4

B. J. C. Schmidt, Z. Zan, L. B. Du and A. J. Lowery, "120 Gbit/s Over 500-km Using Single-Band Polarization-Multiplexed Self-Coherent Optical OFDM," in Journal of Lightwave Technology, vol. 28, no. 4, pp. 328-335, Feb.15, 2010, doi: 10.1109/JLT.2009.2033613

A. Li, X. Chen, G. Gao and W. Shieh, "Transmission of 1 Tb/s Unique-Word DFT-Spread OFDM Superchannel Over 8000 km EDFA-Only SSMF Link," in Journal of Lightwave Technology, vol. 30, no. 24, pp. 3931-3937, Dec.15, 2012, doi: 10.1109/JLT.2012.2206369.

Reviewer #3

(Remarks to the Author)
This manuscript, as an extension from the conference papers [23] and [24], presents an "photonic-electronic (PE) AWG" employing an electronic-optical-electronic (E-O-E) method of generating arbitrary baseband electronic waveforms beyond the limitations of electronic DACs. In the proposed system, an optical IQM is utilized as an electro-optical multiplexer that combines two baseband waveforms, each with a bandwidth of B, to generate an optical waveform with a total bandwidth of 2B around the optical carrier. The output waveform of the IQM is then combined with a coherent LO light with a frequency adjusted to the edge of the spectrum of that waveform. Finally, the waveform is downconverted to the baseband through mixing with the LO in a PD. The relative optical phase between the IQM's output waveform and the LO is stabilized by an opto-electronic PLL. A balanced configuration is employed to suppress SSBI.
In total, the manuscript is well-written. The underlying principle is elaborated in detail, making it comprehensible even to readers outside the field. Extensive experimental results are provided to validate the idea.
However, the motivation remain unclear, and several technical aspects raise concerns. The following points require clarification and additional support:

1. What are the expected applications of this PE-AWG? For applications using optical waveforms themselves as the output [1-8, 10], an OAWG [21] is clearly more suitable because there are no need for adding an extra O-E-O conversion for such applications. Also for the wireless systems, the optical signals are directly downconverted to the intermediate frequency [10], so the downconversion to the baseband is not required. Test and measurement of high-speed electronics may be a possible application, but aren't there anything else? The authors need to provide a more in-depth discussion of their motivation and potential applications for this technology.

2. The proposed system can be understood as an interesting derivative of the setup used in some optical SSB transmission experiments including the same author's previous study [47] and those cited in it, where an optical IQM is used to generate signals with a total bandwidth twice that of the DACs, and a frequency shifter or a separate laser is used to add a "carrier" at the edge of the IQM's output spectrum. The authors should mention this point in Section 2.

3. The discussion of bandwidth scaling in Section 5 is questionable. Although some advanced PDs are reported with extremely high bandwidths [9, 30, 32], it will not be easy to use their outputs without any trans-impedance amps, which will limit the final bandwidth of the PE-AWG. If such extremely-broadband RF amps were to be developed in the future , other electronic devices would likely have achieved similar capailities, making the fully-electronic approaches comparable to the PE-AWG. Packaging and connectorization will also be challeing issues. The authors should also discuss these points in Section 5. The scaling concept as shown in Fig. 1(d) is reasonable for OAWG, but simply applying it to the PE-AWG may not be appropriate.

4. Were the swing voltages mentioned in Section 7 and Fig. 3 measured after the linear RF amp? The authors should clalify this point.

In particular, point 1 must be clearly addressed for publlication, as it was not sufficietly explained in the previous conference proceedings. The reviewer understands the experimental demonstration of the optical PAM transmission using the PE-AWG is just for proof of concept, but it is simply confusing to see the setup including E-O-E-O-E conversions, which are clealy redundant for obtaining similar transmission performances. While practical aspects may not be the primary concern of this journal, since this technology is essentially a combination of known technologies with originality lying in their application, further discussion on this point is indispensable.

Reviewer #4

(Remarks to the Author)
The manuscript described a photonic-electronic arbitrary-waveform generator (PE-AWG) which has the potential to compete with commercial expensive electronic instrument. The concept of quadrature multiplexing of optical waveforms is new and up to 200 GBd signal generation has been demonstrated using discrete components.

The most highlight of the work is the use of optical quadrature multiplexing to effectively double the limited bandwidth in electronics. The design of the system is sound, and experimental results are promising. Another novelty is the closed-loop active phase stablization which is crucial for the success of the work.

However there are several unclarities preventing the impact of the work:
1. The gap between vision in Fig. 1a and actual system is large. Fiber and discrete components were used for the first system demo. It was successful, but it addresses very different challenges than those integrated on chip. The challenges of integrating the system on chip was not thought of, and experiments did not orient to address those.
One example is the reflections and scattering among optical (and electronic) components on chip. The magnitude and distance and density of those parasitic effects on chip can be completely different from those in fiber based systems.
Another example is the use of EDFA for loss balance. On-chip loss balance / compensation is also essential for complex system on chip (such as integrated optical switches). Semiconductor optical amplifiers were used, but they have a completely different noise characteristics as EDFA.
So the gap between Fig 1a and Fig 2a is large and not addressed. I wonder, have those been considered and at least at system level been modelled for their impacts? If experiments were designed to address the gap, it will make the paper stronger.

2. Scalability is also not crystal clear and not convincing. Fig 1d presented authors' vision (although far from reaching it) on future scaling of the proposed AWG concept using spectral stitching. The scaling of IQM and PSCC is clear from the graph, but the scaling of BPD is confusing.
The Fig 1d only shows how four channels (f2 - f5) are combined into one channel and finally merged with LO and fed into BPD1. But how do other channels do? Do the authors intend to group every 4 frequencies and use one BPD for that group? Or do the authors want to eventually combine all channels into one BPD? The way Fig1d is illustrated can have so many different interpretations.
Also, how is a single LO distributed over so many channels? Not addressed.

3. The closed loop active phase control is interesting. It was a necessity in the system demo mainly because of the phase which is subject to "random drift over time due to vibrations and/or temperature fluctuations..... especially in fiber-optic devices". It is an elegant and effective design, but it is so far only justifiable to fiber-optic systems. It is unclear why a fully integrated system on chip would need the same (see Fig 1a for the PSCC).
In chip scale system, one would expect the phase errors due to temperature and mechanical instabilities to be much better controlled. Also because of the chip integration, even those instabilities exist, their effects should be much weaker as the optical path lengths are orders of magnitude shorter. So the question is, how is the chip-integrated PSCC justified in authors' vision?

4. From Fig 3d, the Keysight M8199B outperforms the proposed system by almost 5dB in SNDR before 180 GBd. The authors explained that the new system was not available at the time of measurement. But it is unclear what would the authors do, if the new system was known earlier to them? Will there be new experiments, and/or improvement to the system architecture, etc, to bring the SNDR to a similarly high level? Or is it M8199B's fundamental advantage due to its new SiGe technology?

5. Another observation in Fig 3d is that M8199B's SNDR declines very rapidly beyond 180GBd. While the proposed system has a much more "flat" trend towards higher rates. If just judged by simple extrapolation, it seems that the proposed system

may outperform the most advanced M8199B for beyond 200GBd?
More analysis, discussion and insights into the trends would be highly valuable.

OVerall I believe it is a very solid experiment. The concept has a high potential. But the impact is not fully visible, and there is an unaddressed gap of feasibility between current demo and authors' vision.

Version 1:

Reviewer comments:

Reviewer #1

(Remarks to the Author)
The authors have addressed all my concerns and comments to a very satisfactory level. The new experiment and detailed analysis on a two-tone signal is deeply appreciated and show many technical details that the audience of this paper could benefit from. New indicator for Fig. 3(d) for the 7% FEC threshold and the new Fig. S7 also answer my doubts of the achieved BER with 200 GBd PAM4 modulation. Many other additional information and clarifications are also provided for the bandwidth scaling, active phase stabilization, signal processing, and potential improvements for an integrated version of the PE-AWG. This paper could serve as an important technical reference for high-speed signal generation, characterization, and optical communication. I would recommend the paper to be published in Nature Communications in a timely manner, as is.

Reviewer #2

(Remarks to the Author)
Please see the attached file.

Reviewer #3

(Remarks to the Author)
Thank you for addressing my previous comments with your revised submission. I am pleased to see that you have incorporated adequate explanations for all the points I raised. While I still have some reservations regarding whether the bandwidth gap between PD and DACs will widen enough to justify the introduction of optical spectral stitching, I believe this is a matter that deserves broader discussion in the field moving forward. No further comments are necessary at this stage. I believe the manuscript is now suitable for publication in this journal.

Reviewer #4

(Remarks to the Author)
The technical work itself is no doubt a interesting and valuable piece of work. It has improved even, after this revision.
The doubts on the PIC concept / feasibility and scalability, shared by all reviewers, have been addressed in a proper way.
The authors clarified the relation between their non-PIC experiment and their PIC-enabled vision. It is a pity that no quantitative analysis was provided on the PIC feasibility as this can be even more impactful, but to me, it is understandable as the focus of the work is proof of concept.
So overall I am satisfied with the revised manuscript, and I support to accept this piece of work for NC.

Open Access This Peer Review File is licensed under a Creative Commons Attribution 4.0 International License, which permits use, sharing, adaptation, distribution and reproduction in any medium or format, as long as you give appropriate credit to the original author(s) and the source, provide a link to the Creative Commons license, and indicate if changes were made.
In cases where reviewers are anonymous, credit should be given to 'Anonymous Referee' and the source.
The images or other third party material in this Peer Review File are included in the article's Creative Commons license, unless indicated otherwise in a credit line to the material. If material is not included in the article's Creative Commons license and your intended use is not permitted by statutory regulation or exceeds the permitted use, you will need to obtain permission directly from the copyright holder.
To view a copy of this license, visit https://creativecommons.org/licenses/by/4.0/
